# Higher BODIPY Homologues–Synthesis, Reactivity, and Photoluminescence Investigations

**DOI:** 10.1002/chem.202404764

**Published:** 2025-05-02

**Authors:** Lukas Erlemeier, Roman‐Malte Richter, Tobias Dunaj, Marius J. Müller, Sangam Chatterjee, Carsten von Hänisch

**Affiliations:** ^1^ Department of Chemistry and Marburg Center for Quantum Materials and Sustainable Technology (mar.quest) Philipps University Marburg Hans‐Meerwein Straße 4 35043 Marburg Germany; ^2^ Institute of Experimental Physics I and Center for Materials Research Justus Liebig University Giessen 35392 Giessen Germany

**Keywords:** BODIPY, coordination chemistry, dipyrrin, dipyrromethene, DPM, fluorescence, group 13, main group chemistry, quantum efficiency, triel

## Abstract

The synthesis of ^Mes^DPM group 13 (B─In, ^Mes^DPM = 1,5,9‐trimesityldipyrromethene) compounds with halide substituents (Cl─I) is described. All compounds were fully characterized including NMR and IR spectroscopy as well as mass spectrometry. In addition, the solid state molecular structures have been determined by X‐ray diffraction (XRD) analysis. As higher representatives of BODIPY (boron difluoride dipyrromethene) dyes, some of these ^Mes^DPM triel dihalides also exhibit an intense green fluorescence when exposed to sunlight. In this regard, the optical properties were investigated by UV/Vis and photoluminescence spectroscopy giving absorption maxima around 520 nm and fluorescence emission in the range between 550 and 660 nm. Fluorescence quantum efficiencies up to 42% could be obtained from measurements in toluene solution. Further, reactivity studies were carried out which opened‐up access to mixed substituted ^Mes^DPM triels with one alkyl and one halide substituent.

## Graphical abstract

Higher homologues of the well‐known BODIPY dyes for boron, aluminum, gallium, and indium are described. These have been obtained with the halogen substituents chlorine, bromine, and iodine and as mixed substituted compounds with methyl and halogen substituents. The optical properties are described as well as the observation of excimers.

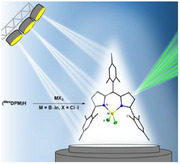

**Abbreviations**
^Mes^DPM: 1,5,9‐trimesityl‐dipyrromethene
DPM: Dipyrromethene
BODIPY: Boron difluoro dipyrromethene
Mes: 2,4,6‐trimethylphenyl
Dipp: 2,6‐di*iso*propylphenyl
Trip: 2,4,6‐tri*iso*propylphenyl
GM: General Method

## 1 Introduction

Investigating the luminescent properties of materials containing light main group elements has become a wide‐spreading part of current research with respect to optoelectronic applications.^[^ ^1^, ^2^, ^3^ ^]^ In this regard, several types of luminescent light main group compounds can be found in the literature, including carbene complexes of Li or Mg,^[^ ^1^ ^]^ Si nanocrystals,^[^ ^1^ ^]^ alkali metal iminophosphoamides,^[^ ^3^ ^]^ azomethines^[^ ^2^ ^]^ as well as *β*‐ketoiminates, respectively, diketo/diketiminates of B and Al.^[^ ^2^ ^]^

**Figure 1:**
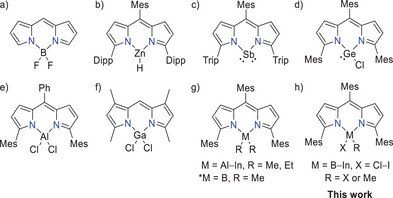
Lewis formula of “parent” BODIPY (a), selected literature known DPM complexes of the transition metal Zn (b), of main group elements with low‐valent antimony (c) and divalent germanium (d) as well as a DPM aluminum and gallium halide complexes (e, f), our previous works (g), and this work (h, *).

In the last decades, particularly dipyrromethene (DPM)‐based compounds, like boron difluoride dipyrromethene (BODIPY) (Figure 1a),^[^ ^4^, ^5^, ^6^ ^]^ gained great attention due to their highly fluorescent properties leading to many different applications, for example in the fields of fluorescent switches, light‐harvesting arrays, or as fluorescence labels in biomedical imaging and photo dynamic therapy.^[^ ^7^, ^8^, ^9^, ^10^, ^11^, ^12^, ^13^, ^14^, ^15^, ^16^, ^17^ ^]^ Ligand based π*‐*π* emissions are described as a source of the fluorescent behavior of DPM‐based compounds. Hereby, the strength of the electronic transition is influenced by the dimension of the conjugated *π*‐system, the bulkiness of the aryl group at the *meso*‐position, as well as the rigidification generated by chelating a cationic species.^[^ ^18^, ^19^, ^20^, ^21^ ^]^ In recent years, especially the interest in BODIPY‐type DPM complexes with transition metals and heavier main group elements has grown immense.^[^ ^22^, ^23^, ^24^, ^25^, ^26^, ^27^, ^28^, ^29^, ^30^ ^]^ For example, the C─H bond activation ability of several DPM Fe and Co complexes was intensively studied by the *Betley* group^[^ ^23^, ^24^ ^]^ while a catalytic CO_2_ hydrosilylation was shown by Ballmann et al. using a Zn hydride DPM (Figure 1b).^[^ ^22^ ^]^ With respect to main group elements, Liu et al. reported a bulky DPM stabilized low‐valent antimony compound (Figure 1c) with the potential of cleaving disulfides and diselenides.^[^ ^25^ ^]^ Further, the *Nagendran* group used the sterically demanding 1,5,9‐trimesityldipyrromethene (^Mes^DPM) ligand for the stabilization of divalent germanium (Figure 1d) while Su et al. reported the synthesis of DPM‐supported radical species of germanium or the triels boron to gallium.^[^ ^26^, ^28^, ^29^ ^]^ Investigations of the luminescent properties of higher group 15 analogues of BODIPY were carried out by Bismuto et al.^[^ ^27^ ^]^ Similar studies on the synthesis of DPM complexes with the heavier main group elements arsenic and tin have been published recently.^[^ ^30^ ^]^ In our earlier studies, we reported the easy and high yielding synthesis of strongly fluorescent ^Mes^DPM triel dialkyls of the type [(^Mes^DPM)MR_2_] (M = Al─In; R = Me, Et; Figure 1g).^[^ ^21^ ^]^ However, despite the huge number of BODIPY‐based compounds and relatives, which can be found in the literature, the amount of its directly higher homologues is quite low. In this regard, the *Mason* group described the synthesis and reactivity studies of aluminum dihalide containing DPM compounds (Figure 1e)^[^ ^31^ ^]^ while Wan et al. observed photoluminescent behavior for gallium dichloride chelated by a 1,3,7,9‐tetramethyl‐ dipyrromethene ligand (Figure 1f).^[^ ^32^ ^]^ Recently, also the *Harder* group reported the synthesis of [(*^R^* DPM)MI_2_] (R = *t*Bu, M = Al, Ga; R = Mes, M = Ga), and in case of M = Ga of the K@KI mediated reduction to generate a DPM stabilized low‐valent gallium compound.^[^ ^33^, ^34^ ^]^ But as far as our knowledge, reports of DPM complexes of both, higher triels and heavier halides as well, are so far rare to find in the literature. This is surprising, because it was shown that a substitution of second row elements (as in BODIPY's) by heavier main group elements can have dramatic impacts on a molecule's optical and electronic properties.^[^ ^35^, ^36^, ^37^, ^38^, ^39^ ^]^

In this context, we herein present the facile synthesis and characterization of higher group 13 dihalide complexes with the sterically demanding ^Mes^DPM ligand which makes these compounds heavier and bulkier homologues of the well‐known BODIPY. In addition, the optical properties of these compounds were intensively studied. Moreover, we examined reactivity studies to investigate reversible reaction pathways between the ^Mes^DPM triel dihalides and the dialkyl species reported recently.^[^ ^21^ ^]^

## 2 Results and Discussion

**Scheme 1:**
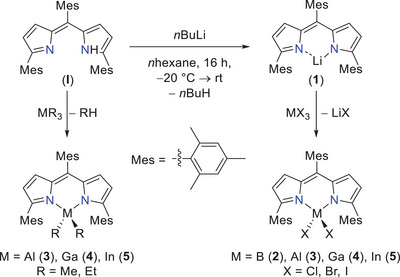
Synthesis of lithiated ligand (^Mes^DPM)Li (**1**) starting from (^Mes^DPM)H (**I**) as well as following alkane eliminations (left) to literature known ^Mes^DPM triel dialkyls resp. salt eliminations (right) to the desired group 13 ^Mes^DPM dihalides.

The synthesis of the starting compound, the free (protonated) ligand (^Mes^DPM)H (**I**), occurs according to the literature by an acid‐catalyzed condensation reaction of mesitaldehyde dimethyl acetal and two equivalents of 2‐Mes‐1H‐pyrrole (using PPTS ‍ = ‍pyridinium *p*‐toluenesulfonate as catalyst) followed by a 2,3‐dichloro‐5,6‐dicyano‐1,4‐benzoquinone (DDQ) mediated oxidation.^[^ ^24^, ^40^, ^41^ ^]^ On the one hand, **I** can be treated with group 13 trialkyls giving the corresponding [(^Mes^DPM)MR_2_] triel compounds **3_R2_**, **4_R2,_** and **5_R2_** (**3** = Al, **4** = Ga, **5** = In; R = Me, Et; Scheme 1, left) in quantitative yields, as we have reported previously.^[^ ^21^ ^]^ However, corresponding treatment of **I** with BEt_3_ was shown to not yield in the formation of the desired compound **2_Et2_** (**2** = B). On the other hand, compound **I** can further be lithiated. According to synthetic routes known from the literature, lithiation usually is carried out in coordinating solvents such as ethers,^[^ ^20^, ^24^, ^42^ ^]^ resulting in the generation of corresponding solvent adducts. In contrast, when performing the synthesis in a noncoordinative solvent such as alkanes or toluene, the solvent free [(^Mes^DPM)Li] (**1**) can be obtained after drying under reduced pressure in quantitative yield (Scheme 1, top). Despite attempts on determining the XRD structure of the solvent free compound failed so far, we were able to discover the molecular structure in solid state of its THF adduct, which was missing in the literature to date (see the Supporting Information). After lithiation, compound **1** can further undergo salt metathesis reactions with the group 13 trihalides generating the desired ^Mes^DPM triel dihalides **2_X2_**, **3_X2_**, **4_X2,_** and **5_X2_** (except **2_I2_**) as red/orange solids in moderate yields between 47% and 89% (Scheme 1 right) analogue to the recently published synthesis of **4_I2_** by the *Harder* group.^[^ ^34^ ^]^ Comparing the alkane elimination pathway to **3_Me2_**, **4_Me2,_** and **5_Me2_** mentioned above^[^ ^21^ ^]^ with the salt eliminations route to **2_X2_**, **3_X2_**, **4_X2,_** and **5_X2_**, the latter one leads to a significantly lower yield as the syntheses are associated with further purification steps to separate the resulting lithium halide. Based on this, the question arose as to whether both classes of DPM compounds could be converted into each other in order to receive higher yields for **3_X2_**, **4_X2,_** and **5_X2_** resp. to find a new synthetic route to the missing boron compounds **2**_Me2_ or **2**_I2_.

**Scheme 2:**
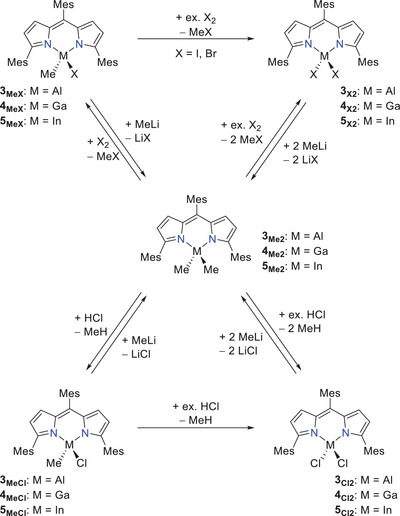
Reactivity studies of [(^Mes^DPM)MMe_2_] (**3**–**5_Me2_**) toward halides resp. HCl into the mixed substituted compounds **3**–**5_MeX_** (X = Cl─I) as well as ^Mes^DPM triel dihalides **3**–**5_X2_**.

In this regard, we started our reactivity studies by reacting the dihalides **2_X2_**, **3_X2_**, **4_X2,_** and **5_X2_** with two equivalents of MeLi which generates the expected dimethyl compounds **2_Me2_**, **3_Me2_**, **4_Me2,_** and **5_Me2_** in almost quantitative yields (Scheme 2). For the reverse case, reactions of alkyl compounds with iodine is a common way to transfer alkyl into iodide substituents, as was shown, e.g., by Ballmann et al. for the synthesis of [(^Mes^DPM)ZnI], by Cui et al. for [({HC(CMeNDipp)_2_}AlI_2_)], or by Richter et al. for synthesis of [(*^t^* ^Bu^DPM)AlI_2_].^[^ ^22^, ^33^, ^43^ ^]^ Here, the reaction of **3_Me2_**, **4_Me2,_** and **5_Me2_** with up to two equivalents I_2_ surprisingly results in the selective generation of the mixed substituted compounds [(^Mes^DPM)M(Me)I] (M = Al (**3_MeI_**), Ga (**4_MeI_**), or In (**5_MeI_**); Scheme 2 top left). This observation could be proved by mass spectrometry as well as NMR spectroscopic measurements. The latter reveal an unsymmetrically coordination of the metal center as was shown by the appearance of a second set of signals for the flanking mesityl substituents at the ligand backbone. The resulting mixed substituted products **3_MeI_**, **4_MeI,_** and **5_MeI_** were obtained as red/pink solids with quantitative yields. In contrast, for the generation of the desired compounds **3_I2_**, **4_I2,_** and **5_I2_** a significantly higher excess of I_2_ of up to four equivalents is needed (Scheme 2 top right; see Supporting Information for more details). Considering these results, performing same reactions but with using bromine instead of iodine under similar conditions surprisingly do not yield in the selective formation of the expected products (neither **3**–**5_Br2_** nor **3**–**5_MeBr_**) but to a product mixture due to the high reactivity of bromine. Nevertheless, decreasing the reaction temperature down to − 60 °C, finally lead to the mixed bromide species **3_MeBr_** and **5_MeBr_**. In case of **4_MeBr_**, synthesis was performed via vapor diffusion of bromine into a stirred solution of **4_Me2_** in toluene. To obtain the appropriate chloride compounds **3_Cl2_**, **4_Cl2,_** and **5_Cl2_** as well as the mixed substituted species **3_MeCl_**, **4_MeCl,_** and **5_MeCl_** starting from **3_Me2_**, **4_Me2,_** and **5_Me2_**, a 4 M HCl solution in 1,4‐dioxane was used as more harmless alternative to chlorine gas (Scheme 2d). The reaction with one equivalent of HCl lead to the selective formation of the mixed substituted ^Mes^DPM triels **3_MeCl_**, **4_MeCl,_** and **5_MeCl_** as red/pink solids in quantitative yield. Further, an increase to a higher excess of HCl yields the dihalide compounds **3_Cl2_**, **4_Cl2,_** and **5_Cl2_** also in quantitative yields. All compounds can be stored in inert atmosphere as solid or in toluene solution even at elevated temperatures (90 °C). In addition, no light sensitivity was observed for any compound during the 24‐month duration of our work. However, on contact with moisture, hydrolysis occurs under release of the protonated ligand (**I**).

## 3 Optical Properties

**Figure 2:**
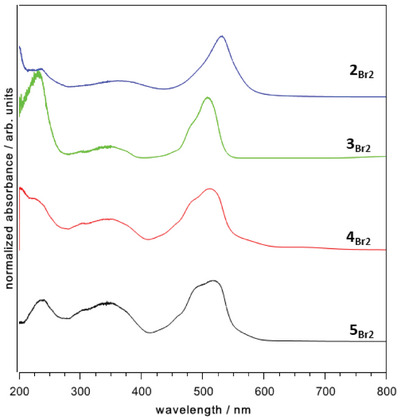
Normalized and stacked solid state UV/Vis spectra of synthesized ^Mes^DPM triel dibromides (color scheme: blue = **2_Br2_**; green ‍ = **3_Br2_**; red = **4_Br2_**; black ‍ = ‍**5_Br2_**).

**Figure 3:**
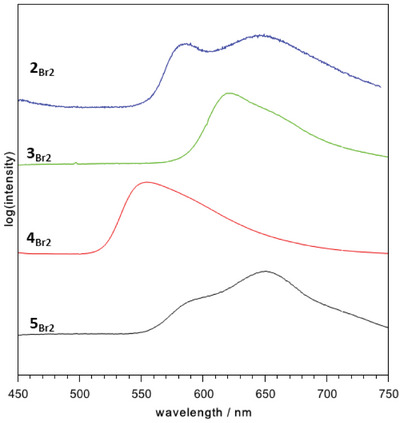
Normalized and stacked photoluminescence spectra of synthesized ^Mes^DPM triel dibromides (color scheme: blue = **2_Br2_**; green ‍ = **3_Br2_**; red = **4_Br2_**; black ‍ = ‍**5_Br2_**). Excitation with 405 nm continuous wave diode laser.

In our earlier studies on ^Mes^DPM triel dialkyls we described intense green fluorescence in solution under visible light irradiation, particularly for **3_Me2,_** and **4_Me2_**. Similar observations became evident during the synthesis of several ^Mes^DPM triel dihalides. For this reason we investigate the optical properties of **2**–**5_X2_** as well as **3**–**5_MeX_** using UV/Vis spectroscopy as well as photoluminescence experiments. UV/Vis spectroscopic measurements in solid state of **2**–**5_X2_** (Figure 2) exhibit several and predominately low intense absorptions in the ultraviolet region (200–380 nm) which are discussed as metal‐perturbed intraligand charge transfer. In addition, maximum absorptions *λ*_max,ss_ in the range from 507 to ‍532 nm can be observed. This small deviation of about 20 nm is in good agreement with the low influence of the coordinated metal atom as was shown by calculations for **3**–‍**5_R2_**.^[^ ^21^ ^]^ The values also show a slightly hypsochromic shift with increasing period of the halide substituents. Comparing the received data of the measurements in solid state and in toluene solution, the latter show a slight blue field shift of approximately 10 nm giving maximum absorptions *λ*_max_ around 510 nm. Using the maximum absorptions we further were also able to determine the molar extinction coefficients for **2**–**5_X2_** with values between 0.48 and 1.49 ×10^5^ L•mol^−1^•cm^−1^.These results align well with the dialkyl species **3**–**5_R2_** ^[^ ^21^ ^]^ and to other compounds of this class.^[^ ^8^, ^25^, ^26^, ^31^, ^44^ ^]^ Comparing the obtained data with the mixed substituted compounds **3**–**5_MeX_**, similar absorption maxima were observed from the measurements in both, solid state and in toluene solution. Nevertheless, significant higher molar extinction coefficients *ε*_max_ between 0.91 and 1.72 × 10^5^ L•mol^−1^•cm^−1^ were determined. Furthermore, the influence of halide substitution on the fluorescence response of ^Mes^DPM triel dihalides was investigated by performing room temperature photoluminescence spectroscopy in toluene solution (Figure 3). The data reveal emission maxima in the green area of the visible spectrum within a span of 550 nm to 660 nm (π*→*π* transitions). In line with expectations, for **3**–**5_MeX_** fluorescence emission occurs at higher energies (550–600 nm, except from **3_MeI_** at 619 nm) with maxima localized between those observed for the ^Mes^DPM triel dialkyl and dihalide species. With view on the spectra, high fluorescence intensities for the gallium compounds **4_X2_** as well as the boron compound **2_Cl2_** became evident which reflects the largest quantum efficiencies within this product class with values between 14% and 22% for **4_X2_** and 42% for **2_Cl2_** (Table 1). Compared to the BODIPY compounds as well as their aluminum analogues, the gallium compounds exhibit stronger *φ*_F_ values in this series. The higher quantum yield of the gallium compounds compared to the aluminum compounds is surprising. We attribute this effect to the higher covalency of the Ga─N bonds compared to the Al─N bonds. The Ga─N bonds are thus more strongly directed than the predominantly ionic interaction between ligand and aluminum. As a result, the GaX_2_ (X = Cl, Br, I) groups lead to a greater stiffening of the ligand, which is conducive to high fluorescence.

**Table 1:** UV/Vis and photoluminescence data for [(^Mes^DPM)BMe_2_](**2_Me2_**) and the ^Mes^DPM triel halides [(^Mes^DPM)MX_2_] (**2**–**5_X2_**) and [(^Mes^DPM)M(Me)X] (**3**–**5_MeX_**) with M = B─In and X = Cl─I.

Compound	*λ*_max,ss_ [nm]	*λ*_max_ [nm]	*ε*_max_ (λ_max_) [L•mol^−1^•cm^−1^]	*λ*_F_ [nm]	*φ*_F_ [%]
[LBMe_2_] (**2_Me2_**)	510	515	0.13•10^5^	538, 649	1.8
[LBCl_2_] (**2_Cl2_**)	528	524	0.69•10^5^	572	42
[LBBr_2_] (**2_Br2_**)	532	529	0.48•10^5^	590, 649	1.0
[LAlCl_2_] (**3_Cl2_**)	519	510	1.20•10^5^	594, 657	0.6
[LAlBr_2_] (**3_Br2_**)	517	512	0.87•10^5^	650	0.3
[LAlI_2_] (**3_I2_**)	518	510	1.25•10^5^	644	0.02
[LGaCl_2_] (**4_Cl2_**)	512	508	1.30•10^5^	560	19
[LGaBr_2_] (**4_Br2_**)	510	509	1.02•10^5^	554	22
[LGaI_2_] (**4_I2_**)	517	512	1.02•10^5^	566	14
[LInCl_2_] (**5_Cl2_**)	509	505	1.49•10^5^	613, 655	0.4
[LInBr_2_] (**5_Br2_**)	507	505	1.02•10^5^	623	0.3
[LInI_2_] (**5_I2_**)	513	508	0.57•10^5^	622	0.2
[LAl(Me)Cl] (**3_MeCl_**)	516	509	1.06•10^5^	587	0.6
[LGa(Me)Cl] (**4_MeCl_**)	506	508	1.73•10^5^	554	39
[LIn(Me)Cl] (**5_MeCl_**)	513	504	1.17•10^5^	592	0.9
[LAl(Me)Br] (**3_MeBr_**)	518	511	1.21•10^5^	553	29
[LGa(Me)Br] (**4_MeBr_**)	511	510	0.95•10^5^	553	24
[LIn(Me)Br] (**5_MeBr_**)	510	506	0.91•10^5^	571	6.1
[LAl(Me)I] (**3_MeI_**)	524	514	1.05•10^5^	619	0.2
[LGa(Me)I] (**4_MeI_**)	521	514	1.08•10^5^	565	0.5
[LIn(Me)I] (**5_MeI_**)	512	508	1.03•10^5^	560	0.6

Abbreviations: L = ^Mes^DPM, *λ*_max,ss_ = absorption maximum (solid state), *λ*_max_ = absorption maximum (toluene solution), *ε*_max_ (*λ*_max_) = molar absorption coefficient (at *λ*_max_), *λ*_F_ = fluorescence emission maximum, *φ*_F_ = fluorescence quantum efficiency

A comparison of the fluorescence lifetimes within a halide series (Cl, Br, I) provides deeper insights into the nature of the excited states. Exemplary data for the triel gallium are presented in the Supporting Information. The compounds exhibit fluorescence half‐life times of 4.1 ns, 3.7 ns, and 1.2 ns for **4_Cl2_**, **4_Br2_**, and **4_I2_**, respectively. These data infer decreasing values with the atomic order of the halide substituents. This observation appears to contradict the initial assumption of the occurrence of phosphorescence and much rather infers favored nonradiative intersystem crossings into the singlet ground state with increasing atom number of the respective halides.

Moreover, in the series of the mixed substituted, the highest fluorescence quantum yields were obtained for **4_MeCl_** with 39% as well as for **3**–**4_MeBr_** with about 29% resp. 24%.

Except from **5_MeBr_** (6.1%) all other compounds show comparably low fluorescence intensities with *φ*_F_ values below 1%. It stands out that drastically fluorescence quenching occurs predominantly for indium coordination as well as iodine substitution attributable to the heavy atom effect. In general, the investigations revealed a decreasing fluorescence quantum efficiencies with increasing degree of halide substitution. For example, while for **4_Me2_** a *φ_F_* value of 51%^[^ ^21^ ^]^ was still observed, this value already fell to 39% for the monosubstituted compound **4_MeCl_** and even further down to 19% in case of the disubstituted species **4_Cl2_**.

**Figure 4:**
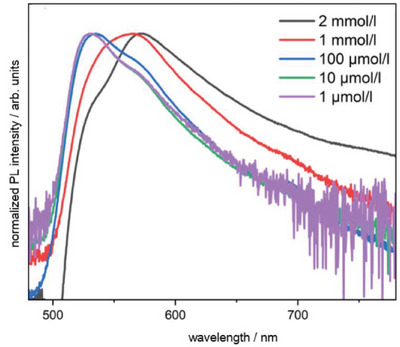
Normalized photoluminescence intensity of [(^Mes^DPM)BBr_2_] (**2_Br2_**) solutions in toluene (color scheme: black = 2 mmol, red = 1 mmol, blue = 100 µM, green = 10 µM, purple = 1 µM). Excitation: frequency doubled Ti:Sapphire Laser (400 nm, 78 MHz, 100 fs).

In case of **2_Br2_**, **3_Cl2_** as well as **5_Cl2_**, the appearance of two emission bands indicate excimer formation. To confirm this assumption, exemplarily for **2_Br2_**, concentration dependent UV/Vis as well as photoluminescence experiments were performed. The appearance of a single absorption band in the UV/Vis experiments even at higher concentrations (up to 2 mM) proves that the emissions are not the result of two different absorptions by **2_Br2_**. Rather, the photoluminescence experiments of a series of concentrations revealed a progressive disappearance of the lower energy emission with decreasing concentrations so that the remaining signal can be assigned to the monomer emission (Figure 4).

**Figure 5:**
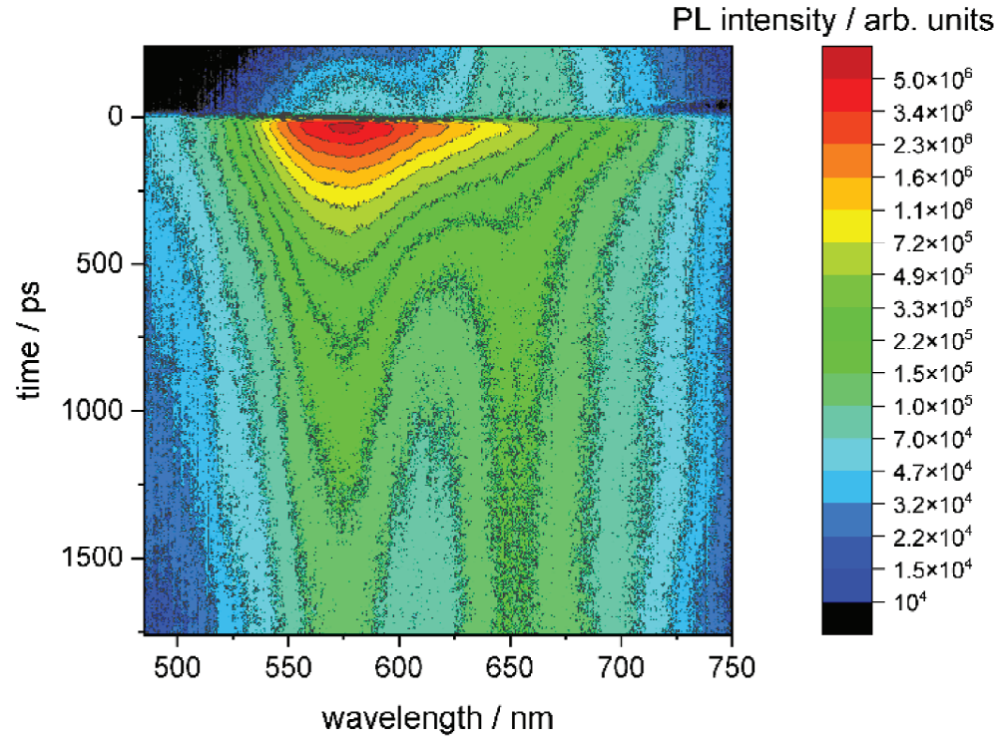
Time‐resolved photoluminescence of a 2 mM **2_Br2_** toluene solution. Excitation: frequency doubled Ti:Sapphire Laser (400 nm, 78 MHz, 100 fs).

Last but not least, fluorescence lifetime experiments (Figure 5) have shown a difference in excitation lifetime between the two emission maxima of **2_Br2_**. While the higher energetic state was shown to have a short‐termed lifetime of 84 ps, the fluorescence emission at higher wavelengths revealed a significantly longer duration, which can be best described using a biexponential model with two lifetimes of 124 ps and 9.8 ns (see Supporting Information).

## 4 X‐Ray Diffraction (XRD) Analysis

**Figure 6:**
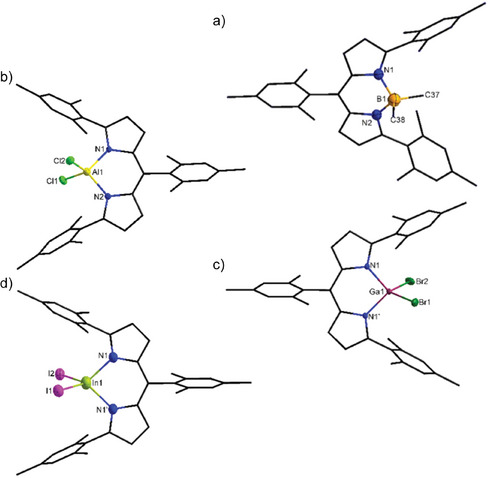
Exemplary solid state molecular structures of a) [(^Mes^DPM)BMe_2_] (**2_Me2_**), b)‍ [(^Mes^DPM)AlCl_2_] (**3_Cl2_**), c) [(^Mes^DPM)GaBr_2_] (**4_Br2_**), and d) [(^Mes^DPM)InI_2_] (**5_I2_**) with thermal ellipsoids set at the 50% probability level. Carbon atoms are depicted as wireframe and hydrogen atoms are omitted for clarity.

As a result of the reactivity studies, finally the solid state molecular structure of [(^Mes^DPM)BMe_2_] (**2_Me2_**) could be determined which was missing in our earlier studies due to failed syntheses via the alkane elimination route.^[^ ^21^ ^]^ Compared to the higher homologues **3**–**5_Me2_** which crystallize in the monoclinic crystal system, **2_Me2_** crystallizes triclinic with the space group *P* $\bar{1}$, instead (Figure 6a). According to the smaller atomic radius of boron compared to the higher homologues, the solid state molecular structure of **2_Me2_** reveal significantly shorter bond lengths. However, these values are in good agreement with literature data.^[^ ^5^ ^]^ For **2_Me2_**, therefore a distorted tetrahedral coordination sphere around the boron center with *τ*_4_ & *τ*_4_’ values of 0.93 is present.

**Table 2:** Selected bond lengths *d*_A‐B_ and angels ∢_A‐B‐C_ as well as *τ*_4_ & *τ*_4_’ values for **2**–**5_X2_** as well as for **2_Me2_**.

Compound	*d*_M_─_N_ [Å]	*d*_M_─_X_ [Å]	∢_N_─_M_─_N_ [°]	*τ*_4_ *& τ*_4_’
**2_Me2_**	1.600(3) 1.598(3)	1.601(3) 1.632(4)	103.4(2)	0.93
**2_Cl2_**	1.542(6) 1.543(5)	1.875(5) 1.825(5)	107.2(3)	0.95
**3_Cl2_**	1.881(2) 1.887(2)	2.109(1) 2.118(1)	98.1(1)	0.96
**4_Cl2_**	1.913(5) 1.935(5)	2.147(2) 2.160(1)	97.3(2)	0.96
**5_Cl2_**	2.128(2)	2.3323(8) 2.3341(7)	89.80(9)	0.94
**2_Br2_**	1.537(4) 1.534(4)	1.980(4) 2.093(4)	108.5(3)	0.94
**3_Br2_**	1.890(1)	2.2779(8) 2.2747(8)	97.85(9)	0.95
**4_Br2_**	1.934(1)	2.2963(3) 2.3025(3)	96.65(6)	0.94
**5_Br2_**	2.130(2)	2.4666(4) 2.4702(3)	89.48(8)	0.93
**3_I2_**	1.901(5)	2.500(3) 2.510(3)	97.6(3)	0.94
**4_I2_**	1.944(1)	2.5138(3) 2.5183(3)	96.37(8)	0.94
**5_I2_**	2.145(3)	2.6722(4) 2.6734(5)	88.9(2)	0.92

Furthermore, the synthesized ^Mes^DPM triel dihalides **2**–**5 _X2_** were crystallized from toluene at room temperature or −32 °C and the solid state structures determined by XRD analysis (Figure 6b–d). The ^Mes^DPM triel dihalides predominantly crystallizes isotypically in the monoclinic crystal system with the space group *P*2_1_/*m*. Herein, a mirror plane passing through the mesityl ring at the *meso*‐position of the ^Mes^DPM ligand reveals the symmetrical coordination of the triel atom, which was confirmed by NMR spectroscopic measurements. Exceptions from these are **2_Cl2_** and **2_Br2_** that crystallizes in *P*2_1_/*c* as well as the compounds **3_Cl2_** and **4_Cl2_** which differs from these by crystallizing in the space group *P*2_1_. In case of boron, the small atomic radius leads to a strong distortion of the aromatic backbone. When considering the central metal atoms, a distorted tetrahedral coordination sphere with *τ*_4_ and *τ*_4_’ values close to 1 (≥0.92) can be observed, while the distortion increases with the atomic order from aluminum to indium as well as from chlorine to iodine (Table  2). Corresponding to the atomic radii, the compounds also exhibit increasing bond lengths around the metal center as the period of the halide or metal atom increases, while the N─M─N bond angles decrease. However, the halide substitution has no significant impact on the structural metrics of the metal center. A comparison with the methyl substituted ^Mes^DPM triels **3_Me2_**, **4_Me2,_** and **5_Me2_** reveals slightly lower M─N bond lengths for the ^Mes^DPM triel dihalides. Nevertheless, all obtained bond lengths and angles are in good agreement with literature data.^[^ ^21^, ^31^, ^32^, ^33^ ^]^

**Figure 7:**
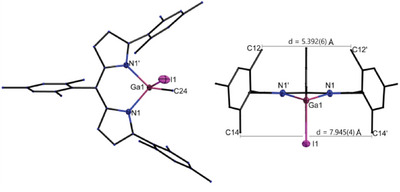
Exemplary solid state molecular structure of [(^Mes^DPM)Ga(Me)I] (**4**_MeI_) with thermal ellipsoids set at the 50% probability level. Carbon atoms are depicted as wireframe and hydrogen atoms are omitted for clarity.

Further, we were able to analyze the solid state molecular structures of the mixed substituted compounds **3**–**5_MeX_** (exemplarily shown for **4_MeI_** in Figure 7), which proves the different substitution of the metal centers. All of the compounds crystallize isotypically to **3**–**5_X2_** (X = Br, I) and **3**–**5_Me2_** in the monoclinic crystal system with the space group *P*2_1_/*m*. However, in several cases the asymmetrical substitution leads to a disorder between the triel centered methyl group and the halide substituent. With view on the solid state molecular structure especially for **4_MeI_** a movement of the metal atom out of the aromatic plane of the ligand backbone became evident. Contrary to the dihalides **3**–**5_X2_**, this causes a rotation of the two mesityl substituents towards the side of the alkyl group (e.g., **4_MeI_**: *d*_C12–C12’_ = Figure 7, right) providing more space for the halide substituent (*d*_C14–C14’_ = 7.945(4) Å) and explaining the further splitting of the mesityl signals within the ^1^H NMR spectra for these compounds. In line with expectations, the molecular structures reveal similar bond lengths and angles compared to the ^Mes^DPM triel dialkyls **3**–**5_Me2_** or the dihalide compounds **3**–**5_X2_** mentioned previously (Table 3). In addition, comparing the result shown here with literature known DPM triel compounds, the values reveal no huge differences.^[^ ^5^, ^21^, ^31^, ^32^, ^33^ ^]^

**Table 3:** Selected bond lengths *d*_A‐B_ and angels ∢_A‐B‐C_ as well as *τ*_4_ and *τ*_4_’ values for **3**–**5_MeX_**.

Compound	*d*_M─N_ [Å]	*d*_M─C_ [Å]	*d*_M─X_ [Å]	∢_N─M─N_ [°]	*τ*_4_ *& τ*_4_’
**3**_MeI_	1.908(7)	1.93(2)	2.557(4)	96.7(4)	0.85
**4**_MeI_	1.950(2)	1.952(5)	2.5736(5)	94.0(1)	0.85
**5**_MeI_	2.149(7)	2.07(2) 2.13(2)	2.678(1) 2.524(9)	87.4(4)	0.89
**3**_MeBr_	1.909(2)	1.999(4)	2.306(1)	96.1(2)	0.91
**4**_MeBr_	1.955(3)	1.959(6)	2.358(1)	94.2(2)	0.85
**5**_MeBr_	2.154(3)	2.222(8)	2.4951(8)	87.1(2)	0.88
**3**_MeCl_	1.907(2)	1.996(4)	2.138(2)	95.7(1)	0.92
**4**_MeCl_	1.951(2)	1.932(4)	2.196(1)	94.47(9)	0.85
**5**_MeCl_	2.153(1)	2.143(3)	2.3781(7)	87.09(7)	0.85

## 5 Conclusion

In order to build up a library of heavier BODIPY homologues to gain deeper understanding into the photoluminescence behavior of such compounds, the ^Mes^DPM triel dihalides **2**–**5_X2_** were synthesized via salt metathesis reactions. Moreover, performing reactivity studies with these compounds, our studies revealed not only access to the so far missing compound **2_Me2_** but also to a new class of DPM‐based triel complexes with two different substituents at the metal center. With these compounds some more examples of rarely represented DPM supported indium compounds are reported.

With the overall aim on studying the influence of metal coordination and substitution within the DPM core on the optical properties of resulting compounds, the present work confirmed a negligible effect of different triel coordination on the absorption maxima of ^Mes^DPM triel halides. Moreover, investigations on the photoluminescence behavior of our compounds revealed fluorescence emissions in the green area of the electromagnetic spectrum. Thereby, comparably high fluorescence quantum yields up to 42% could be determined from measurements in toluene solution at room temperature. On the one hand, our studies confirmed the positive effect of gallium coordination on the fluorescence intensity of DPM compounds as already stated in our earlier works. On the other hand, drastically fluorescence quenching was observed for DPM compounds with participating heavy atoms. With these values, we were able to demonstrate a correlation between halide substitution on the fluorescence quantum efficiencies. In addition, the observation of two emission bands in case of **2_Br2_**, **3_Cl2_** as well as **5_Cl2_** led to the suggestion of excimer formation which was supported by concentration dependent UV/Vis and photoluminescence measurements as well as fluorescence lifetime experiments exemplarily for **2_Br2_**.

With regard on tailoring the photoluminescent behavior of such compounds, our actual research targets the alternative synthesis of higher ^Mes^DPM triel fluorides in direct contrast to BODIPY. In addition we search for new classes of heavier BODIPY homologues in order to improve their optical properties for the development of new fluorescence dyes for practical application. Further, using the iodine compounds, studies on singlet oxygen generation with regard to photo dynamic therapy will be part of future research.

## 6 Experimental Section

Below are the synthesis instructions for all the compounds described here, summarized where appropriate. Full characterization details can be found in the Supplementary Information.

**1**: At room temperature 7.50 g (^Mes^DPM)H (**I**, 15.04 mmol, 1.00 eq.) was suspended in 200 mL toluene under stirring. To this stirred suspension 6.54 mL of a 2.3 M solution of *n*BuLi (15.04 mmol, 1.00 eq.) in hexane was added dropwise at ^−^20 °C. The reaction mixture was allowed to warm up to room temperature and was stirred for 16 hours. Evaporation of the solvent under reduced pressure and trituration with *n‐*pentane gives the desired [(^Mes^DPM)Li] (**1**) as a red solid in quantitative yield.

**2_Me2_**: At room temperature 0.25 g [(^Mes^DPM)BCl_2_] (**2_Cl2_**, 0.44 mmol, 1.00 eq.) were dissolved in 5 mL toluene under stirring. To this, 0.28 mL of a 3.1 M solution of MeLi in DEM (0.87 mmol, 2.00 eq.) was added dropwise. The reaction mixture was stirred at room temperature for 1 day. The obtained suspension was filtered through a celite padded frit and the residue washed with 1 mL toluene. The filtrate was evaporated and triturated with *n‐*pentane (three times with each 2 mL). The residue was dried under reduced pressure to afford 0.18 g [(^Mes^DPM)BMe_2_] (**2_Me2_**, 0.33 mmol) as a red/orange solid in 75% yield (alternative synthesis by use of **2_Br2_** instead of **2_Cl2_** also possible).

### General method A

**3**–**5_X2_**: At room temperature, 0.10 g **1** (0.20 mmol, 1.00 eq.) were dissolved in 10 mL toluene under stirring. To this, MX_3_ (M = Al─In; X = Cl─I) (0.24 mmol, 1.20 eq.) was added. The reaction mixture was stirred at room temperature for 2 days. The obtained suspension was filtered and the residue washed with 2 mL toluene. The filtrate was evaporated and dried under reduced pressure at 85 °C to afford the desired ^Mes^DPM triel dihalides [(^Mes^DPM)MX_2_] (**3**–**5_X2_**, M = Al─In; X = Cl─I) as red/orange solids.

### General method B

**3**–**5_MeI_**: At room temperature, [(^Mes^DPM)MMe_2_] (M = Al─In (**3_Me2_**, **4_Me2_** or **5_Me2_**), 0.20 mmol, 1.00 eq.) were dissolved in 10 mL toluene under stirring. To this, I_2_ (0.20 mmol, 1.00 eq.) was added. The reaction mixture was stirred at room temperature for 16 hours. Evaporation of the solvent and drying under reduced pressure at 85 °C affords [(^Mes^DPM)M(Me)I] (**3**–**5_MeI_**, M ‍ = ‍Al─In) as red/pink solids in quantitative yield.

### General method C

**3**–**5_MeCl_**: At room temperature, [(^Mes^DPM)MMe_2_] (M = Al─In (**3_Me2_**, **4_Me2_** or **5_Me2_**), 0.20 mmol, 1.00 eq.) were dissolved in 10 mL toluene under stirring. To this, HCl in 1,4‐dioxane (4 M, 0.20 mmol, 1.00 eq.) was added dropwise. The reaction mixture was stirred at room temperature for 16 hours. Evaporation of the solvent and drying under reduced pressure at 85 °C affords [(^Mes^DPM)M(Me)Cl] (**3**–**5_MeCl_**, M = Al─In) as red/pink solids in quantitative yield.

### General method D

**3**–**5_I2_**: At room temperature, [(^Mes^DPM)MMe_2_] (M = Al─In (**3_Me2_**, **4_Me2_** or **5_Me2_**), 0.20 mmol, 1.00 eq.) were dissolved in 10 mL toluene under stirring. To this, I_2_ (1.00 mmol, 5.00 eq.) was added. The reaction mixture was stirred at room temperature for 16 hours. Evaporation of the solvent and drying under reduced pressure at 85 °C affords [(^Mes^DPM)MI_2_] (**3**–**5_I2_**, M = Al─In,) as red/orange solids in quantitative yield.

### General method E

**2**–**5_Cl2_**: At room temperature, [(^Mes^DPM)MMe_2_] (M = Al─In (**2_Me2_**, **3_Me2_**, **4_Me2_** or **5_Me2_**), 0.20 mmol, 1.00 eq.) were dissolved in 10 mL toluene under stirring. To this, HCl in 1,4‐dioxane (4 M, 1.00 mmol, 5.00 eq.) was added dropwise. The reaction mixture was stirred at room temperature for 16 hours. Evaporation of the solvent and drying under reduced pressure at 85 °C affords (^Mes^DPM)MCl_2_ (**2**–**5_Cl2_**, M = Al─In,) as red/orange solids in quantitative yield.

**2_Cl2_**: At room temperature 0.50 g [(^Mes^DPM)Li] (**1**, 0.99 mmol, 1.00 eq.) were dissolved in 5 mL toluene under stirring. To this, 1 mL of a 1 M solution of BCl_3_ in heptane (0.99 mmol, 1.00 eq.) was added. The reaction mixture was stirred at room temperature for 2 days. The obtained suspension was filtered and the residue washed with 1 mL toluene. The filtrate was evaporated and triturated with *n‐*pentane (three times with each 2 mL). The residue was dried under reduced pressure to afford the desired [(^Mes^DPM)BCl_2_] (**2_Cl2_**) as a red solid in 83% yield (alternative synthesis via General Method E also possible (yield > 95%).

**2_Br2_**: At room temperature 0.25 g [(^Mes^DPM)Li] (**1**, 0.50 mmol, 1.00 eq.) were dissolved in 5 mL toluene under stirring. To this, 0.5 mL of a 1 M solution of BBr_3_ in toluene (0.50 mmol, 1.00 eq.) was added. The reaction mixture was stirred at room temperature for 2 days. The obtained suspension was filtered and the residue washed with 1 mL toluene. The filtrate was evaporated and triturated with *n‐*pentane (three times with each 2 mL). The residue was dried under reduced pressure to afford 295 mg of the desired [(^Mes^DPM)BBr_2_] (**2_Br2_**, 0.44 mmol) as a red solid in 89% yield.

3Cl2: General Method A: Yield: 85%; General Method E: Yield > 95%.

**4_Cl2_**: General Method A: Yield: 85%; General Method E: Yield > 95%.

**5_Cl2_**: General Method A: Yield: 85%; General Method E: Yield > 95%.

**3_Br2_**: General Method A: Yield: 81%.

**4_Br2_**: General Method A: Yield: 78%.

**5_Br2_**: General Method A: Yield: 78%.

**3_I2_**: General Method A: Yield: 78%; General Method D: Yield > 95%.

**4_I2_**: General Method A: Yield 75%; General Method D: Yield > 95%.

**5_I2_**: General Method A: Yield: 47%; General Method D: Yield > 95%.

**3_MeCl_**: General Method C: Yield: >95%.

**4_MeCl_**: General Method C: Yield: >95%.

**5_MeCl_**: General Method C: Yield: >95%.

**3_MeBr_**: At room temperature, 0.20 g [(^Mes^DPM)AlMe_2_] (**3_Me2_**, 0.36 mmol, 1.00 eq.) were dissolved in 10 mL toluene under stirring. To this, 18.5 µL Br_2_ (57.6 mg, 0.36 mmol, 1.00 eq.) dissolved in 10 mL toluene was added dropwise under stirring at −60 °C over 30 minutes. The reaction mixture was warmed up to room temperature over 8 hours and stirred at room temperature for additional 8 hours. The solvent was evaporated and the product triturated two times with each 10 mL *n‐*pentane. Drying under reduced pressure at 85 °C affords [(^Mes^DPM)Al(Me)Br] (**3_MeBr_**) as a red/pink solid in quantitative yield.

**4_MeBr_**: At room temperature, 0.20 g [(^Mes^DPM)GaMe_2_] (**4_Me2_**, 0.34 mmol, 1.00 eq.) were dissolved in 10 mL toluene under stirring. On top, a dropping funnel with pressure equalizer containing a solution of 17.1 µL Br_2_ (53.5 mg, 0.34 mmol, 1.00 eq.) dissolved in 10 mL toluene. The solution of **4_Me2_** was stirred at room temperature for 14 days. The solvent was evaporated and the product triturated two times with each 10 mL *n‐*pentane. Drying under reduced pressure at 85 °C affords [(^Mes^DPM)Ga(Me)Br] (**4_MeBr_**) as a red solid in quantitative yield.

**5_MeBr_**: At room temperature, 0.20 g [(^Mes^DPM)InMe_2_] (**5_Me2_**, 0.31 mmol, 1.00 eq.) were dissolved in 10 mL toluene under stirring. To this, 15.9 µL Br_2_ (49.7 mg, 0.31 mmol, 1.00 eq.) dissolved in 10 mL toluene was added dropwise under stirring at −60 °C over 30 minutes. The reaction mixture was warmed up to room temperature over 8 hours and stirred at room temperature for additional 8 hours. The solvent was evaporated and the product triturated two times with each 10 mL *n‐*pentane. Drying under reduced pressure at 85 °C affords [(^Mes^DPM)In(Me)Br] (**5_MeBr_**) as red/pink solid in quantitative yield.

**3_MeI_**: General Method B: Yield: >95%.

**4_MeI_**: General Method B: Yield: >95%.

**5_MeI_**: General Method B: Yield: >95%.

### XRD analysis

Single crystal XRD analysis was conducted using a StadiVari diffractometer by *STOE* with CuKα (*λ* = 1.54186) radiation (*Xenocs* Microfocus Source, *λ* ‍ = 1.54186 Å) and a *Dectris* Pilatus 300 K detector as well as on a *Bruker* D8 Quest diffractometer. The diffractometer uses Mo − Kα (λ  =  0.71073 Å, *Incoatec* Microfocus Source) radiation and a Photon 100 CMOS detector. Structures were solved via intrinsic phasing using SHELXT‐2015. Structure refinement was performed via full‐matrix‐least‐squares against *F* ^2^ using SHELXL‐2015. All structures were solved and refined using the OLEX2 platform.

Deposition numbers 2 413 317 (**1**·THF), 2 413 333 (**2_Me2_**), 2 413 316 (**2_Cl2_**), 2 413 318 (**2_Br2_**), 2 413 320 (**3_Cl2_**), 2 413 322 (**3_Br2_**), 2 413 326 (**3_I2_**), 2 413 332 (**4_Cl2_**), 2 413 329 (**4_Br2_**), 2 413 325 (**5_Cl2_**), 2 413 331 (**5_Br2_**), 2 413 335 (**5_I2_**), 2 413 337 (**3_MeCl_**), 2 413 323 (**3_MeBr_**), 2 413 321 (**3_MeI_**), 2 413 324 (**4_MeCl_**), 2 413 319 (**4_MeBr_**), 2 413 364 (**4_MeI_**), 2 413 330 (**5_MeCl_**), 2 413 328 (**5_MeBr_**), and 24 134 (**5_MeI_**), contain the supplementary crystallographic data for this paper. These data can be obtained free of charge via www.ccdc.cam.ac.uk/data_request/cif. For details of the crystal structures see also Supporting Information.

## Author Contributions

Lukas Erlemeier: project realization, synthesis, characterization, sample preparation, implementation of SC‐XRD experiments, crystal structure refinement, and writing of the manuscript. Roman‐Malte Richter: implementation of SC‐XRD experiments. Tobias Dunaj: support with the refinement of crystal structures. Marius J. Müller: measurement of photoluminescence spectra and determination of fluorescence quantum efficiencies. Sangam Chatterjee: scientific management of optical measurements and writing of the manuscript. Carsten von Hänisch: scientific management of the project and writing of the manuscript. All authors have given approval to the final version of the manuscript.

## Conflict of Interests

The authors declare no conflict of interest.

## Supporting information



Supporting Information

## Data Availability

The data that support the findings of this study are available in the supplementary material of this article.
