# Supplementary material for: Higher BODIPY Homologues–Synthesis, Reactivity, and Photoluminescence Investigations
Source: Chemistry. 2025 May 2;31(31):e202404764. doi: 10.1002/chem.202404764 (PMC12133635; doi:10.1002/chem.202404764)
Supplement: Supplementary file 1 — Supporting Information [file CHEM-31-e202404764-s001.docx]

Supplementary Information

**Higher BODIPY Homologues – Synthesis, Reactivity and Photoluminescence Investigations**

Lukas Erlemeier,^a^ Roman-Malte Richter,^a^ Tobias Dunaj,^a^ Marius J. Müller,^b^ Sangam Chatterjee,^b,*^ Carsten von Hänisch^a,^*

1. Department of Chemistry, Philipps University Marburg, 35032 Marburg, Germany. E-Mail: carsten.vonhaenisch@chemie.uni-marburg.de.
2. Institute of Experimental Physics I, Justus Liebig University Giessen, 35392 Giessen, Germany. E-Mail: sangam.chatterjee@physik.uni-giessen.de.

Content

[1. Experimental procedures: 3](#_Toc194312311)

[2. Synthesis and characterization of [(^Mes^DPM)Li] (1) 5](#_Toc194312312)

[3. Synthesis and characterization of ^Mes^DPM triel dihalides 8](#_Toc194312313)

[3.1 [(^Mes^DPM)BCl_2_] (**2**_Cl2_): 8](#_Toc194312314)

[3.2 [(^Mes^DPM)BBr_2_] (**2**_Br2_): 12](#_Toc194312315)

[3.3 [(^Mes^DPM)AlCl_2_] (**3**_Cl2_): 16](#_Toc194312316)

[3.4 [(^Mes^DPM)AlBr_2_] (**3**_Br2_): 21](#_Toc194312317)

[3.5 [(^Mes^DPM)AlI_2_] (**3_I2_**): 26](#_Toc194312318)

[3.6 [(^Mes^DPM)GaCl_2_] (**4**_Cl2_): 30](#_Toc194312319)

[3.7 [(^Mes^DPM)GaBr_2_] (**4_Br2_**): 34](#_Toc194312320)

[3.8 [(^Mes^DPM)GaI_2_] (**4**_I2_): 38](#_Toc194312321)

[3.9 [(^Mes^DPM)InCl_2_] (**5_Cl2_**): 42](#_Toc194312322)

[3.10 [(^Mes^DPM)InBr_2_] (**5**_Br2_): 46](#_Toc194312323)

[3.11 [(^Mes^DPM)InI_2_] (**5**_I2_): 50](#_Toc194312324)

[4. Synthesis and characterization of [(^Mes^DPM)BMe_2_] (2_Me2_): 54](#_Toc194312325)

[5. Synthesis and characterization of mixed substituted ^Mes^DPM triel complexes: 58](#_Toc194312326)

[5.1 [(^Mes^DPM)Al(Me)I] (**3**_MeI_): 58](#_Toc194312327)

[5.2 [(^Mes^DPM)Ga(Me)I] (**4**_MeI_): 63](#_Toc194312328)

[5.3 [(^Mes^DPM)In(Me)I] (**5**_MeI_): 67](#_Toc194312329)

[5.4 [(^Mes^DPM)Al(Me)Cl] (**3**_MeCl_): 71](#_Toc194312330)

[5.5 [(^Mes^DPM)Ga(Me)Cl] (**4**_MeCl_): 76](#_Toc194312331)

[5.6 [(^Mes^DPM)In(Me)Cl] (**5**_MeCl_): 80](#_Toc194312332)

[5.7 [(^Mes^DPM)Al(Me)Br] (**3**_MeBr_): 84](#_Toc194312333)

[5.8 [(^Mes^DPM)Ga(Me)Br] (**4**_MeBr_): 89](#_Toc194312334)

[5.9 [(^Mes^DPM)In(Me)Br] (**5**_MeBr_): 93](#_Toc194312335)

[6. Optical properties 97](#_Toc194312336)

[6.1 UV/Vis and photoluminescence spectra in toluene solution resp. solid state: 97](#_Toc194312337)

[6.2 Spectroscopic data 103](#_Toc194312338)

[7. Crystallographic data: 104](#_Toc194312339)

[7.1 [(^Mes^DPM)Li∙thf] (**1∙THF**) 104](#_Toc194312340)

[7.2 [(^Mes^DPM)BMe_2_] (**2_Me2_**) 105](#_Toc194312341)

[7.3 [(^Mes^DPM)MCl_2_] (M = B (**2_Cl2_**), Al (**3_Cl2_**), Ga (**4_Cl2_**), In (**5_Cl2_**)) 106](#_Toc194312342)

[7.4 [(^Mes^DPM)MBr_2_] (M = B (**2_Br2_**), Al (**3_Br2_**), Ga (**4_Br2_**), In (**5_Br2_**)) 107](#_Toc194312343)

[7.5 [(^Mes^DPM)MI_2_] (M = Al (**3_I2_**), Ga (**4_I2_**), In (**5_I2_**)) 108](#_Toc194312344)

[7.6 [(^Mes^DPM)M(Me)I] (M = Al (**3_MeI_**), Ga (**4_MeI_**), In (**5_MeI_**)) 109](#_Toc194312345)

[7.7 [(^Mes^DPM)M(Me)Br] (M = Al (**3_MeBr_**), Ga (**4_MeBr_**), In (**5_MeBr_**)) 110](#_Toc194312346)

[7.8 [(^Mes^DPM)M(Me)Cl] (M = Al (**3_MeCl_**), Ga (**4_MeCl_**), In (**5_MeCl_**)) 111](#_Toc194312347)

[8. Literature: 112](#_Toc194312348)

# 1. Experimental procedures:

**General synthetic considerations**:

All manipulations were performed under an inert argon atmosphere using standard *Schlenk* techniques. The handling and storage of moisture or air sensitive substances occurred under an inert argon atmosphere in a glove box. Solvents were dried by standard procedures and freshly distilled before use. 1,5,9-trimesityldipyrromethene ((^Mes^DPM)H, **1**) was prepared according to literature methods.^[1]^

**Analytic methods**:

**NMR** spectroscopic experiments including the nuclei ^1^H, ^13^C, ^27^Al and ^119^Sn occur in automation on a *Bruker* AV II 300 MHz, AV III HD 300 MHz or in manual operation on the spectrometer types *Bruker* AV III HD 300 MHz, AV III 500 MHz. Coupling constants (*J*) were reported in Hertz (Hz) and the chemical shifts (δ) are given in ppm relative to the standard (^1^H, ^13^C: SiMe_4_). The NMR multiplicities are abbreviated with s = singlet, d = doublet, t = triplet, q = quartet or m ‍= ‍multiplet. The assignment of ^13^C and ^1^H signals was made using 2D NMR spectroscopy (HMQC, COSY). The evaluation of NMR data was performed using *MestReNova* *6.0.2* by *Mestrelab* *Research*.^[2]^

**IR** spectra were recorded on a *Bruker* ALPHA FT‐IR with a diamond ATR (500‐4000 cm^‐1^) and processed using OPUS 7.2.139_Wizard. The signals are reported in cm^-1^ and assigned with br = broad and/or w = weak, m = medium or s = strong.

**CHN** (elemental analyses) experiments of the elements hydrogen, carbon and nitrogen were performed on an *Elementar* Vario Microcube and the content is reported in %.

**HR**-**CI** mass spectra were acquired with an AccuTOF GCv 4G (*JEOL*) Time of Flight (TOF) mass spectrometer. An internal or external standard was used for drift time correction.

**SC-XRD** experiments occur predominantly on a StadiVari diffractometer by *STOE* with CuKα radiation (*Xenocs* Microfocus Source, *λ* ‍= 1.54186) and a *Dectris* Pilatus 300K detector or on a D8-Quest diffractometer by *Bruker* with MoKα radiation (*Incoatec* Microfocus Source, *λ* = 0.71073) and a Photon 100 CMOS detector. In addition, for a few exceptions data were collected on a *Bruker* Kappa APEX II diffractometer equipped with an APEX II CCD detector using a TRIUMPH monochromator with a MoKα X-ray source at the *University of California Santa Barbara* (UCSB). All measurements occurred at 100 K with crystal cooling by nitrogen gas (*Oxford* *Cryosystems*). Suitable single crystals were obtained from saturated toluene solution at room temperature or –32 °C (unless specified otherwise). The integration of XRD data was carried out with *Apex3*^[3]^ or *X-Area*^[4]^. The solving and refinement of the structures occurred using *SHELXT* and *SHELXL* on the *OLEX2* application.^[5,6]^ Images of crystal structures were made using *Diamond* *4*.^[7]^ All ellipsoid plots represent the 50% probability level.

**Solid-state UV/Vis** spectroscopic measurements were measured in reflection under inert conditions employing a Varian Cary 5000 UV/Vis/NIR spectrometer from *Agilent*, equipped with a Praying Mantis accessory. **Extinction** spectra were recorded from 2 µM toluene solutions in inert atmosphere at 298.15 K on an *Analytik Jena* Specord S600 using WinASPECT software and an *UNISOKU* CoolSpeK Cryostat. Excitation and emission spectra were measured in solution on a Varian Cary Eclipse fluorescence spectrometer from *Agilent* with a xenon flash lamp.

**Photoluminescence** **quantum yield** spectroscopy was performed with 2 µM toluene solutions under inert conditions in a 1 mm thick quartz-glass cuvette with the sample mounted under direct illumination in an integrating sphere. As excitation source a 405 nm continuous wave diode laser was used. The emission was spectrally resolved with a 50 cm Czerny−Turner spectrograph and detected by a thermoelectrically cooled Si CCD camera. Corrections for setup response and integrating sphere emission were performed according to the literature.^[8]^ The maximum wavelength (*λ*_i_) of received signals from spectroscopic measurements (UV/Vis and PL) are given in nm and calculated fluorescence quantum efficiencies (*φ*_F_) are reported in %. The quantum efficiencies were validated against a solution of perylene in ethanol.^[9]^ Presented images of UV/Vis and photoluminescence spectra were made using QtiPlot.

**Time-resolved photoluminescence** spectroscopy was performed on 100 µM and 2 mM solutions in quartz-glass cuvettes with a confocal microscope. The samples were excited using a frequency doubled Ti:Sa Laser (400 nm, 78 MHz, 100 fs). The emission was spectrally resolved with a Czerny−Turner spectrograph and detected by a thermoelectrically cooled Si CCD camera, as well as temporally resolved using a streak camera. The lifetimes were fitted with exponential models accounting for the back sweep signal and additional buildup.2. Synthesis and characterization of ^Mes^DPM triel dihalides

# 2. Synthesis and characterization of [(^Mes^DPM)Li] (1)

**^1^H-NMR** (300.19 MHz, C_6_D_6_) *δ* = 6.87 (s, 2 H, -C*H*(Mes)), 6.85 (s, 4 H, -C*H*(Mes)), 6.82 (d, ^3^*J*_AB_ = 3.9 Hz, 2 H, -C*H*(py)), 6.32 (d, ^3^*J*_AB_ = 3.9 Hz, 2 H, -C*H*(py)), 2.36 (s, 6 H, -‍*Me*), 2.28 (s, 12 H, -*Me*), 2.25 (s, 3 H, -‍*Me*), 2.20 (s, 6 H, -*Me*) ppm.

**^13^C{^1^H}-NMR** (75.48 MHz, C_6_D_6_) *δ* = 159.2 (s, 2 C, *C*_quart_ (pyr)), 141.7 (s, 1 C, *C*_quart_ (Mes)), 137.2 (s, 1 C, *C*_quart_ (Mes)), 137.2 (s, 4 C, *C*_quart_ (Mes)), 137.1 (s, 2 C, *C*_quart_ (Mes)), 136.9 (s, 2 C, *C*_quart_ (Mes)), 136.6 (s, 2 C, *C*_quart_ (Mes)), 136.4 (s, 1 C, *C*_quart_ (Mes)), 131.2 (s, 2 C, -*C*H (pyr)), 128.8 (s, 2 C, *C*_quart_ (pyr)), 127.9 (s, 6 C, -*C*H (Mes)), 118.8 (s, 2 C, -*C*H (pyr)), 21.3 (s, 1 C, -*Me*), 21.2 (s, 2 C, -*Me*), 21.1 (s, 4 C, -‍*Me*), 20.4 (s, 2 C, -*Me*) ppm.

**IR** (cm^–1^) *ν* = 2914 (m), 2854 (w), 1609 (w), 1532 (s), 1459 (m), 1374 (m), 1352 (m), 1330 (m), 1247 (s), 1236 (s), 1069 (w), 1044 (m), 976 (s), 863 (m), 851.20 (m), 835 (s), 782 (m), 726 (s), 693 (m), 614 (w), 570 (w), 501 (w), 463 (w), 442 (w), 426 (w), 411 (w).

**SS-UV/Vis** (BaSO_4_): *λ_max,ss_* = 488 nm.

Figure S1: ^1^H NMR spectrum of compound 1 in C_6_D_6_ at 300 K.

Figure S2: ^13^C NMR spectrum of compound 1 in C_6_D_6_ at 300 K.


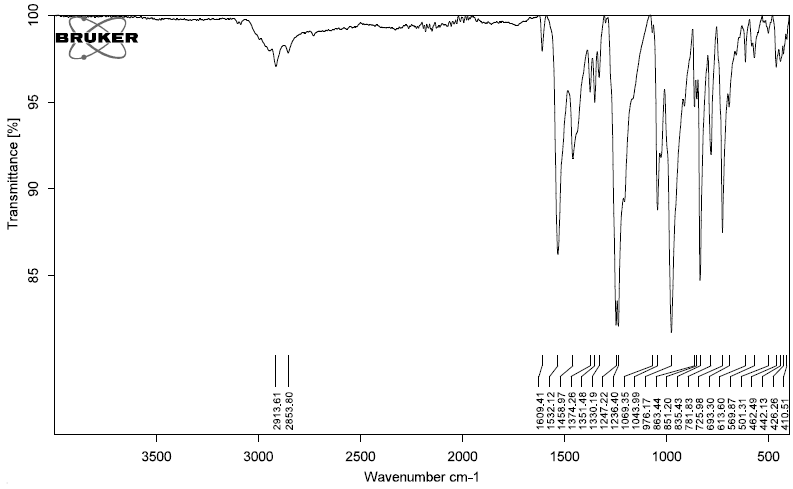


Figure S3: IR spectrum of compound 1.

Figure S4: Solid state UV/Vis spectrum of compound 1.

# 3. Synthesis and characterization of ^Mes^DPM triel dihalides

## 3.1 [(^Mes^DPM)BCl_2_] (**2**_Cl2_):

**^1^H-NMR** (400.13 MHz, C_6_D_6_) *δ* = 6.76 (s, 4 H, -C*H*(Mes)), 6.75 (s, 2 H, -C*H*(Mes)), 6.51 (d, ^3^*J*_AB_ = 4.2 Hz, 2 H, -C*H*(py)), 5.93 (d, ^3^*J*_AB_ = 4.2 Hz, 2 H, -C*H*(py)), 2.33 (s, 12 H, -*Me*), 2.17 (s, 3 H, -*Me*), 2.13 (s, 6 H, -‍*Me*), 2.12 (s, 6 H, -*Me*) ppm.

**^11^B{^1^H}-NMR** (128.38 MHz, C_6_D_6_) *δ* = 3.07 ppm.

**^13^C{^1^H}-NMR** (100.62 MHz, C_6_D_6_) *δ* = 161.41 (s, 2 C, *C*_quart_ (pyr)), 144.68 (s, 1 C, *C*_quart_ (Mes)), 138.79 (s, 1 C, *C*_quart_ (Mes)), 138.50 (s, 2 C, *C*_quart_ (Mes)), 137.39 (s, 4 C, *C*_quart_ (Mes)), 136.89 (s, 2 C, *C*_quart_ (Mes)), 135.21 (s, 2 C, *C*_quart_ (Mes)), 131.13 (s, 2 C, *C*_quart_ (pyr)), 130.63 (s, 1 C, *C*_quart_ (Mes)), 129.98 (s, 2 C, -*C*H (pyr)), 128.57 (s, 2 C, -*C*H (Mes)), 128.46 (s, 4 C, -*C*H (Mes)), 122.19 (s, 2 C, -*C*H (pyr)), 21.56 (s, 4 C, -‍*Me*), 21.25 (s, 2 C, -*Me*), 21.15 (s, 1 C, -*Me*), 19.92 (s, 2 C, -*Me*) ppm.

**IR** (cm^-1^) *ν* = 2952.18 (w), 2916.38 (w), 2856.79 (w), 1610.79 (m), 1555.29 (m), 1504.19 (m), 1459.27 (m), 1399.72 (m), 1382.26 (m), 1335.58 (m), 1253.11 (m), 1214.47 (m), 1164.39 (m), 1117.17 (s), 1086.33 (m), 1054.31 (m), 961.76 (m), 936.45 (m), 887.97 (m), 865.59 (m), 848.45 (m), 828.32 (m), 807.25 (m), 725.07 (m), 684.80 (s), 648.24 (m), 607.33 (m), 572.27 (w), 522.24 (w), 475.29 (m), 443.01 (w), 411.54 (w).

**MS (HR-ESI^+^)** (m/z (%)) calculated for [C_36_H_38_N_2_B_1_Cl_1_]^+^: 544.28166; found: 544.27796 (100.00); calculated for [C_36_H_37_N_2_B_1_Cl_1_]^+^: 543.27383; found: 543.27376 (82.32).

**SS-UV/Vis** (BaSO_4_): *λ_max,ss_* = 528 nm.

**UV/Vis** (toluene): *λ_max_* = 524 nm, *ε_max_* (524 nm) = 0.69∙10^5^ L mol^-1^cm^-1^.

**PL** (toluene, *λ_Ex_* = 405 nm): *λ_F_* = 572 nm; *φ*_F_ = 41.5%.

Figure S5: ^1^H NMR spectrum of compound 2_Cl2_ in C_6_D_6_ at 300 K.

Figure S6: ^13^C NMR spectrum of compound 2_Cl2_ in C_6_D_6_ at 300 K.


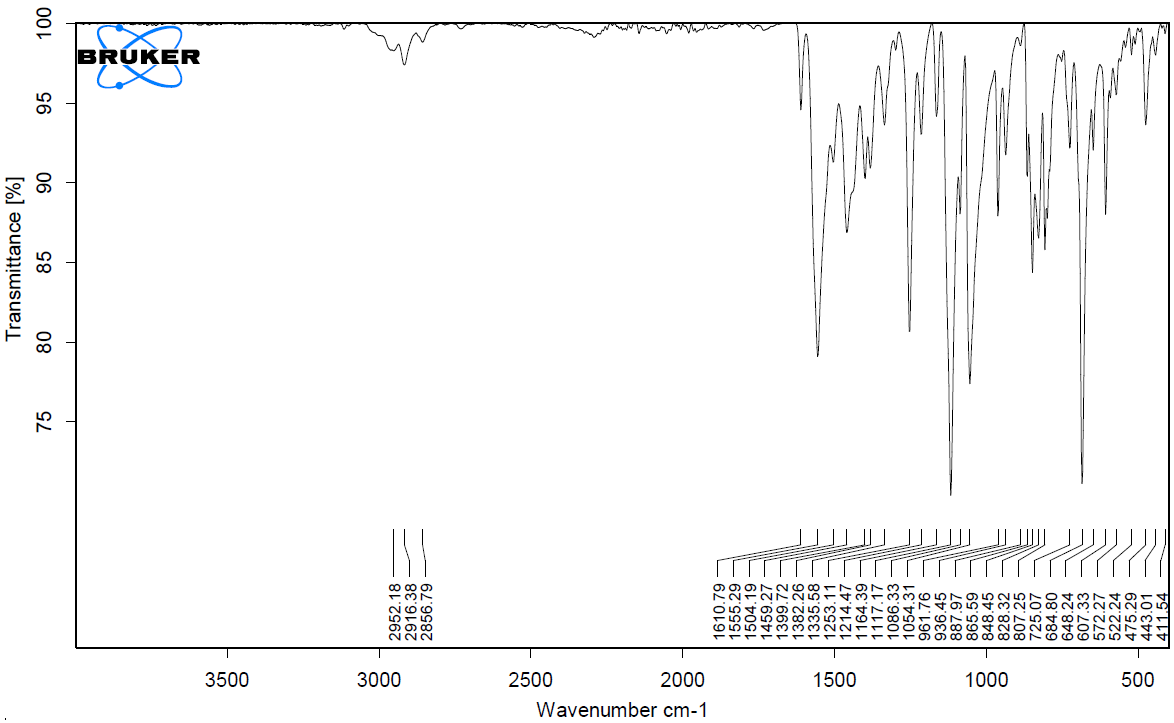


Figure S7: IR spectrum of compound 2_Cl2_.

Figure S8: Solid-state UV/VIS spectrum of compound 2_Cl2_.

Figure S9: Solution-state UV/VIS spectrum of compound 2_Cl2_ (2 µM in toluene).

Figure S10: Solution-state PL spectrum of compound 2_Cl2_ in toluene. Excitation with 405 nm continuous wave diode laser.

## 3.2 [(^Mes^DPM)BBr_2_] (**2**_Br2_):

**^1^H-NMR** (400.13 MHz, C_6_D_6_) *δ* = 6.75 (s, 2 H, -C*H*(Mes)), 6.73 (s, 4 H, -C*H*(Mes)), 6.55 (d, ^3^*J*_AB_ = 4.2 Hz, 2 H, -C*H*(py)), 5.91 (d, ^3^*J*_AB_ = 4.2 Hz, 2 H, -C*H*(py)), 2.36 (s, 12 H, -*Me*), 2.19 (s, 6 H, -*Me*), 2.17 (s, 3 H, -‍*Me*), 2.10 (s, 6 H, -*Me*) ppm.

**^11^B{^1^H}-NMR** (128.38 MHz, C_6_D_6_) *δ* = –5.40 ppm.

**^13^C{^1^H}-NMR** (100.62 MHz, C_6_D_6_) *δ* = 162.11 (s, 2 C, *C*_quart_ (pyr)), 144.84 (s, 1 C, *C*_quart_ (Mes)), 138.87 (s, 1 C, *C*_quart_ (Mes)), 138.65 (s, 2 C, *C*_quart_ (Mes)), 137.19 (s, 4 C, *C*_quart_ (Mes)), 137.08 (s, 2 C, *C*_quart_ (Mes)), 135.81 (s, 2 C, *C*_quart_ (Mes)), 131.52 (s, 2 C, *C*_quart_ (pyr)), 130.94 (s, 2 C, -*C*H (pyr)), 130.45 (s, 1 C, *C*_quart_ (Mes)), 128.50 (s, 2 C, -*C*H (Mes)), 128.21 (s, 4 C, -*C*H (Mes)), 122.72 (s, 2 C, -*C*H (pyr)), 21.74 (s, 4 C, -‍*Me*), 21.22 (s, 2 C, -*Me*), 21.15 (s, 1 C, -*Me*), 20.10 (s, 2 C, -*Me*) ppm.

**IR** (cm^-1^) *ν* = 2944.33 (w), 2915.63 (w), 2855.63 (w), 1610.17 (m), 1551.94 (s), 1500.86 (m), 1456.96 (m), 1402.34 (m), 1379.96 (m), 1339.36 (m), 1320.22 (m), 1298.75 (m), 1248.71 (m), 1213.27 (m), 1162.89 (m), 1120.08 (s), 1084.08 (m), 1049.28 (s), 961.53 (m), 935.11 (m), 865.12 (m), 845.67 (m), 831.33 (m), 798.23 (s), 734.52 (m), 716.95 (m), 682.34 (m), 644.22 (m), 616.36 (m), 571.86 (s), 518.26 (m), 472.56 (m), 416.10 (w).

**CHNS**: calculated: C = 64.70%, H = 5.58%, N = 4.19%; found: C = 65.28%, H = 5.79%, N = 4.60%.

**MS (HR-ESI^+^)** (m/z (%)) calculated for [C_36_H_37_N_2_B_1_^79^Br_1_]^+^: 587.22332; found: 587.22357 (23.13); calculated for [C_36_H_38_N_2_B_1_^81^Br_1_]^+^: 589.22127; found: 589.22592 (37.39); calculated for [C_36_H_38_N_2_B_1_Br_1_]^+^: 588.23114; found: 588.22750 (35.54).

**SS-UV/Vis** (BaSO_4_): *λ_max,ss_* = 532 nm.

**UV/Vis** (toluene): *λ_max_* = 529 nm, *ε_max_* (529 nm) = 0.48∙10^5^ L mol^-1^cm^-1^.

**PL** (toluene, *λ_Ex_* = 405 nm): *λ_F_* = 590 nm, 649 nm; *φ*_F_ = 0.97%.

Figure S11: ^1^H NMR spectrum of compound 2_Br2_ in C_6_D_6_ at 300 K.

Figure S12: ^13^C NMR spectrum of compound 2_Br2_ in C_6_D_6_ at 300 K.


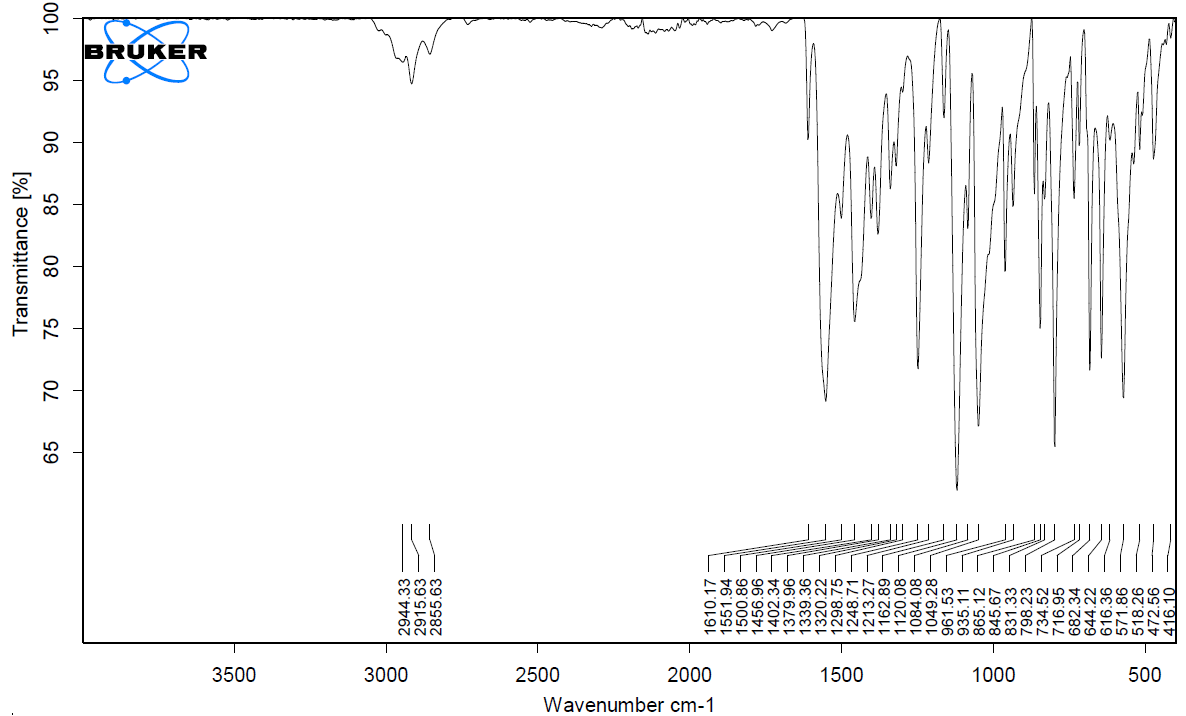


Figure S13: IR spectrum of compound 2_Br2_.

Figure S14: Solid-state UV/VIS spectrum of compound 2_Br2_.

Figure S15: Solution-state UV/VIS spectrum of compound 2_Br2_ (2 µM in toluene).

Figure S16: Solution-state PL spectrum of compound 2_Br2_ in toluene (left: 1 mM; right: 20 µM). Excitation with 405 nm continuous wave diode laser.

## 3.3 [(^Mes^DPM)AlCl_2_] (**3**_Cl2_):

**^1^H-NMR** (300.19 MHz, C_6_D_6_) *δ* = 6.76 (s, 4 H, -C*H*(Mes)), 6.74 (s, 2 H, -C*H*(Mes)), 6.59 (d, ^3^*J*_AB_ = 4.0 Hz, 2 H, -C*H*(py)), 5.98 (d, ^3^*J*_AB_ = 4.0 Hz, 2 H, -C*H*(py)), 2.31 (s, 12 H, -*Me*), 2.18 (s, 3 H, -*Me*), 2.08 (s, 6 H, -‍*Me*), 2.04 (s, 6 H, -*Me*) ppm.

**^13^C{^1^H}-NMR** (75.48 MHz, C_6_D_6_) *δ* = 164.51 (s, 2 C, *C*_quart_ (pyr)), 146.55 (s, 1 C, *C*_quart_ (Mes)), 139.40 (s, 2 C, *C*_quart_ (Mes)), 139.28 (s, 2 C, *C*_quart_ (Mes)), 138.39 (s, 1 C, *C*_quart_ (Mes)), 137.92 (s, 4 C, *C*_quart_ (Mes)), 136.83 (s, 2 C, *C*_quart_ (Mes)), 134.33 (s, 2 C, -*C*H (pyr)), 133.38 (s, 1 C, *C*_quart_ (Mes)), 129.85 (s, 2 C, *C*_quart_ (pyr)), 127.92 (s, 6 C, -*C*H (Mes)), 121.43 (s, 2 C, -*C*H (pyr)), 21.21 (s, 2 C, -*Me*), 21.15 (s, 1 C, -*Me*), 20.97 (s, 4 C, -*Me*), 19.76 (s, 2 C, -*Me*) ppm.

**^27^Al-NMR** (78.24 MHz, C_6_D_6_) *δ* = 71.16 (s, 1 Al, -*Al*Cl_2_) ppm.

**IR** (cm^-1^) *ν* = 2952.37 (w), 2917.73 (w), 2856.52 (w), 1610.74 (m), 1549.47 (s), 1519.07 (m), 1457.51 (m), 1380.78 (m), 1325.06 (m), 1240.59 (s), 1210.18 (m), 1164.70 (w), 1084.30 (m), 1070.20 (m), 1052.75 (s), 1023.79 (s), 955.96 (m), 919.60 (w), 865.35 (m), 843.16 (s), 800.51 (m), 738.00 (m), 724.92 (m), 620.70 (m), 583.52 (w), 571.26 (w), 558.47 (w), 517.27 (s), 419.20 (w).

**MS (HR-CI^+^)** (m/z (%)) calculated for [C_36_H_37_N_2_Al_1_Cl_2_]^+^: 594.21492; found: 594.21786 (100.00).

**SS-UV/Vis** (BaSO_4_): *λ_max,ss_* = 519 nm.

**UV/Vis** (toluene): *λ_max_* = 510 nm, *ε_max_* (510 nm) = 1.20∙10^5^ L mol^-1^cm^-1^.

**Excitation/Emission** (toluene): *λ_Ex_* ‍= 340 nm; *λ_Em_* = 525 nm.

**PL** (toluene, *λ_Ex_* = 405 nm): *λ_F_* = 594 nm, 657 nm; *φ*_F_ = 0.6%.

Figure S17: ^1^H NMR spectrum of compound 3_Cl2_ in C_6_D_6_ at 300 K.

Figure S18: ^13^C NMR spectrum of compound 3_Cl2_ in C_6_D_6_ at 300 K.

Figure S19: ^27^Al NMR spectrum of compound 3_Cl2_ in C_6_D_6_ at 300 K.


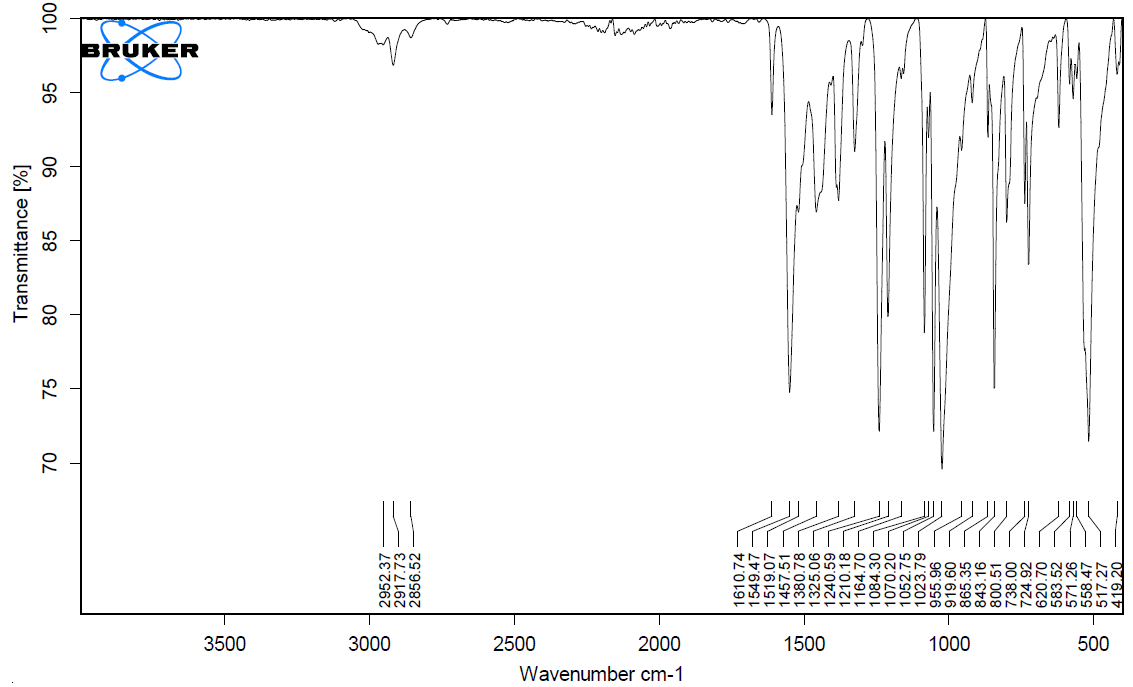


Figure S20: IR spectrum of compound 3_Cl2_.

Figure S21: Solid-state UV/VIS spectrum of compound 3_Cl2_.

Figure S22: Solution-state UV/VIS spectrum of compound 3_Cl2_ (2 µM in toluene).

Figure S23: Solution-state PL spectrum of compound 3_Cl2_ in toluene. Excitation with 405 nm continuous wave diode laser.

## 3.4 [(^Mes^DPM)AlBr_2_] (**3**_Br2_):

**^1^H-NMR** (300.19 MHz, C_6_D_6_) *δ* = 6.76 (s, 4 H, -C*H*(Mes)), 6.74 (s, 2 H, -C*H*(Mes)), 6.59 (d, ^3^*J*_AB_ = 4.0 Hz, 2 H, -C*H*(py)), 5.97 (d, ^3^*J*_AB_ = 4.0 Hz, 2 H, -C*H*(py)), 2.33 (s, 12 H, -*Me*), 2.18 (s, 3 H, -*Me*), 2.10 (s, 6 H, -‍*Me*), 2.02 (s, 6 H, -*Me*) ppm.

**^13^C{^1^H}-NMR** (75.48 MHz, C_6_D_6_) *δ* = 164.66 (s, 2 C, *C*_quart_ (pyr)), 146.57 (s, 1 C, *C*_quart_ (Mes)), 139.47 (s, 2 C, *C*_quart_ (Mes)), 139.11 (s, 2 C, *C*_quart_ (Mes)), 138.43 (s, 1 C, *C*_quart_ (Mes)),137.80 (s, 4 C, *C*_quart_ (Mes)), 136.85 (s, 2 C, *C*_quart_ (Mes)), 134.46 (s, 2 C, -*C*H (pyr)), 133.40 (s, 1 C, *C*_quart_ (Mes)), 129.90 (s, 2 C, *C*_quart_ (pyr)), 128.55 (s, 6 C, -*C*H (Mes)), 121.76 (s, 2 C, -*C*H (pyr)), 21.44 (s, 4 C, -*Me*), 21.38 (s, 1 C, -*Me*), 21.15 (s, 2 C, -*Me*), 19.79 (s, 2 C, -*Me*) ppm.

**^27^Al-NMR** (78.24 MHz, C_6_D_6_) *δ* = 70.66 (s, 1 Al, -*Al*Br_2_) ppm.

**IR** (cm^-1^) *ν* = 2973.79 (w), 2917.22 (w), 2855.55 (w), 1610.28 (m), 1552.61 (s), 1521.79 (m), 1457.39 (m), 1379.65 (m), 1324.15 (m), 1239.60 (s), 1206.67 (s), 1154.25 (m), 1084.07 (m), 1054.03 (s), 1021.87 (s), 954.90 (m), 919.70 (m), 896.01 (w), 864.38 (m), 854.23 (m), 842.58 (s), 798.40 (m), 737.07 (m), 725.36 (m), 663.32 (w), 621.52 (m), 585.23 (w), 572.68 (w), 559.14 (w), 493.46 (s), 437.80 (s).

**MS (HR-CI^+^)** (m/z (%)) calculated for [C_36_H_37_N_2_Al_1_Br_2_]^+^: 682.11389; found: 682.11674 (27.19), calculated for [C_36_H_37_N_2_Al_1_N_2_]^+^: 603.19555; found: 603.19702 (93.56).

**SS-UV/Vis** (BaSO_4_): *λ_max,ss_* = 512 nm.

**UV/Vis** (toluene): *λ_max_* = 511 nm, *ε_max_* (511 nm) = 0.89∙10^5^ L mol^-1^cm^-1^.

**Excitation/Emission** (toluene): *λ_Ex_* ‍= 343 nm; *λ_Em_* = 531 nm.

**PL** (toluene, *λ_Ex_* = 405 nm): *λ_F_* = 650 nm; *φ*_F_ = 0.3%.

Figure S24: ^1^H NMR spectrum of compound 3_Br2_ in C_6_D_6_ at 300 K.

Figure S25: ^13^C NMR spectrum of compound 3_Br2_ in C_6_D_6_ at 300 K.

Figure S26: ^27^Al NMR spectrum of compound 3_Br2_ in C_6_D_6_ at 300 K.


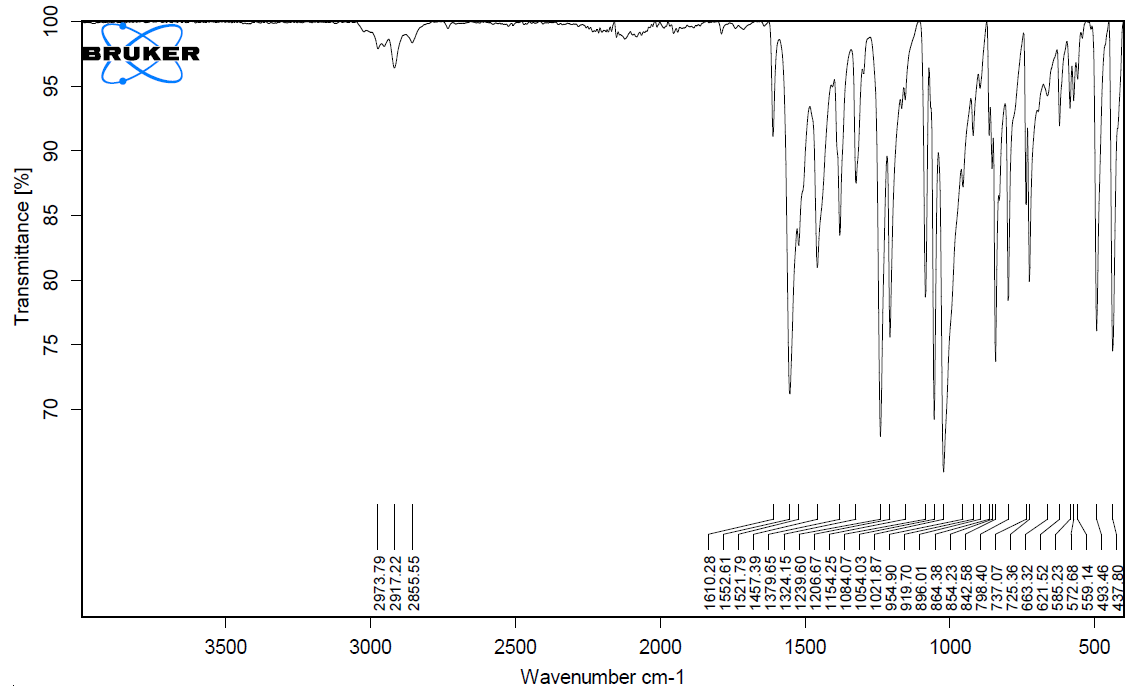


Figure S27: IR spectrum of compound 3_Br2_.

Figure S28: Solid-state UV/VIS spectrum of compound 3_Br2_.

Figure S29: Solution-state UV/VIS spectrum of compound 3_Br2_ (2 µM in toluene).

Figure S30: Solution-state PL spectrum of compound 3_Br2_ in toluene. Excitation with 405 nm continuous wave diode laser.

## 3.5 [(^Mes^DPM)AlI_2_] (**3_I2_**):

**^1^H-NMR** (300.19 MHz, C_6_D_6_) *δ* = 6.76 (s, 4 H, -C*H*(Mes)), 6.74 (s, 2 H, -C*H*(Mes)), 6.61 (d, ^3^*J*_AB_ = 4.0 Hz, 2 H, -C*H*(py)), 5.97 (d, ^3^*J*_AB_ = 4.1 Hz, 2 H, -C*H*(py)), 2.34 (s, 12 H, -*Me*), 2.19 (s, 3 H, -*Me*), 2.16 (s, 6 H, -‍*Me*), 2.04 (s, 6 H, -*Me*) ppm.

**^13^C{^1^H}-NMR** (75.48 MHz, C_6_D_6_) *δ* = 164.74 (s, 2 C, *C*_quart_ (pyr)), 146.68 (s, 1 C, *C*_quart_ (Mes)), 139.59 (s, 2 C, *C*_quart_ (Mes)), 138.91 (s, 2 C, *C*_quart_ (Mes)), 138.49 (s, 1 C, *C*_quart_ (Mes)), 137.63 (s, 4 C, *C*_quart_ (Mes)), 136.96 (s, 2 C, *C*_quart_ (Mes)), 134.91 (s, 2 C, -*C*H (pyr)), 133.37 (s, 1 C, *C*_quart_ (Mes)), 130.07 (s, 2 C, *C*_quart_ (pyr)), 128.90 (s, 6 C, -*C*H (Mes)), 122.15 (s, 2 C, -*C*H (pyr)), 22.05 (s, 4 C, -*Me*), 21.26 (s, 2 C, -*Me*), 21.17 (s, 1 C, -*Me*), 20.08 (s, 2 C, -*Me*) ppm.

**^27^Al-NMR** (78.24 MHz, C_6_D_6_) *δ* = 69.05 (s, 1 Al, -*Al*I_2_) ppm.

**IR** (cm^-1^) *ν* = 2971.10 (w), 2914.79 (w), 2854.32 (w), 1609.88 (m), 1553.44 (s), 1521.28 (m), 1455.38 (m), 1378.59 (m), 1319.97 (m), 1236.94 (s), 1202.85 (s), 1083.15 (m), 1054.30 (s), 1019.16 (s), 954.06 (m), 917.59 (m), 863.64 (m), 841.79 (s), 798.59 (m), 736.12 (m), 725.16 (m), 621.08 (m), 584.06 (m), 572.36 (w), 558.22 (w), 480.44 (s), 416.35 (w).

**MS (HR-CI^+^)** (m/z (%)) calculated for [C_36_H_37_N_2_Al_1_I_2_]^+^: 778.08615; found: 778.08389 (31.94).

**CHNS**: calculated: C = 55.54%, H = 4.79%, N = 3.60%; found: C = 55.97%, H = 5.00%, N = 3.65%.

**SS-UV/Vis** (BaSO_4_): *λ_max,ss_* = 509 nm.

**UV/Vis** (toluene): *λ_max_* = 510 nm, *ε_max_* (510 nm) = 1.25∙10^5^ L mol^-1^cm^-1^.

**Excitation/Emission** (toluene): *λ_Ex_* ‍= 345 nm; *λ_Em_* = 536 nm.

**PL** (toluene, *λ_Ex_* = 405 nm): *λ_F_* = 644 nm; *φ*_F_ = 0.02%.

Figure S31: ^1^H NMR spectrum of compound 3_I2_ in C_6_D_6_ at 300 K.

Figure S32: ^13^C NMR spectrum of compound 3_I2_ in C_6_D_6_ at 300 K.

Figure S33: ^27^Al NMR spectrum of compound 3_I2_ in C_6_D_6_ at 300 K.


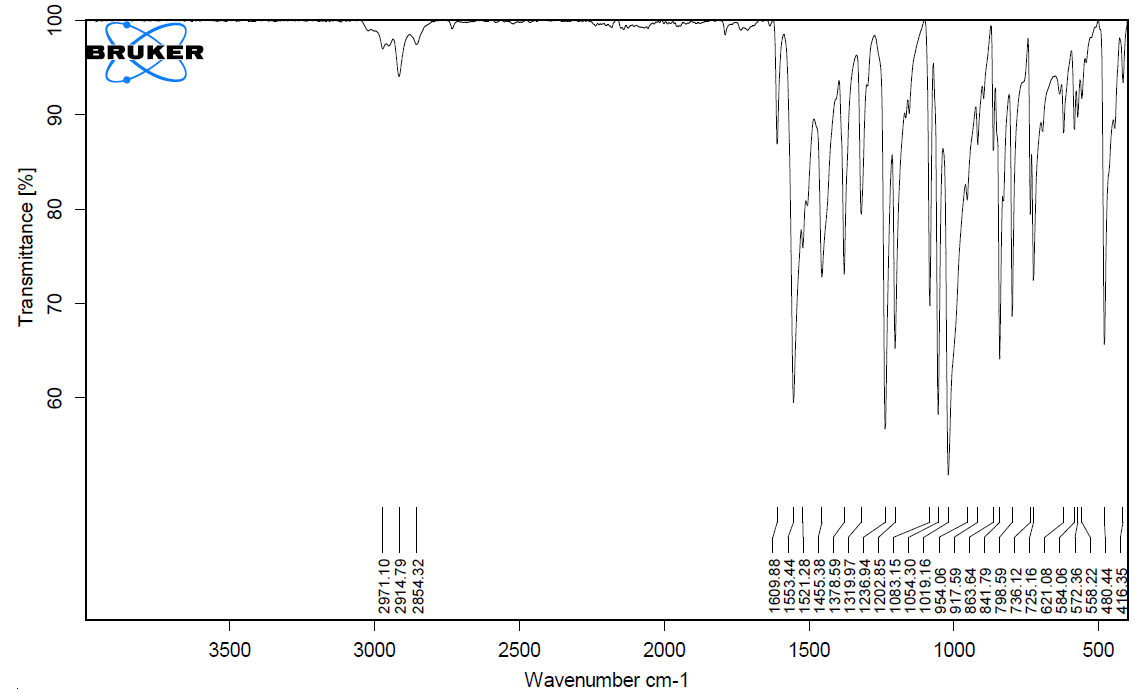


Figure 34: IR spectrum of compound 3_I2_.

Figure S35: Solid-state UV/VIS spectrum of compound 3_I2_.

Figure S36: Solution-state UV/VIS spectrum of compound 3_I2_ (2 µM in toluene).

Figure S37: Solution-state PL spectrum of compound 3_I2_ in toluene. Excitation with 405 nm continuous wave diode laser.

## 3.6 [(^Mes^DPM)GaCl_2_] (**4**_Cl2_):

**^1^H-NMR** (300.19 MHz, C_6_D_6_) *δ* = 6.76 (s, 4 H, -C*H*(Mes)), 6.74 (s, 2 H, -C*H*(Mes)), 6.60 (d, ^3^*J*_AB_ = 4.1 Hz, 2 H, -C*H*(py)), 5.99 (d, ^3^*J*_AB_ = 4.1 Hz, 2 H, -C*H*(py)), 2.31 (s, 12 H, -*Me*), 2.18 (s, 3 H, -*Me*), 2.08 (s, 6 H, -‍*Me*), 2.03 (s, 6 H, -*Me*) ppm.

**^13^C{^1^H}-NMR** (75.48 MHz, C_6_D_6_) *δ* = 163.77 (s, 2 C, *C*_quart_ (pyr)), 145.81 (s, 1 C, *C*_quart_ (Mes)), 139.46 (s, 2 C, *C*_quart_ (Mes)), 138.40 (s, 1 C, *C*_quart_ (Mes)), 138.34 (s, 2 C, *C*_quart_ (Mes)), 137.90 (s, 4 C, *C*_quart_ (Mes)), 136.84 (s, 2 C, *C*_quart_ (Mes)), 133.94 (s, 2 C, -*C*H (pyr)), 133.54 (s, 1 C, *C*_quart_ (Mes)), 129.65 (s, 2 C, *C*_quart_ (pyr)), 128.44 (s, 6 C, -*C*H (Mes)), 120.86 (s, 2 C, -*C*H (pyr)), 21.22 (s, 2 C, -*Me*), 21.16 (s, 1 C, -*Me*), 20.96 (s, 4 C, -*Me*), 19.75 (s, 2 C, -*Me*) ppm.

**IR** (cm^-1^) *ν* = 2967.39 (w), 2917.60 (w), 2856.96 (w), 1610.40 (m), 1551.79 (s), 1460.12 (m), 1377.00 (m), 1330.89 (m), 1246.41 (s), 1215.05 (m), 1164.68 (w), 1081.39 (m), 1053.95 (s), 1021.09 (s), 917.69 (w), 864.16 (m), 841.74 (s), 792.37 (m), 734.40 (m), 725.02 (m), 620.64 (w), 582.54 (w), 571.71 (w), 463.99 (w), 428.39 (m).

**MS (HR-CI^+^)** (m/z (%)) calculated for [C_36_H_37_N_2_Ga_1_Cl_2_]^+^: 636.15896; found: 636.15833 (10.88).

**SS-UV/Vis** (BaSO_4_): *λ_max,ss_* = 517 nm.

**UV/Vis** (toluene): *λ_max_* = 508 nm, *ε_max_* (508 nm) = 1.31∙10^5^ L mol^-1^cm^-1^.

**Excitation/Emission** (toluene): *λ_Ex_* ‍= 341 nm; *λ_Em_* = 528 nm.

**PL** (toluene, *λ_Ex_* = 405 nm): *λ_F_* = 560 nm; *φ*_F_ = 19%.

Figure S38: ^1^H NMR spectrum of compound 4_Cl2_ in C_6_D_6_ at 300 K.

Figure S39: ^13^C NMR spectrum of compound 4_Cl2_ in C_6_D_6_ at 300 K.


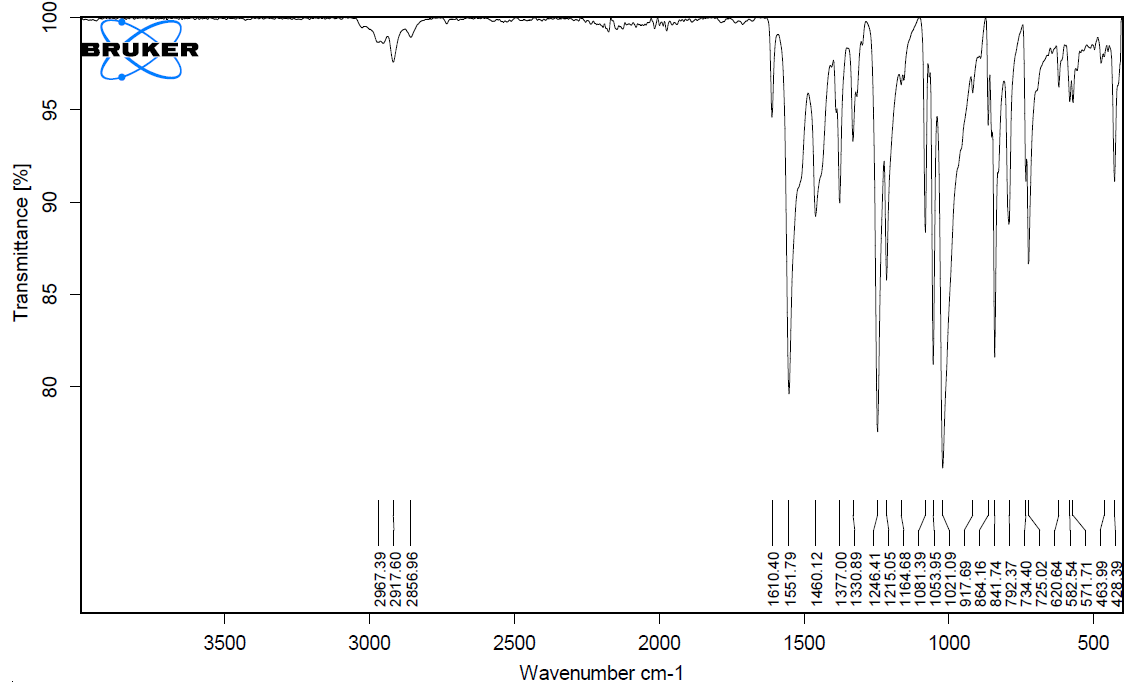


Figure S40: IR spectrum of compound 4_Cl2_.

Figure S41: Solid-state UV/VIS spectrum of compound 4_Cl2_.

Figure S42: Solution-state UV/VIS spectrum of compound 4_Cl2_ (2 µM in toluene).

Figure S43: Solution-state PL spectrum of compound 4_Cl2_ in toluene. Excitation with 405 nm continuous wave diode laser.

## 3.7 [(^Mes^DPM)GaBr_2_] (**4_Br2_**):

**^1^H-NMR** (300.19 MHz, C_6_D_6_) *δ* = 6.76 (s, 4 H, -C*H*(Mes)), 6.75 (s, 2 H, -C*H*(Mes)), 6.60 (d, ^3^*J*_AB_ = 4.1 Hz, 2 H, -C*H*(py)), 5.99 (d, ^3^*J*_AB_ = 4.1 Hz, 2 H, -C*H*(py)), 2.32 (s, 12 H, -*Me*), 2.19 (s, 3 H, -*Me*), 2.10 (s, 6 H, -‍*Me*), 2.02 (s, 6 H, -*Me*) ppm.

**^13^C{^1^H}-NMR** (75.48 MHz, C_6_D_6_) *δ* = 163.83 (s, 2 C, *C*_quart_ (pyr)), 145.81 (s, 1 C, *C*_quart_ (Mes)), 139.48 (s, 2 C, *C*_quart_ (Mes)), 138.39 (s, 1 C, *C*_quart_ (Mes)), 138.14 (s, 2 C, *C*_quart_ (Mes)), 137.81 (s, 4 C, *C*_quart_ (Mes)), 136.87 (s, 2 C, *C*_quart_ (Mes)), 133.95 (s, 2 C, -*C*H (pyr)), 133.63 (s, 1 C, *C*_quart_ (Mes)), 129.70 (s, 2 C, *C*_quart_ (pyr)), 128.56 (s, 6 C, -*C*H (Mes)), 121.10 (s, 2 C, -*C*H (pyr)), 21.42 (s, 4 C, -*Me*), 21.22 (s, 2 C, -*Me*), 21.16 (s, 1 C, -*Me*), 19.80 (s, 2 C, -*Me*) ppm.

**IR** (cm^-1^) *ν* = 2972.05 (w), 2916.47 (w), 2856.00 (w), 2122.13 (w), 1610.41 (w), 1552.41 (s), 1514.00 (m), 1459.56 (m), 1377.06 (m), 1328.67 (m), 1244.43 (s), 1213.61 (m), 1080.89 (m), 1054.10 (s), 1019.69 (s), 917.88 (w), 863.72 (m), 854.10 (m), 840.88 (s), 795.31 (m), 725.11 (m), 620.78 (w), 572.66 (w), 473.42 (w), 424.07 (m).

**MS (HR-CI^+^)** (m/z (%)) calculated for [C_36_H_37_N_2_Ga_1_Br_2_]^+^: 724.05793; found: 724.05821 (36.73).

**SS-UV/Vis** (BaSO_4_): *λ_max,ss_* = 510 nm.

**UV/Vis** (toluene): *λ_max_* = 509 nm, *ε_max_* (509 nm) = 1.00∙10^5^ L mol^-1^cm^-1^.

**Excitation/Emission** (toluene): *λ_Ex_* ‍= 344 nm; *λ_Em_* = 532 nm.

**PL** (toluene, *λ_Ex_* = 405 nm): *λ_F_* = 554 nm; *φ*_F_ = 22%.

Figure S44: ^1^H NMR spectrum of compound 4_Br2_ in C_6_D_6_ at 300 K.

Figure S45: ^13^C NMR spectrum of compound 4_Br2_ in C_6_D_6_ at 300 K.


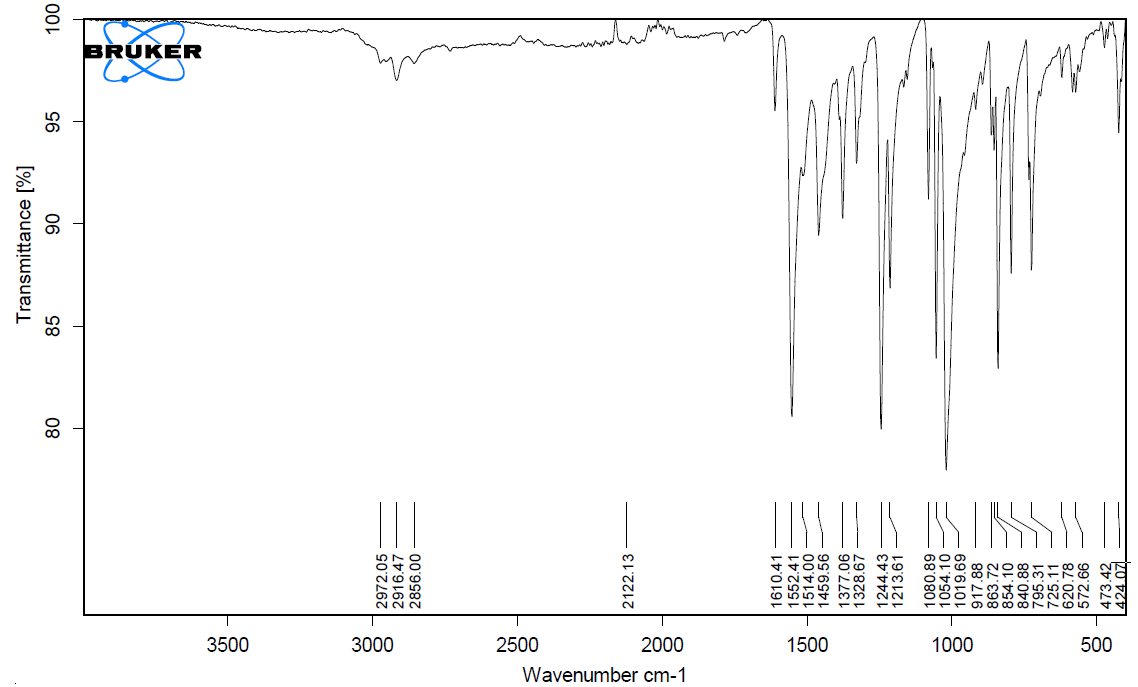


Figure S46: IR spectrum of compound 4_Br2_.

Figure S47: Solid-state UV/VIS spectrum of compound 4_Br2_.

Figure S48: Solution-state UV/VIS spectrum of compound 4_Br2_ (2 µM in toluene).

Figure S49: Solution-state PL spectrum of compound 4_Br2_ in toluene. Excitation with 405 nm continuous wave diode laser.

## 3.8 [(^Mes^DPM)GaI_2_] (**4**_I2_):

**^1^H-NMR** (300.19 MHz, C_6_D_6_) *δ* = 6.77 (s, 4 H, -C*H*(Mes)), 6.76 (s, 2 H, -C*H*(Mes)), 6.63 (d, ^3^*J*_AB_ = 4.1 Hz, 2 H, -C*H*(py)), 5.98 (d, ^3^*J*_AB_ = 4.1 Hz, 2 H, -C*H*(py)), 2.33 (s, 12 H, -*Me*), 2.19 (s, 3 H, -*Me*), 2.17 (s, 6 H, -‍*Me*), 2.05 (s, 6 H, -*Me*) ppm.

**^13^C{^1^H}-NMR** (75.48 MHz, C_6_D_6_) *δ* = 163.66 (s, 2 C, *C*_quart_ (pyr)), 145.89 (s, 1 C, *C*_quart_ (Mes)), 139.50 (s, 2 C, *C*_quart_ (Mes)), 138.38 (s, 1 C, *C*_quart_ (Mes)), 137.93 (s, 2 C, *C*_quart_ (Mes)), 137.69 (s, 4 C, *C*_quart_ (Mes)), 136.98 (s, 2 C, *C*_quart_ (Mes)), 133.98 (s, 2 C, -*C*H (pyr)), 133.72 (s, 1 C, *C*_quart_ (Mes)), 129.79 (s, 2 C, *C*_quart_ (pyr)), 128.86 (s, 6 C, -*C*H (Mes)), 121.36 (s, 2 C, -*C*H (pyr)), 22.03 (s, 4 C, -*Me*), 21.27 (s, 2 C, -*Me*), 21.17 (s, 1 C, -*Me*), 20.11 (s, 2 C, -*Me*) ppm.

**IR** (cm^-1^) *ν* = 2970.82 (w), 2914.97 (m), 2852.93 (w), 1784.60 (w), 1609.95 (m), 1552.38 (s), 1507.69 (m), 1457.56 (m), 1375.43 (m), 1325.09 (m), 1239.83 (s), 1210.13 (s), 1154.13 (m), 1079.98 (m), 1054.09 (s), 1016.57 (s, br), 916.10 (m), 862.97 (m), 851.58 (m), 839.78 (s), 794.88 (s), 724.85 (s), 620.49 (m), 582.51 (m), 572.68 (m), 558.55 (m), 496.64 (w), 472.91 (w), 421.74 (m).

**MS (HR-CI^+^)** (m/z (%)) calculated for [C_36_H_37_N_2_Ga_1_I_2_]^+^: 820.03019; found: 820.03267 (25.73).

**CHNS**: calculated: C = 52.65%, H = 4.54%, N = 3.41%; found: C = 53.68%, H = 4.620%, N = 3.33%.

**SS-UV/Vis** (BaSO_4_): *λ_max,ss_* = 507 nm.

**UV/Vis** (toluene): *λ_max_* = 512 nm, *ε_max_* (512 nm) = 1.02∙10^5^ L mol^-1^cm^-1^.

**Excitation/Emission** (toluene): *λ_Ex_* ‍= 356 nm; *λ_Em_* = 539 nm.

**PL** (toluene, *λ_Ex_* = 405 nm): *λ_F_* = 566 nm; *φ*_F_ = 14%.

Figure S50: ^1^H NMR spectrum of compound 4_I2_ in C_6_D_6_ at 300 K.

Figure S51: ^13^C NMR spectrum of compound 4_I2_ in C_6_D_6_ at 300 K.


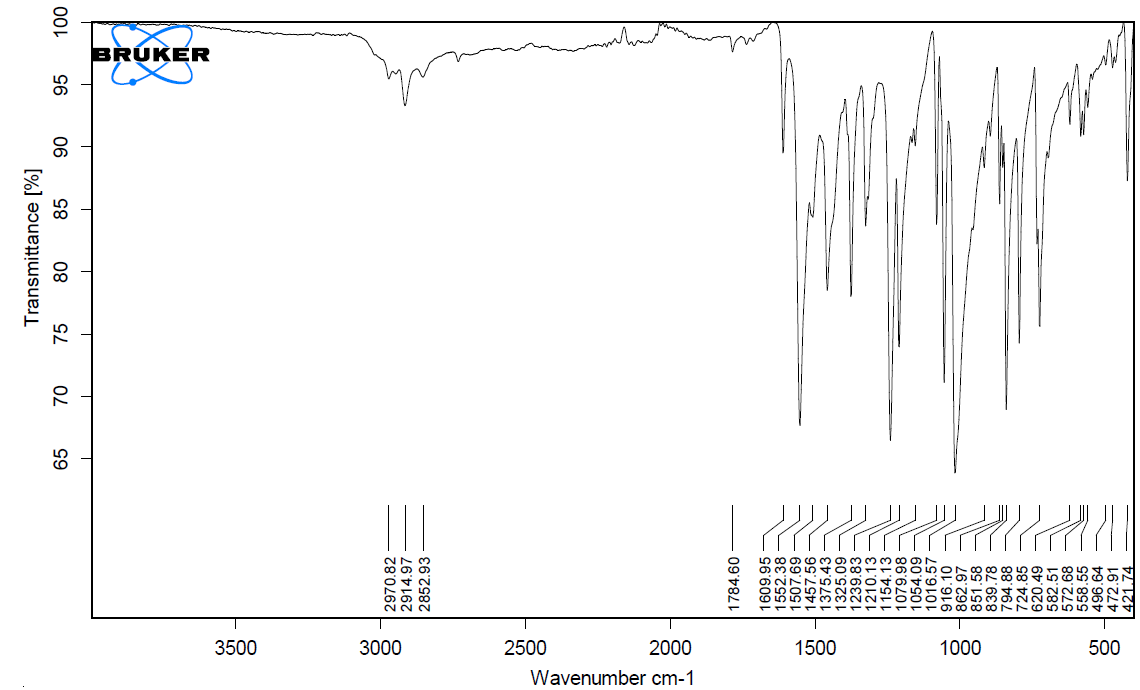


Figure S52: IR spectrum of compound 4_I2_.

Figure S53: Solid-state UV/VIS spectrum of compound 4_I2_.

Figure S54: Solution-state UV/VIS spectrum of compound 4_I2_ (2 µM in toluene).

Figure S55: Solution-state PL spectrum of compound 4_I2_ in toluene. Excitation with 405 nm continuous wave diode laser.

## 3.9 [(^Mes^DPM)InCl_2_] (**5_Cl2_**):

**^1^H-NMR** (300.19 MHz, C_6_D_6_) *δ* = 6.77 (s, 4 H, -C*H*(Mes)), 6.76 (s, 2 H, -C*H*(Mes)), 6.66 (d, ^3^*J*_AB_ = 4.1 Hz, 2 H, -C*H*(py)), 6.04 (d, ^3^*J*_AB_ = 4.1 Hz, 2 H, -C*H*(py)), 2.29 (s, 12 H, -*Me*), 2.20 (s, 3 H, -*Me*), 2.09 (s, 6 H, -‍*Me*), 1.98 (s, 6 H, -*Me*) ppm.

**^13^C{^1^H}-NMR** (75.48 MHz, C_6_D_6_) *δ* = 164.05 (s, 2 C, *C*_quart_ (pyr)), 146.21 (s, 1 C, *C*_quart_ (Mes)), 139.92 (s, 2 C, *C*_quart_ (Mes)), 139.63 (s, 2 C, *C*_quart_ (Mes)), 138.20 (s, 1 C, *C*_quart_ (Mes)), 137.97 (s, 4 C, *C*_quart_ (Mes)), 136.83 (s, 2 C, *C*_quart_ (Mes)), 135.17 (s, 2 C, -*C*H (pyr)), 134.67 (s, 1 C, *C*_quart_ (Mes)), 130.26 (s, 2 C, *C*_quart_ (pyr)), 128.86 (s, 6 C, -*C*H (Mes)), 120.02 (s, 2 C, -*C*H (pyr)), 21.17 (s, 2 C, -*Me*), 21.16 (s, 1 C, -*Me*), 20.70 (s, 4 C, -*Me*), 19.74 (s, 2 C, -*Me*) ppm.

**IR** (cm^-1^) *ν* = 2972.39 (w), 2919.33 (w), 2856.13 (w), 1610.70 (m), 1544.59 (s), 1460.27 (m), 1373.15 (m), 1328.94 (m), 1311.75 (m), 1239.58 (s), 1152.88 (m), 1079.53 (m), 1052.18 (s), 1011.20 (s), 915.07 (w), 892.89 (w), 863.54 (m), 854.17 (m), 839.13 (s), 792.52 (s), 724.94 (s), 618.76 (w), 571.94 (w), 559.28 (w), 524.23 (w), 468.48 (w), 411.40 (w).

**MS (HR-CI^+^)** (m/z (%)) calculated for [C_36_H_37_N_2_In_1_Cl_2_]^+^: 682.13726; found: 682.13396 (41.62), calculated for [C_36_H_37_N_2_In_1_Cl_1_]^+^: 647.16840; found: 647.16727 (51.17).

**CHNS**: calculated: C = 63.27%, H = 5.46%, N = 4.10%; found: C = 63.30%, H = 5.678%, N = 4.06%.

**SS-UV/Vis** (BaSO_4_): *λ_max,ss_* = 518 nm.

**UV/Vis** (toluene): *λ_max_* = 505 nm, *ε_max_* (505 nm) = 1.47∙10^5^ L mol^-1^cm^-1^.

**Excitation/Emission** (toluene): *λ_Ex_* = 336 nm; *λ_Em_* = 530 nm.

**PL** (toluene, *λ_Ex_* = 405 nm): *λ_F_* = 613 nm, 655 nm; *φ*_F_ = 0.4%.

Figure S56: ^1^H NMR spectrum of compound 5_Cl2_ in C_6_D_6_ at 300 K.

Figure S57: ^13^C NMR spectrum of compound 5_Cl2_ in C_6_D_6_ at 300 K.


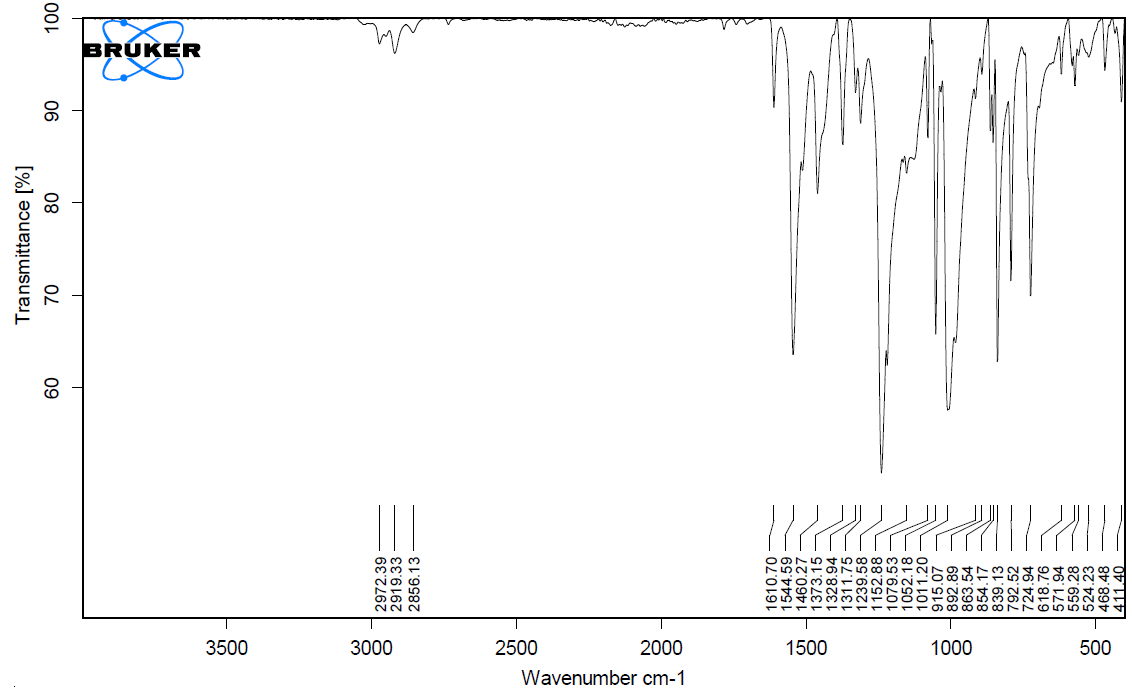


Figure S58: IR spectrum of compound 5_Cl2_.

Figure S59: Solid-state UV/VIS spectrum of compound 5_Cl2_.

Figure S60: Solution-state UV/VIS spectrum of compound 5_Cl2_ (2 µM in toluene).

Figure S61: Solution-state PL spectrum of compound 5_Cl2_ in toluene. Excitation with 405 nm continuous wave diode laser.

## 3.10 [(^Mes^DPM)InBr_2_] (**5**_Br2_):

**^1^H-NMR** (300.19 MHz, C_6_D_6_) *δ* = 6.79 (s, 4 H, -C*H*(Mes)), 6.75 (s, 2 H, -C*H*(Mes)), 6.66 (d, ^3^*J*_AB_ = 4.1 Hz, 2 H, -C*H*(py)), 6.04 (d, ^3^*J*_AB_ = 4.1 Hz, 2 H, -C*H*(py)), 2.30 (s, 12 H, -*Me*), 2.20 (s, 3 H, -*Me*), 2.10 (s, 6 H, -‍*Me*), 1.99 (s, 6 H, -*Me*) ppm.

**^13^C{^1^H}-NMR** (75.48 MHz, C_6_D_6_) *δ* = 164.04 (s, 2 C, *C*_quart_ (pyr)), 146.21 (s, 1 C, *C*_quart_ (Mes)), 139.86 (s, 2 C, *C*_quart_ (Mes)), 139.41(s, 2 C, *C*_quart_ (Mes)), 138.17 (s, 1 C, *C*_quart_ (Mes)), 137.92 (s, 4 C, *C*_quart_ (Mes)), 136.84 (s, 2 C, *C*_quart_ (Mes)), 135.05 (s, 2 C, -*C*H (pyr)), 134.82 (s, 1 C, *C*_quart_ (Mes)), 130.34 (s, 2 C, *C*_quart_ (pyr)), 128.91 (s, 6 C, -*C*H (Mes)), 120.19 (s, 2 C, -*C*H (pyr)), 21.20 (s, 2 C, -*Me*), 21.17 (s, 1 C, -*Me*), 21.17 (s, 4 C, -*Me*), 19.76 (s, 2 C, -*Me*) ppm.

**IR** (cm^-1^) *ν* = 2970.46 (w), 2918.00 (w), 2855.55 (w), 1610.57 (m), 1544.65 (s), 1511.56 (m), 1459.31 (m), 1372.22 (m), 1328.35 (m), 1312.50 (m), 1239.91 (s), 1217.61 (s), 1153.90 (w), 1079.10 (m), 1052.37 (s), 1010.96 (s), 863.26 (m), 852.87 (m), 838.94 (s), 792.58 (m), 725.25 (m), 618.50 (w), 571.97 (w), 468.17 (w), 411.21 (w).

**MS (HR-CI^+^)** (m/z (%)) calculated for [C_36_H_37_N_2_In_1_Br_2_]^+^: 770.03623; found: 770.03887 (50.11), calculated for [C_36_H_37_N_2_In_1_Br_1_]^+^: 691.11789; found: 691.12023 (89.89).

**CHNS**: calculated: C = 55.99%, H = 4.83%, N = 3.63%; found: C = 55.22%, H = 5.023%, N = 3.62%.

**SS-UV/Vis** (BaSO_4_): *λ_max,ss_* = 517 nm.

**UV/Vis** (toluene): *λ_max_* = 505 nm, *ε_max_* (505 nm) = 1.02∙10^5^ L mol^-1^cm^-1^.

**Excitation/Emission** (toluene): *λ_Ex_* ‍= 340 nm; *λ_Em_* = 536 nm.

**PL** (toluene, *λ_Ex_* = 405 nm): *λ_F_* = 623 nm; *φ*_F_ = 0.3%.

Figure S62: ^1^H NMR spectrum of compound 5_Br2_ in C_6_D_6_ at 300 K.

Figure S63: ^13^C NMR spectrum of compound 5_Br2_ in C_6_D_6_ at 300 K.


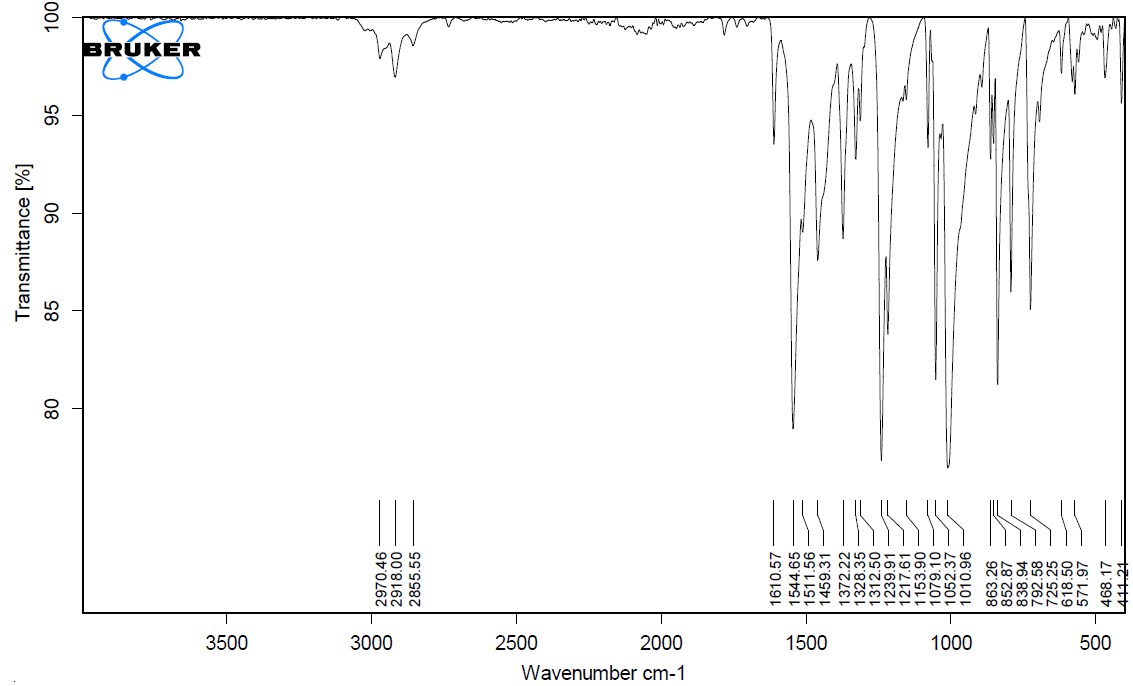


Figure S64: IR spectrum of compound 5_Br2_.

Figure S65: Solid-state UV/VIS spectrum of compound 5_Br2_.

Figure S66: Solution-state UV/VIS spectrum of compound 5_Br2_ (2 µM in toluene).

Figure S67: Solution-state PL spectrum of compound 5_Br2_ in toluene. Excitation with 405 nm continuous wave diode laser.

## 3.11 [(^Mes^DPM)InI_2_] (**5**_I2_):

**^1^H-NMR** (300.19 MHz, C_6_D_6_) *δ* = 6.79 (s, 4 H, -C*H*(Mes)), 6.76 (s, 2 H, -C*H*(Mes)), 6.67 (d, ^3^*J*_AB_ = 4.1 Hz, 2 H, -C*H*(py)), 6.03 (d, ^3^*J*_AB_ = 4.2 Hz, 2 H, -C*H*(py)), 2.31 (s, 12 H, -*Me*), 2.20 (s, 3 H, -*Me*), 2.14 (s, 6 H, -‍*Me*), 2.03 (s, 6 H, -*Me*) ppm.

**^13^C{^1^H}-NMR** (125.79 MHz, C_6_D_6_) *δ* = 163.86 (s, 2 C, *C*_quart_ (pyr)), 146.23 (s, 1 C, *C*_quart_ (Mes)), 139.73 (s, 2 C, *C*_quart_ (Mes)), 139.05(s, 2 C, *C*_quart_ (Mes)), 138.09 (s, 1 C, *C*_quart_ (Mes)), 137.72 (s, 4 C, *C*_quart_ (Mes)), 136.84 (s, 2 C, *C*_quart_ (Mes)), 134.99 (s, 1 C, *C*_quart_ (Mes)), 134.84 (s, 2 C, -*C*H (pyr)), 130.49 (s, 2 C, *C*_quart_ (pyr)), 129.11 (s, 6 C, -*C*H (Mes)), 120.38 (s, 2 C, -*C*H (pyr)), 21.99 (s, 4 C, -*Me*), 21.27 (s, 2 C, -*Me*), 21.18 (s, 1 C, -*Me*), 19.92 (s, 2 C, -*Me*) ppm.

**IR** (cm^-1^) *ν* = 2968.24 (w), 2915.77 (w), 2852.77 (w), 1609.72 (m), 1544.58 (s), 1457.33 (m), 1370.51 (m), 1326.35 (m), 1311.18 (m), 1237.68 (s), 1215.62 (m), 1078.50 (m), 1052.03 (s), 1005.07 (s), 862.67 (m), 850.42 (w), 838.36 (s), 791.89 (m), 725.53 (m), 693.77 (m), 618.08 (w), 571.55 (w), 464.22 (w), 409.45 (w).

**MS (HR-CI^+^)** (m/z (%)) calculated for [C_36_H_37_N_2_In_1_I_2_]^+^: 866.00849; found: 866.00813 (53.96).

**SS-UV/Vis** (BaSO_4_): *λ_max,ss_* = 513 nm.

**UV/Vis** (toluene): *λ_max_* = 508 nm, *ε_max_* (508 nm) = 0.58∙10^5^ L mol^-1^cm^-1^.

**PL** (toluene, *λ_Ex_* = 405 nm): *λ_F_* = 622 nm; *φ*_F_ = 0.2%.

Figure S68: ^1^H NMR spectrum of compound 5_I2_ in C_6_D_6_ at 300 K.

Figure S69: ^13^C NMR spectrum of compound 5_I2_ in C_6_D_6_ at 300 K.


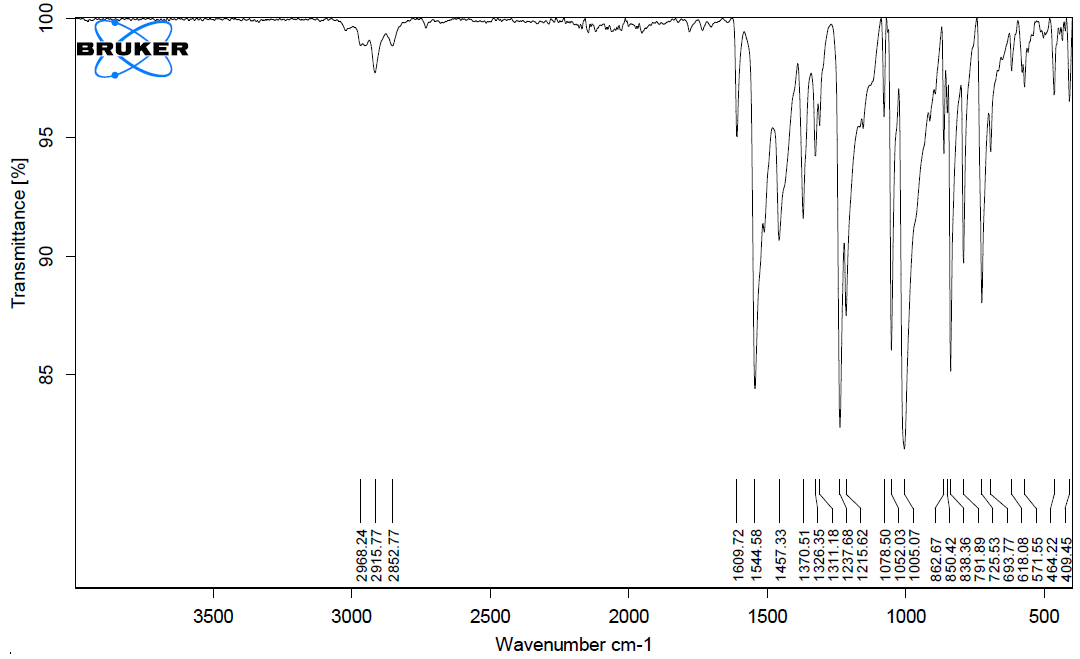


Figure S70: IR spectrum of compound 5_I2_.

Figure S71: Solid-state UV/VIS spectrum of compound 5_I2_.

Figure S72: Solution-state UV/VIS spectrum of compound 5_I2_ (2 µM in toluene).

Figure S73: Solution-state PL spectrum of compound 5_I2_ in toluene. Excitation with 405 nm continuous wave diode laser.

# 4. Synthesis and characterization of [(^Mes^DPM)BMe_2_] (2_Me2_):

**^1^H-NMR** (400.13 MHz, C_6_D_6_) *δ* = 6.82 (s, 2 H, -C*H*(Mes)), 6.74 (s, 4 H, -C*H*(Mes)), 6.69 (d, ^3^*J*_AB_ = 4.2 Hz, 2 H, -C*H*(py)), 6.04 (d, ^3^*J*_AB_ = 4.2 Hz, 2 H, -C*H*(py)), 2.24 (s, 6 H, -‍*Me*), 2.20 (s, 15 H, -*Me*), 2.11 (s, 6 H, -‍*Me*), 0.15 (s, 6 H, -B*Me*_2_) ppm.

**^11^B{^1^H}-NMR** (128.38 MHz, C_6_D_6_) *δ* = –20.67 ppm.

**^13^C{^1^H}-NMR** (100.62 MHz, C_6_D_6_) *δ* = 157.29 (s, 2 C, *C*_quart_ (pyr)), 143.85 (s, 1 C, *C*_quart_ (Mes)), 138.05 (s, 1 C, *C*_quart_ (Mes)), 137.96 (s, 2 C, *C*_quart_ (Mes)), 137.10 (s, 4 C, *C*_quart_ (Mes)), 136.82 (s, 2 C, *C*_quart_ (Mes)), 135.57 (s, 2 C, *C*_quart_ (Mes)), 133.13 (s, 2 C, *C*_quart_ (pyr)), 132.54 (s, 1 C, *C*_quart_ (Mes)), 128.41 (s, 2 C, -*C*H (Mes)), 128.37 (s, 4 C, -*C*H (Mes)), 127.35 (s, 2 C, -*C*H (pyr)), 120.20 (s, 2 C, -*C*H (pyr)), 21.19 (s, 3 C, -‍*Me*), 21.16 (s, 4 C, -*Me*), 20.01 (s, 1 C, -*Me*), 14.85 (s, 2 C, -B*Me*) ppm.

**IR** (cm^-1^) *ν* = 2948.97 (m), 2917.11 (m), 2856.42 (w), 1611.27 (m), 1557.68 (s), 1456.97 (m), 1440.42 (m), 1401.03 (m), 1377.72 (m), 1353.59 (w), 1299.12 (w), 1267.41 (s), 1218.42 (m), 1163.95 (w), 1140.65 (w), 1090.99 (s), 1062.44 (m), 1042.57 (s), 967.59 (m), 948.76 (m), 867.54 (w), 841.84 (s), 829.53 (m), 788.24 (m), 732.56 (m), 724.13 (m), 627.11 (w), 597.16 (w), 571.29 (w), 544.94 (w), 466.91 (w), 424.85 (w).

**MS (HR-CI^+^)** (m/z (%)) calculated for [C_38_H_44_N_2_B_1_]^+^: 539.35975; found: 539.35933 (36.53), calculated for [C_38_H_43_N_2_B_1_]^+^: 538.35193; found: 538.35403 (25.66).

**SS-UV/Vis** (BaSO_4_): *λ_max,ss_* = 510 nm.

**UV/Vis** (toluene): *λ_max_* = 515 nm, *ε_max_* (515 nm) = 0.13∙10^5^ L mol^-1^cm^-1^.

**PL** (toluene, *λ_Ex_* = 405 nm): *λ_F_* = 538 nm, 649 nm; *φ*_F_ = 1.77%.

Figure S74: ^1^H NMR spectrum of compound 2_Me2_ in C_6_D_6_ at 300 K.

Figure 75: ^11^B NMR spectrum of compound 2_Me2_ in C_6_D_6_ at 300 K.

Figure S76: ^13^C NMR spectrum of compound 2_Me2_ in C_6_D_6_ at 300 K.


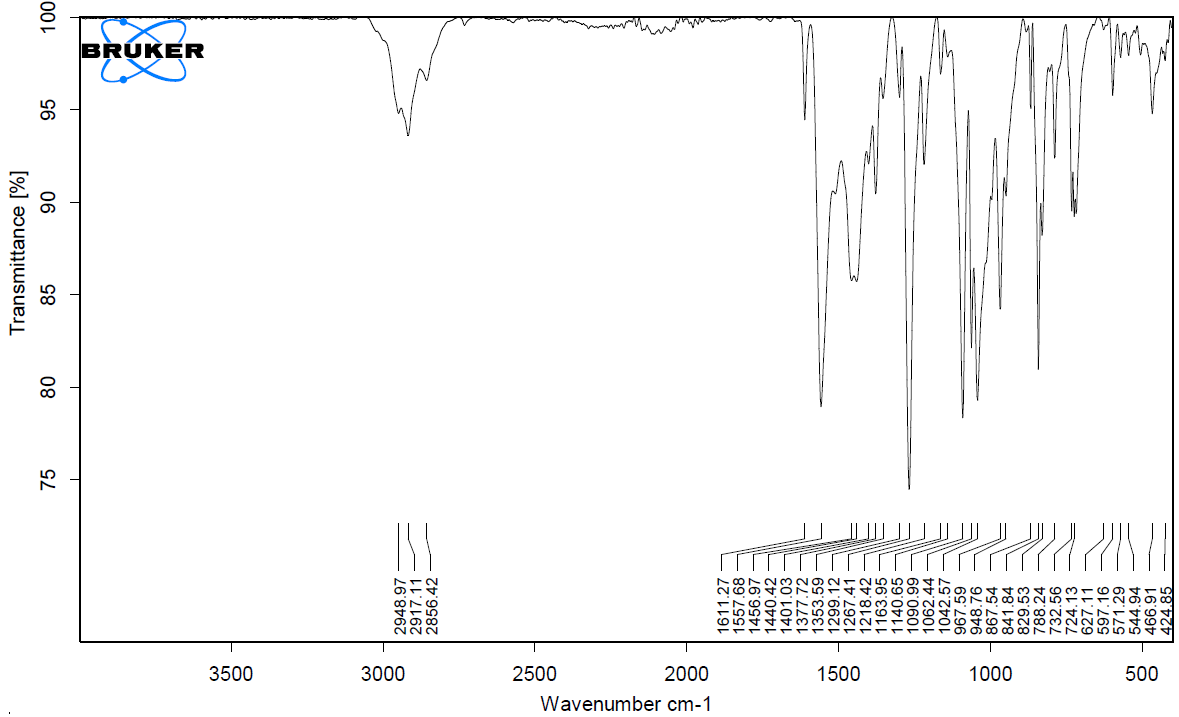


Figure S77: IR spectrum of compound 2_Me2_.

Figure S78: Solid-state UV/VIS spectrum of compound 2_Me2_.

Figure S79: Solution-state UV/VIS spectrum of compound 2_Me2_ (2 µM in toluene).

Figure S80: Solution-state PL spectrum of compound 2_Me2_ in toluene. Excitation with 405 nm continuous wave diode laser.

# 5. Synthesis and characterization of mixed substituted ^Mes^DPM triel complexes:

## 5.1 [(^Mes^DPM)Al(Me)I] (**3**_MeI_):

**^1^H-NMR** (300.19 MHz, C_6_D_6_) *δ* = 6.74 (m, 6 H, -C*H*(py), -C*H*(Mes)), 6.70 (s, 2 H, -‍C*H*(Mes)), 6.05 (d, ^3^*J*_AB_ = 4.0 Hz, 2 H, -C*H*(py)), 2.44 (s, 6 H, -*Me*), 2.38 (s, 3 H, -*Me*), 2.20 (s, 3 H, -*Me*), 2.13 (s, 9 H, -*Me*), 2.04 (s, 6 H, -*Me*), –0.84 (s, 3 H, -Al*Me*) ppm.

**^13^C{^1^H}-NMR** (75.48 MHz, C_6_D_6_) *δ* = 162.92 (s, 2 C, *C*_quart_ (pyr)), 146.96 (s, 1 C, *C*_quart_ (Mes)), 139.21 (s, 2 C, *C*_quart_ (Mes)), 139.16 (s, 2 C, *C*_quart_ (Mes)), 138.22 (s, 1 C, *C*_quart_ (Mes)), 137.98 (s, 1 C, *C*_quart_ (Mes)), 137.68 (s, 2 C, *C*_quart_ (Mes)), 137.15 (s, 2 C, *C*_quart_ (Mes)), 136.15 (s, 2 C, *C*_quart_ (Mes)), 134.37 (s, 2 C, -*C*H (Mes)), 133.59 (s, 2 C, *C*_quart_ (Mes)), 130.70 (s, 2 C, *C*_quart_ (pyr)), 129.17 (s, 2 C, -*C*H (pyr)), 128.59 (s, 2 C, -*C*H (Mes)), 128.35 (s, 2 C, -*C*H (Mes)), 120.85 (s, 2 C, -*C*H (pyr)), 22.20 (s, 2 C, -*Me*), 21.20 (s, 1 C, -‍*Me*), 21.17 (s, 2 C, -*Me*), 20.88 (s, 1 C, -*Me*), 20.45 (s, 2 C, -*Me*), 19.69 (s, 1 C, -*Me*), –‍6.16 (s, 1 C, -‍Al*Me*) ppm.

**^27^Al-NMR** (78.24 MHz, C_6_D_6_) *δ* = 69.89 (s, 1 Al, -*Al*(Me)I) ppm.

**IR** (cm^-1^) *ν* = 2949.22 (w), 2918.77 (w), 2856.28 (w), 1610.52 (m), 1547.58 (s), 1457.85 (m), 1378.42 (m), 1330.77 (m), 1314.66 (w), 1240.20 (s), 1211.47 (m), 1196.23 (m), 1153.85 (w), 1082.19 (m), 1050.49 (s), 1014.70 (s), 956.40 (m), 919.47 (m), 893.30 (w), 865.99 (m), 854.50 (m), 842.02 (s), 794.29 (m), 36.46 (m), 725.29 (m),663.43 (s), 620.26 (w), 583.25 (w), 571.59 (w), 492.40 (w),441.11 (m), 418.23 (w).

**MS (HR-CI^+^)** (m/z (%)) calculated for [C_37_H_40_N_2_Al_1_I_1_]^+^: 666.20515; found: 666.20258 (12.95).

**SS-UV/Vis** (BaSO_4_): *λ_max,ss_* = 524 nm.

**UV/Vis** (toluene): *λ_max_* = 514 nm, *ε_max_* (514 nm) = 1.05∙10^5^ L mol^-1^cm^-1^.

**PL** (toluene, *λ_Ex_* = 405 nm): *λ_F_* = 619 nm; *φ*_F_ = 0.2%.

Figure S81: ^1^H NMR spectrum of compound 3_MeI_ in C_6_D_6_ at 300 K.

Figure S82: ^13^C NMR spectrum of compound 3_MeI_ in C_6_D_6_ at 300 K.

Figure S83: ^27^Al NMR spectrum of compound 3_MeI_ in C_6_D_6_ at 300 K.


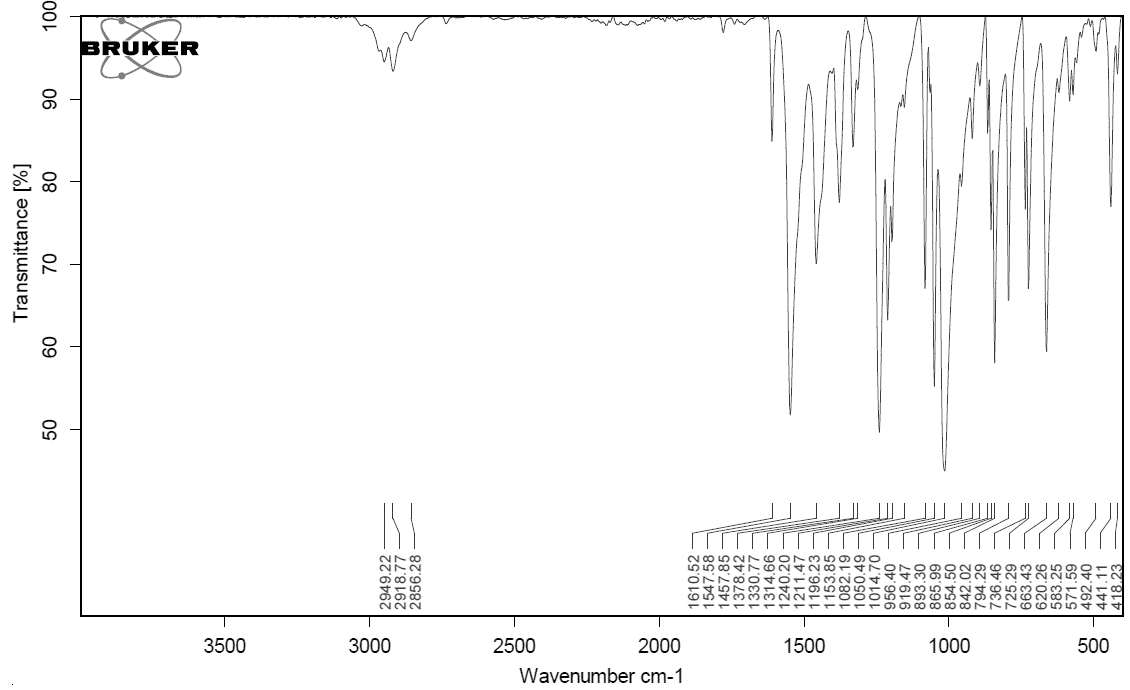


Figure S84: IR spectrum of compound 3_MeI_.

Figure S85: Solid-state UV/VIS spectrum of compound 3_MeI_.

Figure S86: Solution-state UV/VIS spectrum of compound 3_MeI_ (2 µM in toluene).

Figure S87: Solution-state PL spectrum of compound 3_MeI_ in toluene. Excitation with 405 nm continuous wave diode laser.

## 5.2 [(^Mes^DPM)Ga(Me)I] (**4**_MeI_):

**^1^H-NMR** (300.19 MHz, C_6_D_6_) *δ* = 6.81 (m, 4 H, -C*H*(py), -C*H*(Mes)), 6.70 (s, 4 H, -‍C*H*(Mes)), 6.08 (d, ^3^*J*_AB_ = 4.1 Hz, 2 H, -C*H*(py)), 2.44 (s, 6 H, -*Me*), 2.43 (s, 3 H, -*Me*), 2.20 (s, 3 H, -*Me*), 2.15 (s, 3 H, -*Me*), 2.10 (s, 6 H, -*Me*), 2.05 (s, 6 H, -*Me*), –0.39 (s, 3 H, -‍Ga*Me*) ppm.

**^13^C{^1^H}-NMR** (75.48 MHz, C_6_D_6_) *δ* = 161.79 (s, 2 C, *C*_quart_ (pyr)), 146.34 (s, 1 C, *C*_quart_ (Mes)), 139.03 (s, 2 C, *C*_quart_ (Mes)), 138.50 (s, 2 C, *C*_quart_ (Mes)), 138.16 (s, 1 C, *C*_quart_ (Mes)), 137.93 (s, 1 C, *C*_quart_ (Mes)), 137.93 (s, 2 C, *C*_quart_ (Mes)), 137.02 (s, 2 C, *C*_quart_ (Mes)), 136.19 (s, 1 C, *C*_quart_ (Mes)), 134.00 (s, 1 C, *C*_quart_ (Mes)), 133.71 (s, 2 C, -*C*H (Mes)), 130.89 (s, 2 C, *C*_quart_ (Mes)), 129.23 (s, 2 C, -*C*H (Mes)), 128.61 (s, 2 C, *C*_quart_ (pyr)), 128.25 (s, 2 C, -*C*H (pyr)), 128.01 (s, 2 C, -*C*H (Mes)), 120.04 (s, 2 C, -*C*H (pyr)), 22.14 (s, 2 C, -*Me*), 21.19 (s, 1 C, -*Me*), 21.14 (s, 2 C, -*Me*), 21.03 (s, 1 C, -*Me*), 20.32 (s, 2 C, -‍*Me*), 19.71 (s, 1 C, -*Me*), –2.44 (s, 1 C, -Ga*Me*) ppm.

**IR** (cm^-1^) *ν* = 2966.76 (w), 2917.50 (w), 2855.42 (w), 1610.27 (m), 1546.78 (s), 1459.78 (m), 1374.46 (m), 1334.53 (m), 1313.76 (w), 1244.00 (s), 1217.16 (m), 1153.85 (w), 1079.53 (m), 1064.98 (w), 1050.04 (m), 1009.70 (s), 917.86 (m), 890.94 (w), 865.54 (m), 853.96 (m), 839.51 (s), 790.46 (m), 732.61 (m), 723.20 (s), 621.08 (w), 582.66 (m), 512.16 (w), 468.85 (w), 420.56 (m).

**MS (HR-CI^+^)** (m/z (%)) calculated for [C_37_H_40_N_2_Ga_1_I_1_]^+^: 708.14920; found: 708.14977 (4.20), calculated for [C_37_H_41_N_2_Ga_1_I_1_]^+^: 709.15702; found: 709.15591 (8.42).

**CHNS**: calculated: C = 62.65%, H = 5.68%, N = 3.95%; found: C = 59.35%, H = 5.44%, N ‍= 3.93%.

**SS-UV/Vis** (BaSO_4_): *λ_max,ss_* = 521 nm.

**UV/Vis** (toluene): *λ_max_* = 514 nm, *ε_max_* (514 nm) = 1.08∙10^5^ L mol^-1^cm^-1^.

**PL** (toluene, *λ_Ex_* = 405 nm): *λ_F_* = 565 nm; *φ*_F_ = 0.5%.

Figure S88: ^1^H NMR spectrum of compound 4_MeI_ in C_6_D_6_ at 300 K.

Figure S89: ^13^C NMR spectrum of compound 4_MeI_ in C_6_D_6_ at 300 K.


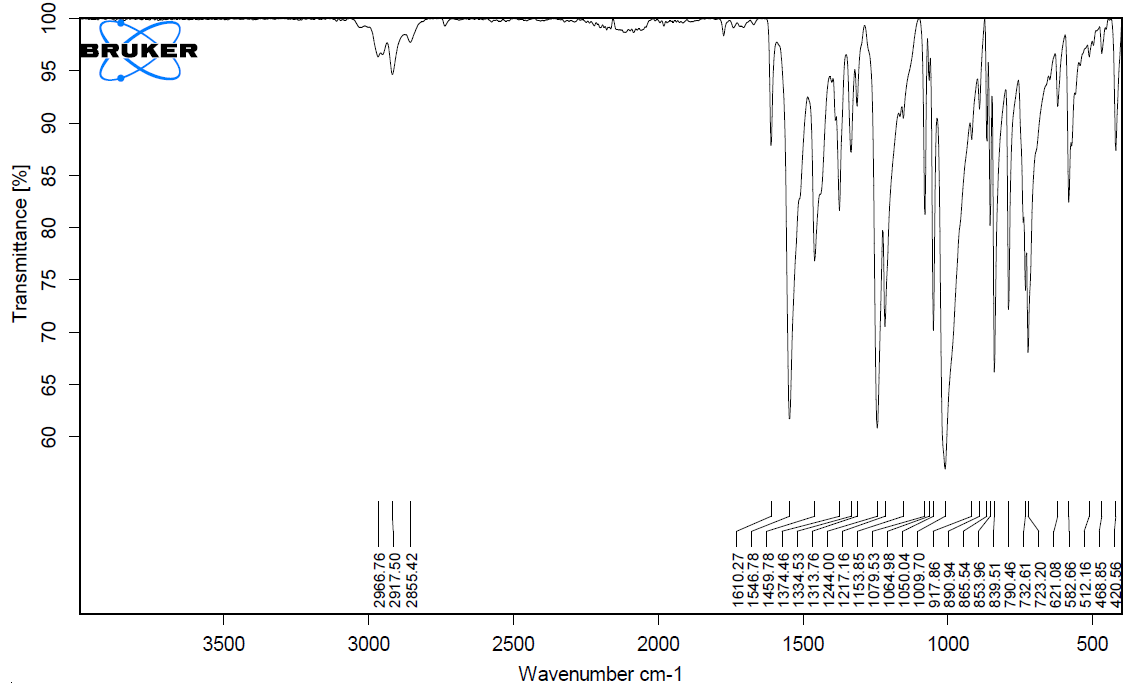


Figure S90: IR spectrum of compound 4_MeI_.

Figure S91: Solid-state UV/VIS spectrum of compound 4_MeI_.

Figure S92: Solution-state UV/VIS spectrum of compound 4_MeI_ (2 µM in toluene).

Figure S93: Solution-state PL spectrum of compound 4_MeI_ in toluene. Excitation with 405 nm continuous wave diode laser.

## 5.3 [(^Mes^DPM)In(Me)I] (**5**_MeI_):

**^1^H-NMR** (300.19 MHz, C_6_D_6_) *δ* = 6.79 (m, 4 H, -C*H*(Mes), -C*H*(py)), 6.71 (s, 4 H, -‍C*H*(Mes)), 6.14 (d, ^3^*J*_AB_ = 4.1 Hz, 2 H, -C*H*(py)), 2.39 (s, 6 H, -*Me*), 2.38 (s, 3 H, -*Me*), 2.21 (s, 3 H, -*Me*), 2.20 (s, 3 H, -*Me*), 2.10 (s, 6 H, -*Me*), 2.03 (s, 6 H, -*Me*), –0.27 (s, 3 H, -‍In*Me*) ppm.

**^13^C{^1^H}-NMR** (75.48 MHz, C_6_D_6_) *δ* = 162.07 (s, 2 C, *C*_quart_ (pyr)), 146.75 (s, 1 C, *C*_quart_ (Mes)), 139.473(s, 2 C, *C*_quart_ (Mes)), 139.11 (s, 2 C, *C*_quart_ (Mes)), 138.18 (s, 2 C, *C*_quart_ (Mes)), 137.93 (s, 1 C, *C*_quart_ (Mes)), 137.80 (s, 1 C, *C*_quart_ (Mes)), 136.88 (s, 2 C, *C*_quart_ (Mes)), 136.17 (s, 1 C, *C*_quart_ (Mes)), 135.30 (s, 1 C, *C*_quart_ (Mes)), 134.24 (s, 2 C, -*C*H (Mes)), 131.62 (s, 2 C, *C*_quart_ (Mes)), 129.35 (s, 2 C, -*C*H (Mes)), 128.35 (s, 2 C, *C*_quart_ (pyr)), 128.16 (s, 2 C, -*C*H (pyr)), 127.97 (s, 2 C, -*C*H (Mes)), 119.32 (s, 2 C, -*C*H (pyr)), 22.00 (s, 2 C, -*Me*), 21.21 (s, 1 C, -*Me*), 21.14 (s, 2 C, -*Me*), 20.70 (s, 1 C, -*Me*), 20.35 (s, 2 C, -‍*Me*), 19.84 (s, 1 C, -*Me*), –5.14 (s, 1 C, -In*Me*) ppm.

**IR** (cm^-1^) *ν* = 2968.28 (w), 2917.79 (w), 2855.25 (w), 1611.10 (m), 1543.94 (s), 1459.41 (m), 1371.98 (m), 1331.15 (m), 1310.68 (w), 1241.91 (s), 1217.82 (m), 1165.30 (w), 1077.64 (w), 1051.35 (m), 1002.60 (s), 914.22 (w), 864.19 (m), 852.95 (m), 838.78 (s), 790.52 (m), 726.35 (m), 710.53 (m), 618.74 (w), 572.50 (w), 558.49 (w), 505.46 (m), 465.85 (w), 408.56 (w).

**MS (HR-CI^+^)** (m/z (%)) calculated for [C_37_H_40_N_2_In_1_I_1_]^+^: 754.12749; found: 754.12403 (37.61), calculated for [C_37_H_41_N_2_In_1_I_1_]^+^: 755.13532; found: 755.13532 (35.39).

**SS-UV/Vis** (BaSO_4_): *λ_max,ss_* = 512 nm.

**UV/Vis** (toluene): *λ_max_* = 508 nm, *ε_max_* (508 nm) = 1.03∙10^5^ L mol^-1^cm^-1^.

**PL** (toluene, *λ_Ex_* = 405 nm): *λ_F_* = 560 nm; *φ*_F_ = 0.6%.

Figure S94: ^1^H NMR spectrum of compound 5_MeI_ in C_6_D_6_ at 300 K.

Figure S95: ^13^C NMR spectrum of compound 5_MeI_ in C_6_D_6_ at 300 K.


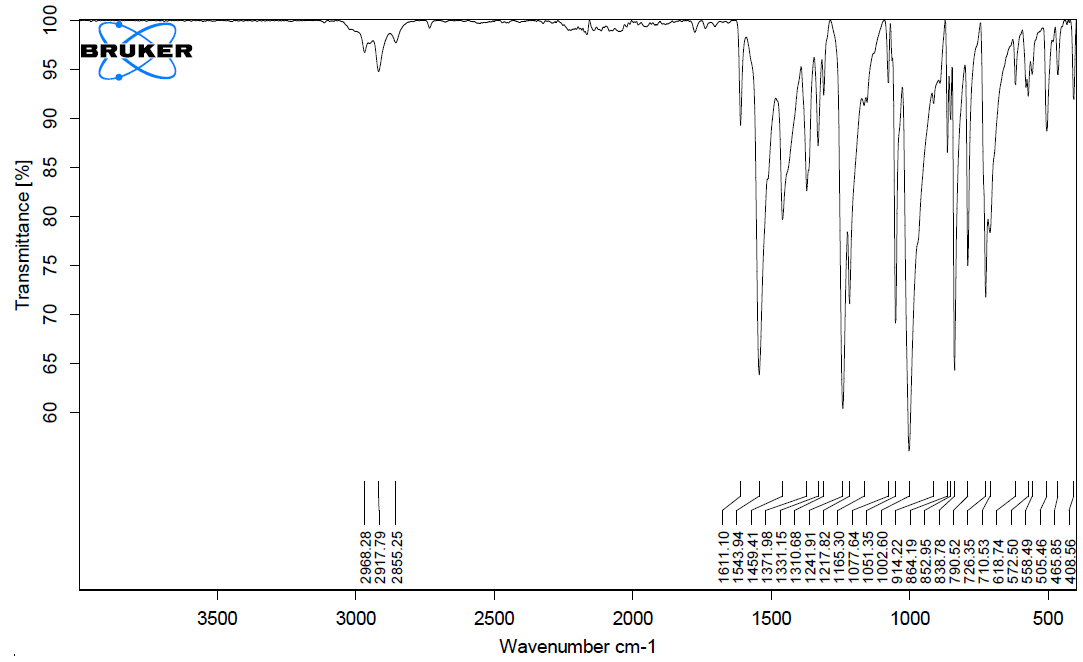


Figure S96: IR spectrum of compound 5_MeI_.

Figure S97: Solid-state UV/VIS spectrum of compound 5_MeI_.

Figure S98: Solution-state UV/VIS spectrum of compound 5_MeI_ (2 µM in toluene).

Figure S99: Solution-state PL spectrum of compound 5_MeI_ in toluene. Excitation with 405 nm continuous wave diode laser.

## 5.4 [(^Mes^DPM)Al(Me)Cl] (**3**_MeCl_):

**^1^H-NMR** (300.19 MHz, C_6_D_6_) *δ* = 6.77 (m, 2 H, -C*H*(Mes)), 6.73 (s, 4 H, -C*H*(Mes)), 6.67 (d, ^3^*J*_AB_ = 4.1 Hz, 2 H, -C*H*(py)), 6.04 (d, ^3^*J*_AB_ = 4.0 Hz, 2 H, -C*H*(py)), 2.38 (s, 6 H, -‍*Me*), 2.24 (s, 3 H, -*Me*), 2.20 (s, 3 H, -‍*Me*), 2.13 (s, 3 H, -*Me*), 2.12 (s, 6 H, -*Me*), 2.05 (s, 6 H, -*Me*), –0.95 (s, 3 H, -Al*Me*) ppm.

**^13^C{^1^H}-NMR** (75.48 MHz, C_6_D_6_) *δ* = 163.20 (s, 2 C, *C*_quart_ (pyr)), 146.79 (s, 1 C, *C*_quart_ (Mes)), 139.20 (s, 2 C, *C*_quart_ (Mes)), 139.04 (s, 2 C, *C*_quart_ (Mes)), 138.17 (s, 2 C, *C*_quart_ (Mes)), 138.11 (s, 1 C, *C*_quart_ (Mes)), 137.51 (s, 1 C, *C*_quart_ (Mes)), 137.24 (s, 2 C, *C*_quart_ (Mes)), 136.30 (s, 1 C, *C*_quart_ (Mes)), 133.83 (s, 1 C, *C*_quart_ (Mes)), 133.71 (s, 2 C, -*C*H (pyr)), 130.89 (s, 2 C, *C*_quart_ (pyr)), 128.82 (s, 2 C, -*C*H (Mes)), 128.35 (s, 2 C, -*C*H (Mes)), 127.95 (s, 2 C, -*C*H (Mes)), 120.57 (s, 2 C, -*C*H (pyr)), 21.18 (m, 3 C, -*Me*), 21.15 (s, 2 C, -‍*Me*), 20.39 (s, 2 C, -*Me*), 20.05 (s, 1 C, -*Me*), 19.78 (s, 1 C, -*Me*), –10.37 (s, 1 C, -‍Al*Me*) ppm.

**^27^Al-NMR** (78.24 MHz, C_6_D_6_) *δ* = 71.06 (s, 1 Al, -*Al*(Me)Cl) ppm.

**IR** (cm^-1^) *ν* = 2972.82 (w), 2948.13 (w), 2920.11 (w), 2859.04 (w), 1610.92 (m), 1549.99 (s), 1460.15 (m), 1379.29 (m), 1330.98 (m), 1313.77 (m), 1241.99 (s), 1212.15 (m), 1196.35 (m), 1153.58 (m), 1081.49 (m), 1051.41 (m), 1013.94 (s), 919.97 (m), 892.45 (m), 866.08 (m), 855.89 (m), 841.63 (m), 794.39 (m), 737.37 (m), 725.64 (m), 658.93 (m), 620.78 (m), 583.96 (w), 572.13 (w), 519.54 (w), 463.04 (m), 415.99 (m).

**MS (HR-CI^+^)** (m/z (%)) calculated for [C_37_H_40_N_2_Al_1_Cl_1_]^+^: 574.26954; found: 574.26804 (12.99).

**SS-UV/Vis** (BaSO_4_): *λ_max,ss_* = 516 nm.

**UV/Vis** (toluene): *λ_max_* = 509 nm, *ε_max_* (509 nm) = 1.06∙10^5^ L mol^-1^cm^-1^.

**PL** (toluene, *λ_Ex_* = 405 nm): *λ_F_* = 587 nm; *φ*_F_ = 0.6%.

Figure S100: ^1^H NMR spectrum of compound 3_MeCl_ in C_6_D_6_ at 300 K.

Figure S101: ^13^C NMR spectrum of compound 3_MeCl_ in C_6_D_6_ at 300 K.

Figure S102: ^27^Al NMR spectrum of compound 3_MeCl_ in C_6_D_6_ at 300 K.


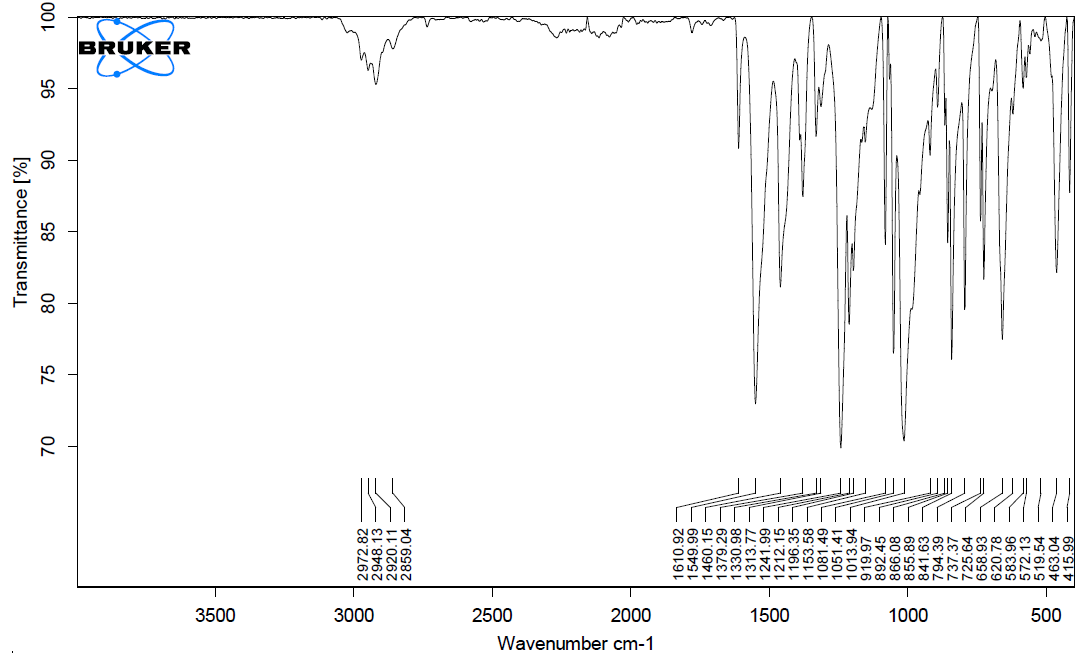


Figure S103: IR spectrum of compound 3_MeCl_.

Figure S104: Solid-state UV/VIS spectrum of compound 3_MeCl_.

Figure S105: Solution-state UV/VIS spectrum of compound 3_MeCl_ (2 µM in toluene).

Figure S106: Solution-state PL spectrum of compound 3_MeCl_ in toluene. Excitation with 405 nm continuous wave diode laser.

## 5.5 [(^Mes^DPM)Ga(Me)Cl] (**4**_MeCl_):

**^1^H-NMR** (300.19 MHz, C_6_D_6_) *δ* = 6.79 (m, 2 H, -C*H*(Mes)), 6.72 (m, 6 H, -C*H*(Mes), -‍C*H*(py)), 6.08 (d, ^3^*J*_AB_ = 4.0 Hz, 2 H, -C*H*(py)), 2.39 (s, 6 H, -*Me*), 2.30 (s, 3 H, -*Me*), 2.20 (s, 3 H, -*Me*), 2.16 (s, 3 H, -*Me*), 2.08 (s, 6 H, -*Me*), 2.06 (s, 6 H, -*Me*), –0.61 (s, 3 H, -‍Ga*Me*) ppm.

**^13^C{^1^H}-NMR** (75.48 MHz, C_6_D_6_) *δ* = 162.08 (s, 2 C, *C*_quart_ (pyr)), 146.39 (s, 1 C, *C*_quart_ (Mes)), 138.95 (s, 2 C, *C*_quart_ (Mes)), 138.54 (s, 2 C, *C*_quart_ (Mes)), 138.42 (s, 2 C, *C*_quart_ (Mes)), 138.04 (s, 1 C, *C*_quart_ (Mes)), 137.83 (s, 1 C, *C*_quart_ (Mes)), 137.00 (s, 2 C, *C*_quart_ (Mes)), 136.22 (s, 1 C, *C*_quart_ (Mes)), 134.14 (s, 1 C, -*C*H (Mes)), 133.28 (s, 2 C, -*C*H (pyr)), 131.08 (s, 2 C, *C*_quart_ (Mes)), 128.95 (s, 2 C, *C*_quart_ (pyr)), 128.48 (s, 2 C, -*C*H (Mes)), 127.89 (s, 2 C, -*C*H (Mes)), 119.78 (s, 2 C, -*C*H (pyr)), 21.16 (s, 5 C, -*Me*), 20.25 (s, 2 C, -‍*Me*), 20.18 (s, 1 C, -*Me*), 19.80 (s, 1 C, -*Me*), –7.54 (s, 1 C, -Ga*Me*) ppm.

**IR** (cm^-1^) *ν* = 2971.39 (w), 2917.60 (w), 2858.31 (w), 1610.74 (m), 1548.20 (s), 1461.88 (m), 1376.50 (m), 1335.31 (m), 1315.72 (w), 1246.79 (s), 1218.59 (m), 1165.72 (w), 1079.49 (m), 1066.10 (m), 1050.60 (m), 1010.63 (s), 918.76 (m), 890.83 (w), 865.72 (w), 855.39 (m), 839.69 (s), 790.85 (m), 734.01 (m), 722.68 (m), 621.47 (w), 583.11 (m), 468.32 (w), 421.15 (m).

**MS (HR-CI^+^)** (m/z (%)) calculated for [C_37_H_40_N_2_Ga_1_Cl_1_]^+^: 616.21358; found: 616.21209 (14.86), calculated for [C_37_H_40_N_2_Ga_1_]^+^: 581.24473; found: 581.24359 (100.00).

**SS-UV/Vis** (BaSO_4_): *λ_max,ss_* = 506 nm.

**UV/Vis** (toluene): *λ_max_* = 507 nm, *ε_max_* (507 nm) = 1.72∙10^5^ L mol^-1^cm^-1^.

**PL** (toluene, *λ_Ex_* = 405 nm): *λ_F_* = 554 nm; *φ*_F_ = 39%.

Figure S107: ^1^H NMR spectrum of compound 4_MeCl_ in C_6_D_6_ at 300 K.

Figure S108: ^13^C NMR spectrum of compound 4_MeCl_ in C_6_D_6_ at 300 K.


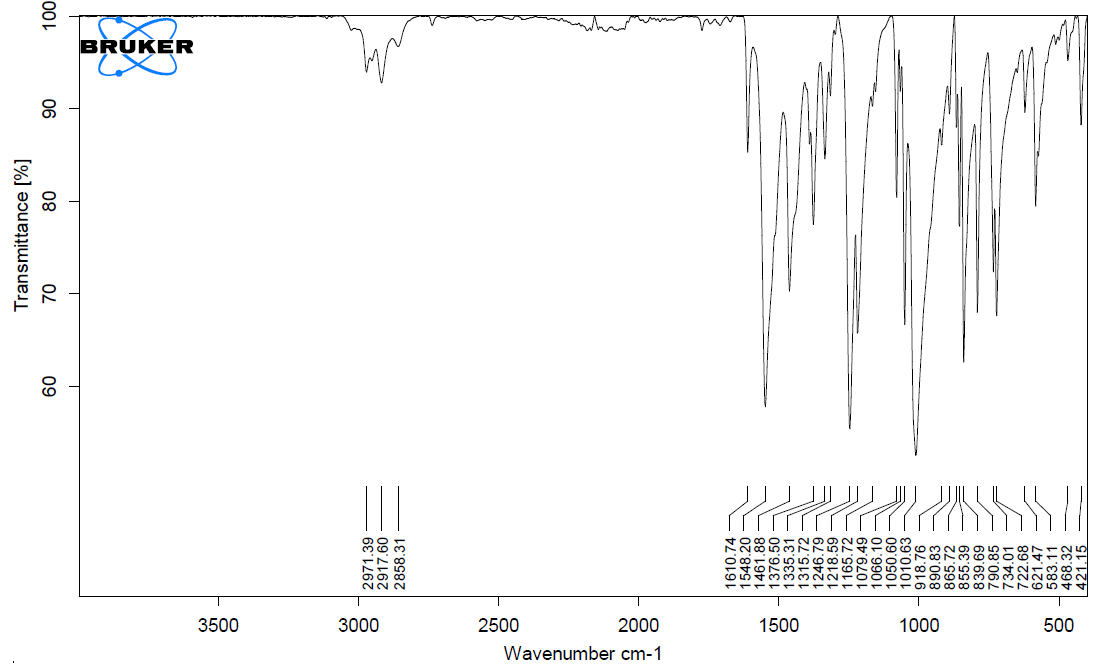


Figure S109: IR spectrum of compound 4_MeCl_.

Figure S110: Solid-state UV/VIS spectrum of compound 4_MeCl_.

Figure S111: Solution-state UV/VIS spectrum of compound 4_MeCl_ (2 µM in toluene).

Figure S112: Solution-state PL spectrum of compound 4_MeCl_ in toluene. Excitation with 405 nm continuous wave diode laser.

## 5.6 [(^Mes^DPM)In(Me)Cl] (**5**_MeCl_):

**^1^H-NMR** (300.19 MHz, C_6_D_6_) *δ* = 6.82 (m, 2 H, -C*H*(Mes)), 6.77 (d, ^3^*J*_AB_ = 3.9 Hz, 2 H, -‍C*H*(py)), 6.70 (s, 4 H, -C*H*(Mes)), 6.13 (d, ^3^*J*_AB_ = 4.0 Hz, 2 H, -C*H*(py)), 2.38 (s, 6 H, -*Me*), 2.29 (s, 3 H, -*Me*), 2.21 (s, 6 H, -‍*Me*), 2.05 (s, 6 H, -*Me*), 2.04 (s, 6 H, -*Me*), –0.45 (s, 3 H, -‍In*Me*) ppm.

**^13^C{^1^H}-NMR** (75.48 MHz, C_6_D_6_) *δ* = 162.30 (s, 2 C, *C*_quart_ (pyr)), 146.87 (s, 1 C, *C*_quart_ (Mes)), 139.63 (s, 2 C, *C*_quart_ (Mes)), 139.09 (s, 2 C, *C*_quart_ (Mes)), 138.80 (s, 1 C, *C*_quart_ (Mes)), 137.80 (s, 2 C, *C*_quart_ (Mes)), 136.83 (s, 1 C, *C*_quart_ (Mes)), 136.24 (s, 1 C, *C*_quart_ (Mes)), 135.34 (s, 1 C, *C*_quart_ (Mes)), 134.07 (s, 2 C, -*C*H (pyr)), 131.74 (s, 2 C, *C*_quart_ (pyr)), 129.37 (s, 1 C, *C*_quart_ (Mes)), 129.20 (s, 2 C, -*C*H (Mes)), 128.25 (s, 2 C, -*C*H (Mes)), 127.85 (s, 2 C, -*C*H (Mes)), 127.52 (s, 1 C, *C*_quart_ (Mes)), 119.13 (s, 2 C, -*C*H (pyr)), 21.20 (s, 1 C, -*Me*), 21.14 (s, 4 C, -*Me*), 21.08 (s, 1 C, -*Me*), 20.21 (s, 1 C, -*Me*), 20.10 (s, 1 C, -*Me*), 19.89 (s, 1 C, -‍*Me*), –9.66 (s, 1 C, -In*Me*) ppm.

**IR** (cm^-1^) *ν* = 2970.57 (w), 2917.97 (w), 2857.52 (w), 1610.87 (m), 1542.45 (s), 1461.87 (m), 1373.12 (m), 1333.55 (m), 1311.31 (w), 1242.53 (s), 1220.58 (m), 1077.66 (w), 1050.05 (m), 1005.01 (s), 890.40 (w), 864.68 (m), 855.21 (m), 838.83 (s), 789.77 (m), 725.24 (m), 619.63 (w), 572.62 (w), 520.12 (w), 466.31 (w), 409.75 (w).

**MS (HR-CI^+^)** (m/z (%)) calculated for [C_37_H_40_N_2_In_1_Cl_1_]^+^: 662.19188; found: 662.19223 (15.93).

**CHNS**: calculated: C = 67.03%, H = 6.08%, N = 4.23%; found: C = 64.38%, H = 5.89%, N = 4.48%.

**SS-UV/Vis** (BaSO_4_): *λ_max,ss_* = 513 nm.

**UV/Vis** (toluene): *λ_max_* = 504 nm, *ε_max_* (504 nm) = 1.17∙10^5^ L mol^-1^cm^-1^.

**PL** (toluene, *λ_Ex_* = 405 nm): *λ_F_* = 592 nm; *φ*_F_ = 0.9%.

Figure S113: ^1^H NMR spectrum of compound 5_MeCl_ in C_6_D_6_ at 300 K.

Figure S114: ^13^C NMR spectrum of compound 5_MeCl_ in C_6_D_6_ at 300 K.


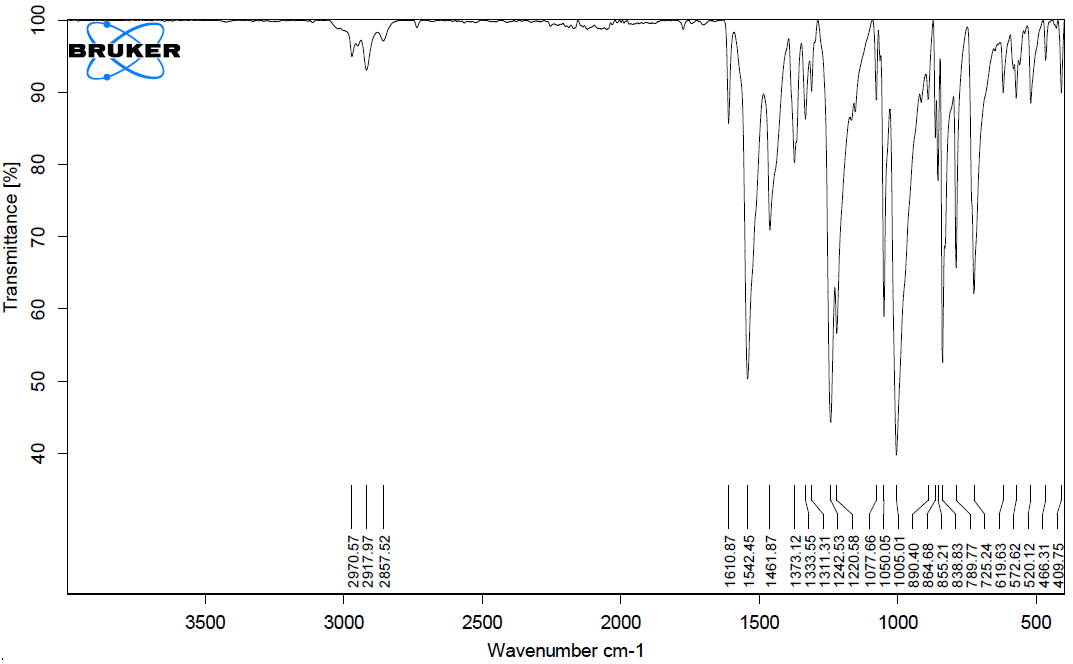


Figure S115: IR spectrum of compound 5_MeCl_.

Figure S116: Solid-state UV/VIS spectrum of compound 5_MeCl_.

Figure S117: Solution-state UV/VIS spectrum of compound 5_MeCl_ (2 µM in toluene).

Figure S118: Solution-state PL spectrum of compound 5_MeCl_ in toluene. Excitation with 405 nm continuous wave diode laser.

## 5.7 [(^Mes^DPM)Al(Me)Br] (**3**_MeBr_):

**^1^H-NMR** (300.19 MHz, C_6_D_6_) *δ* = 6.77 (m, 2 H,-C*H*(Mes)), 6.73 (s, 2 H, -C*H*(Mes)), 6.72 (s, 2 H, -‍C*H*(Mes)), 6.68 (d, ^3^*J*_AB_ = 4.0 Hz, 2 H, -C*H*(py)), 6.04 (d, ^3^*J*_AB_ = 4.0 Hz, 2 H, -C*H*(py)), 2.41 (s, 6 H, -*Me*), 2.29 (s, 3 H, -*Me*), 2.20 (s, 3 H, -*Me*), 2.12 (s, 9 H, -*Me*), 2.05 (s, 6 H, -*Me*), –0.92 (s, 3 H, -Al*Me*) ppm.

**^13^C{^1^H}-NMR** (125.79 MHz, C_6_D_6_) *δ* = 163.11 (s, 2 C, *C*_quart_ (pyr)), 146.87 (s, 1 C, *C*_quart_ (Mes)), 139.21 (s, 2 C, *C*_quart_ (Mes)), 139.09 (s, 2 C, *C*_quart_ (Mes)), 138.15 (s, 1 C, *C*_quart_ (Mes)), 137.95 (s, 2 C, *C*_quart_ (Mes)), 137.72 (s, 1 C, *C*_quart_ (Mes)), 137.20 (s, 2 C, *C*_quart_ (Mes)), 136.19 (s, 1 C, *C*_quart_ (pyr)), 134.00 (s, 2 C, -*C*H (pyr)), 133.72 (s, 1 C, *C*_quart_ (pyr)), 130.82 (s, 2 C, -*C*H (Mes)), 128.96 (s, 2 C, *C*_quart_ (Mes)), 128.50 (s, 2 C, -*C*H (Mes)), 128.35 (s, 2 C, -*C*H (Mes)), 120.68 (s, 2 C, -*C*H (pyr)), 21.54 (s, 2 C, -*Me*), 21.18 (s, 1 C, -‍*Me*), 21.17 (s, 2 C, -*Me*), 20.40 (s, 2 C, -*Me*), 20.26 (s, 1 C, -*Me*), 19.74 (s, 1 C, -*Me*), –‍8.96 (s, 1 C, -‍Al*Me*) ppm.

**^27^Al-NMR** (78.24 MHz, C_6_D_6_) *δ* = 70.00 (s, 1 Al, -*Al*(Me)Br) ppm.

**IR** (cm^-1^) *ν* = 2948.89 (w), 2919.56 (w), 2856.47 (w), 1610.92 (w), 1550.68 (s), 1459.67 (m), 1379.48 (m), 1331.40 (m), 1243.57 (s), 1212.47 (m), 1197.05 (m), 1082.17 (m), 1051.72 (s), 1019.07 (s), 956.52 (m), 920.20 (w), 893.01 (w), 866.26 (w), 855.97 (m), 842.62 (m), 794.97 (m), 737.27 (m), 725.92 (m), 661.09 (m), 620.80 (w), 584.11 (w), 571.52 (w), 444.95 (m), 417.49 (w).

**MS (HR-CI^+^)** (m/z (%)) calculated for [C_37_H_40_N_2_Al_1_Br_1_]^+^: 618.21902; found: 618.21769 (23.73).

**SS-UV/Vis** (BaSO_4_): *λ_max,ss_* = 518 nm.

**UV/Vis** (toluene): *λ_max_* = 511 nm, *ε_max_* (511 nm) = 1.21∙10^5^ L mol^-1^cm^-1^.

**PL** (toluene, *λ_Ex_* = 405 nm): *λ_F_* = 553 nm; *φ*_F_ = 29.0%.

Figure S119: ^1^H NMR spectrum of compound 3_MeBr_ in C_6_D_6_ at 300 K.

***Figure S120: ^13^C NMR spectrum of compound 3_MeBr_*** ***in C_6_D_6_ at 300 K.***

Figure S121: ^27^Al NMR spectrum of compound 3_MeBr_ in C_6_D_6_ at 300 K.


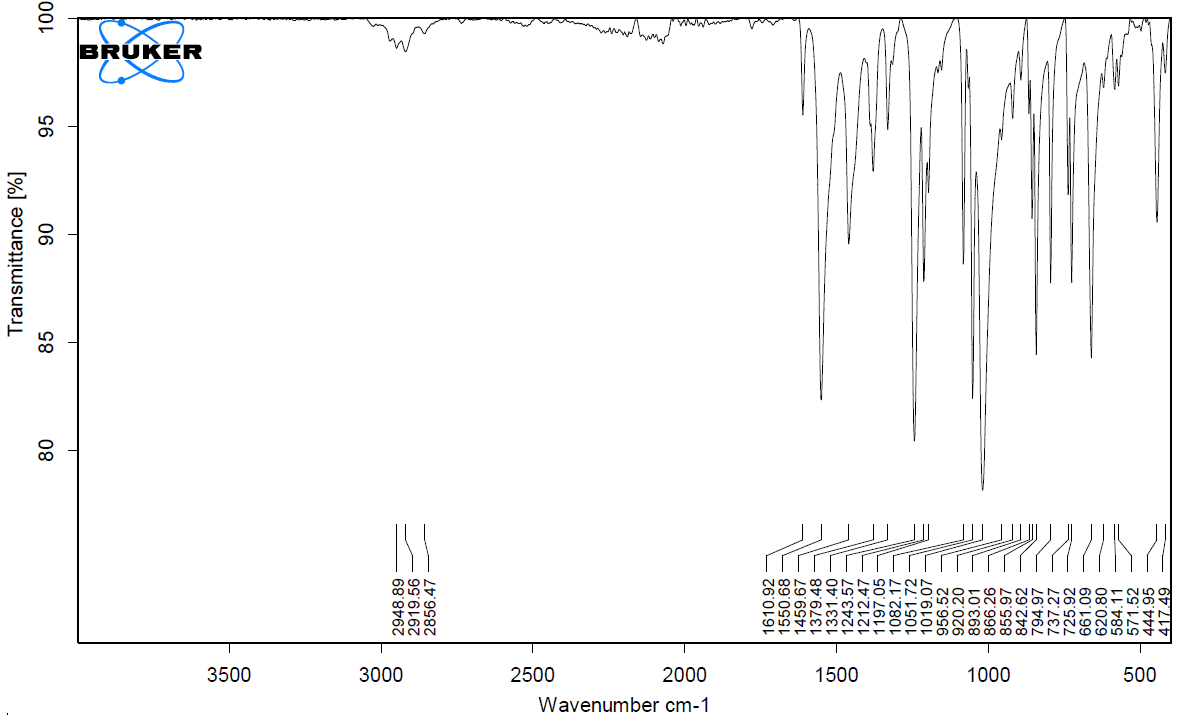


Figure S122: IR spectrum of compound 3_MeBr_.

Figure S123: Solid-state UV/VIS spectrum of compound 3_MeBr_.

Figure S124: Solution-state UV/VIS spectrum of compound 3_MeBr_ (2 µM in toluene).

***Figure S125: Solution state PL spectrum of compound 3_MeBr_ in toluene. Excitation with 405 nm continuous wave diode laser.***

## 5.8 [(^Mes^DPM)Ga(Me)Br] (**4**_MeBr_):

**^1^H-NMR** (300.19 MHz, C_6_D_6_) *δ* = 6.81 (s, 1 H, -C*H*(Mes)), 6.76 (s, 1 H, -C*H*(Mes)), 6.73 (d, ^3^*J*_AB_ = 4.1 Hz, 2 H, -C*H*(py)), 6.71 (s, 4 H, -C*H*(Mes)), 6.13 (d, ^3^*J*_AB_ = 4.1 Hz, 2 H, -C*H*(py)), 2.41 (s, 6 H, -*Me*), 2.36 (s, 3 H, -*Me*), 2.20 (s, 3 H, -*Me*), 2.16 (s, 3 H, -*Me*), 2.09 (s, 6 H, -*Me*), 2.05 (s, 6 H, -*Me*), –0.53 (s, 3 H, -‍Ga*Me*) ppm.

**^13^C{^1^H}-NMR** (75.49 MHz, C_6_D_6_) *δ* = 161.97 (s, 2 C, *C*_quart_ (pyr)), 146.39 (s, 1 C, *C*_quart_ (Mes)), 138.98 (s, 2 C, *C*_quart_ (Mes)), 138.53 (s, 2 C, *C*_quart_ (Mes)), 138.21 (s, 2 C, *C*_quart_ (Mes)), 138.06 (s, 1 C, *C*_quart_ (Mes)), 137.98 (s, 1 C, *C*_quart_ (Mes)), 137.01 (s, 2 C, *C*_quart_ (Mes)), 136.16 (s, 1 C, *C*_quart_ (pyr)), 134.06 (s, 1 C, *C*_quart_ (pyr)), 133.46 (s, 2 C, *C*_quart_ (Mes)), 131.00 (s, 2 C, -*C*H (Mes)), 129.06 (s, 2 C, -*C*H (pyr)), 127.92 (s, 2 C, -‍*C*H (Mes)), 127.46 (s, 2 C, -*C*H (Mes)), 119.88 (s, 2 C, -*C*H (pyr)), 21.51 (s, 2 C, -*Me*), 21.19 (s, 1 C, -‍*Me*), 21.15 (s, 2 C, -*Me*), 20.41 (s, 1 C, -*Me*), 20.27 (s, 2 C, -*Me*), 19.76 (s, 1 C, -*Me*), –‍5.67 (s, 1 C, -‍In*Me*) ppm.

**IR** (cm^-1^) *ν* = 2969.49 (w), 2917.01 (w), 2856.97 (w), 1610.23 (m), 1548.12 (s, br), 1461.11 (m), 1375.66 (m), 1334.47 (m), 1314.97 (m), 1245.42 (s), 1217.41 (m), 1079.33 (m), 1050.33 (m), 1011.20 (s), 918.31 (m), 865.61 (m), 854.91 (m), 839.89 (s), 827.79 (m), 790.78 (m), 733.33 (m), 723.05 (m), 621.13 (m), 582.71 (m), 572.75 (m), 508.92 (w), 469.31 (w), 420.80 (m).

**MS (HR-CI^+^)** (m/z (%)) calculated for [C_37_H_40_N_2_Ga_1_Br_1_]^+^: 660.16307; found: 660.16242 (16.33).

**CHNS**: calculated: C = 67.09%, H = 6.09%, N = 4.23%; found: C =66.54%, H = 5.96%, N ‍= 4.26%.

**SS-UV/Vis** (BaSO_4_): *λ_max,ss_* = 511 nm.

**UV/Vis** (toluene): *λ_max_* = 510 nm, *ε_max_* (510 nm) = 0.95∙10^5^ L mol^-1^cm^-1^.

**PL** (toluene, *λ_Ex_* = 405 nm): *λ_F_* = 553 nm; *φ*_F_ = 23.8%.

Figure S126: ^1^H NMR spectrum of compound 4_MeBr_ in C_6_D_6_ at 300 K.

Figure S127: ^13^C NMR spectrum of compound 4_MeBr_ in C_6_D_6_ at 300 K.


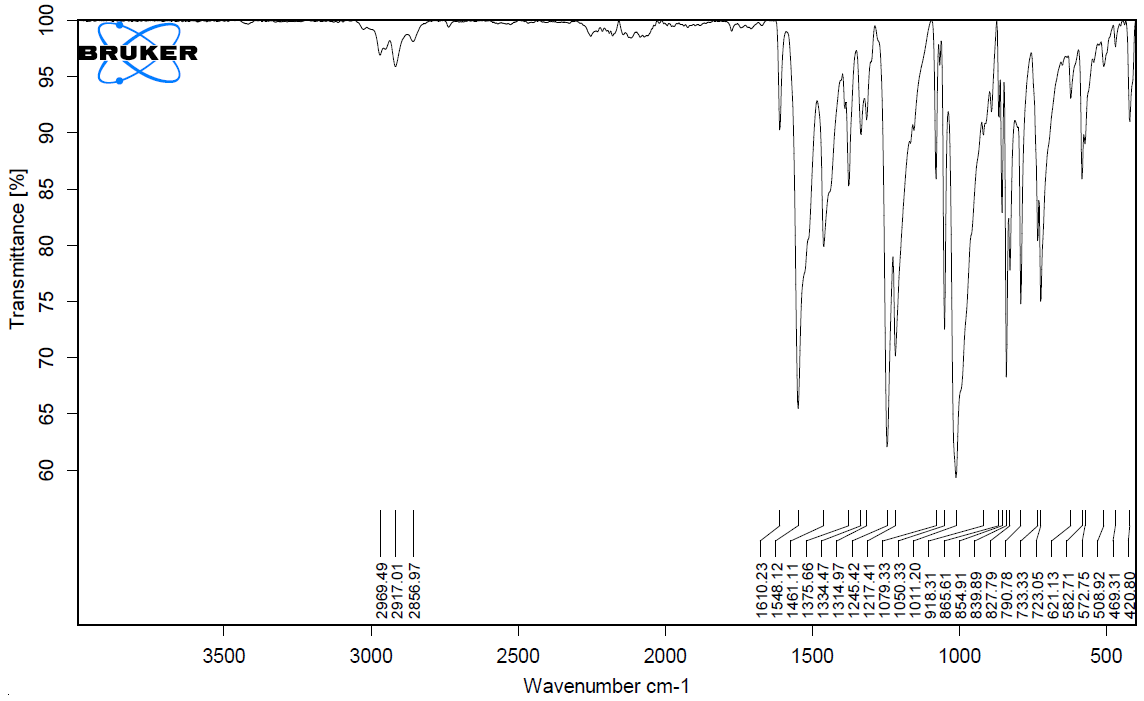


Figure S128: IR spectrum of compound 4_MeBr_.

Figure S129: Solid state UV/Vis spectrum of compound 4_MeBr_.

Figure S130: Solution state UV/Vis spectrum of compound 4_MeBr_ (2 µM in toluene).

***Figure S131: Solution state PL spectrum of compound 4_MeBr_ in toluene. Excitation with 405 nm continuous wave diode laser.***

## 5.9 [(^Mes^DPM)In(Me)Br] (**5**_MeBr_):

**^1^H-NMR** (300.19 MHz, C_6_D_6_) *δ* = 6.77 (m, 4 H, -C*H*(py), -C*H*(Mes)),), 6.71 (s, 4 H, -‍C*H*(Mes)), 6.13 (d, ^3^*J*_AB_ = 4.0 Hz, 2 H, -C*H*(py)), 2.38 (s, 6 H, -*Me*), 2.32 (s, 3 H, -*Me*), 2.21 (s, 6 H, -*Me*), 2.08 (s, 6 H, -*Me*), 2.04 (s, 6 H, -*Me*), –0.38 (s, 3 H, -In*Me*) ppm.

**^13^C{^1^H}-NMR** (75.49 MHz, C_6_D_6_) *δ* = 162.22 (s, 2 C, *C*_quart_ (pyr)), 146.85 (s, 1 C, *C*_quart_ (Mes)), 139.57 (s, 2 C, *C*_quart_ (Mes)), 139.11 (s, 2 C, *C*_quart_ (Mes)), 138.57 (s, 2 C, *C*_quart_ (Mes)), 137.87 (s, 1 C, *C*_quart_ (Mes)), 137.81 (s, 1 C, *C*_quart_ (Mes)), 136.86 (s, 2 C, *C*_quart_ (Mes)), 136.20 (s, 1 C, *C*_quart_ (pyr)), 135.32 (s, 1 C, *C*_quart_ (pyr)), 134.14 (s, 2 C, -*C*H (pyr)), 131.70 (s, 2 C, *C*_quart_ (Mes)), 129.26 (s, 2 C, -*C*H (Mes)), 128.19 (s, 2 C, -‍*C*H (Mes)), 128.02 (s, 2 C, -*C*H (Mes)), 119.21 (s, 2 C, -*C*H (pyr)), 21.37 (s, 2 C, -*Me*), 21.20 (s, 1 C, -‍*Me*), 21.14 (s, 2 C, -*Me*), 20.26 (s, 3 C, -*Me*), 19.87 (s, 1 C, -*Me*), –7.95 (s, 1 C, -In*Me*) ppm.

**IR** (cm^-1^) *ν* = 2969.09 (w), 2917.09 (w), 2854.92 (w), 1610.95 (m), 1541.34 (s), 1458.96 (m), 1372.01 (m), 1331.68 (m), 1311.07 (w), 1241.42 (s), 1218.41 (s), 1103.79 (m), 1077.52 (w), 1050.79 (m), 1003.54 (s), 889.35 (w), 864.08 (m), 853.96 (m), 838.79 (s), 789.92 (m), 725.65 (m), 618.27 (w), 572.41 (w), 513.03 (w), 466.30 (w), 409.06 (w).

**MS (HR-CI^+^)** (m/z (%)) calculated for [C_37_H_40_N_2_In_1_Br_1_]^+^: 706.14136; found: 706.14085 (16.59), calculated for [C_37_H_41_N_2_In_1_Br_1_]^+^: 707.14919; found: 707.14035 (16.32).

**SS-UV/Vis** (BaSO_4_): *λ_max,ss_* = 510 nm.

**UV/Vis** (toluene): *λ_max_* = 506 nm, *ε_max_* (506 nm) = 0.91∙10^5^ L mol^-1^cm^-1^.

**PL** (toluene, *λ_Ex_* = 405 nm): *λ_F_* = 571 nm; *φ*_F_ = 6.1%.

Figure S132: ^1^H NMR spectrum of compound 5_MeBr_ in C_6_D_6_ at 300 K.

Figure S´133: ^13^C NMR spectrum of compound 5_MeBr_ in C_6_D_6_ at 300 K.


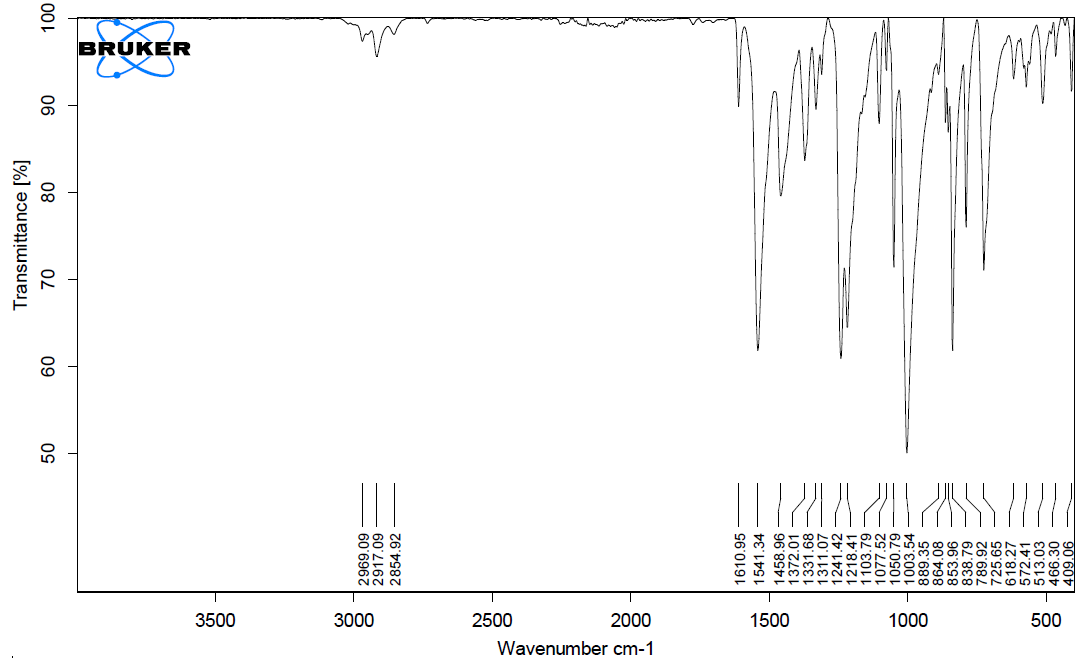


FigureS134: IR spectrum of compound 5_MeBr_.

Figure S135: Solid-state UV/VIS spectrum of compound 5_MeBr_.

Figure S136: Solution-state UV/VIS spectrum of compound 5_MeBr_ (2 µM in toluene).

Figure S137: Solution-state PL spectrum of compound 5_MeBr_ in toluene. Excitation with 405 nm continuous wave diode laser.

# 6. Optical properties

## 6.1 UV/Vis and photoluminescence spectra in toluene solution resp. solid state:

Figure S138: Normalized solid state UV/Vis spectra (left) and UV/Vis absorption spectra from toluene solution (right) of synthesized ^Mes^DPM triel dihalides (color scheme: violet = 2_Cl2_; brown = 2_Br2_; black = 3_Cl2_; red = 3_Br2_; green = 3_I2_; blue ‍= ‍4_Cl2_; light blue = 4_Br2_; pink = 4_I2_; yellow = 5_Cl2_; gold = 5_Br2_; dark blue = 5_I2_).

Figure S139: Normalized and stacked solid state UV/Vis spectra of synthesized ^Mes^DPM triel dihalides (color scheme: violet ‍= 2_Cl2_; brown = 2_Br2_; black = 3_Cl2_; red = 3_Br2_; green = 3_I2_; blue ‍= ‍4_Cl2_; light blue = 4_Br2_; pink = 4_I2_; yellow = 5_Cl2_; gold ‍= 5_Br2_; dark blue = 5_I2_).

Figure S140: Photoluminescence spectra of synthesized ^Mes^DPM triel dihalides (left = magnified view for 2_Br2_, 3_Cl2_ and 5_Cl2_; color scheme: violet = 2_Cl2_; brown = 2_Br2_; black = 3_Cl2_; red = 3_Br2_; green = 3_I2_; blue = 4_Cl2_; light blue = 4_Br2_; pink = 4_I2_; yellow = 5_Cl2_; gold = 5_Br2_; dark blue = 5_I2_). Excitation with 405 nm continuous wave diode laser.


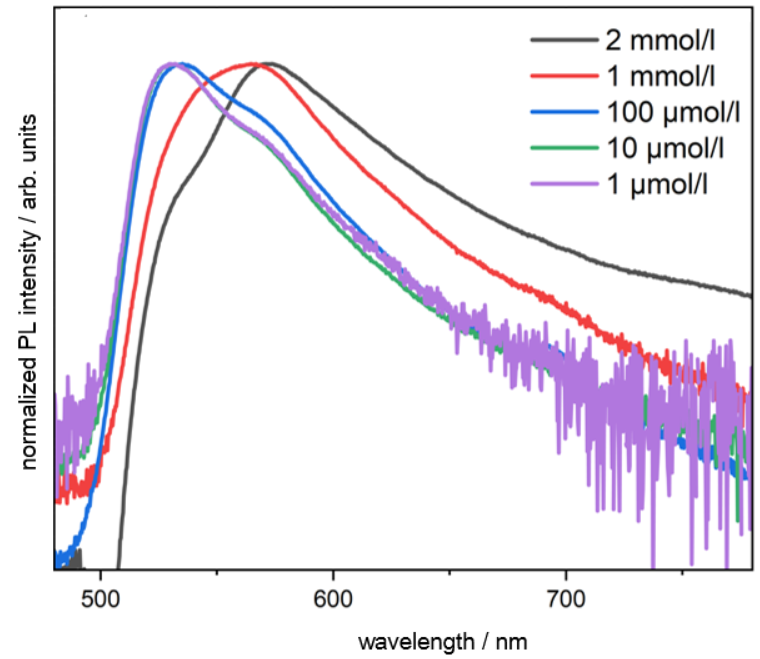


Figure S141: Normalized photoluminescence intensity of [(^Mes^DPM)BBr_2_] (2_Br2_) solutions in toluene (color scheme: black = 2 mmol, red = 1 mmol, blue = 100 µM, green = 10 µM, purple = 1 µM). Excitation: frequency doubled Ti:Sapphire Laser (400 nm, 78 MHz, 100 fs).

Figure S142: Normalized and stacked photoluminescence spectra of synthesized ^Mes^DPM triel dihalides (color scheme: violet = 2_Cl2_; brown = 2_Br2_; black = 3_Cl2_; red = 3_Br2_; green = 3_I2_; blue = 4_Cl2_; light blue = 4_Br2_; pink = 4_I2_; yellow = 5_Cl2_; gold ‍= 5_Br2_; dark blue = 5_I2_). Excitation with 405 nm continuous wave diode laser.


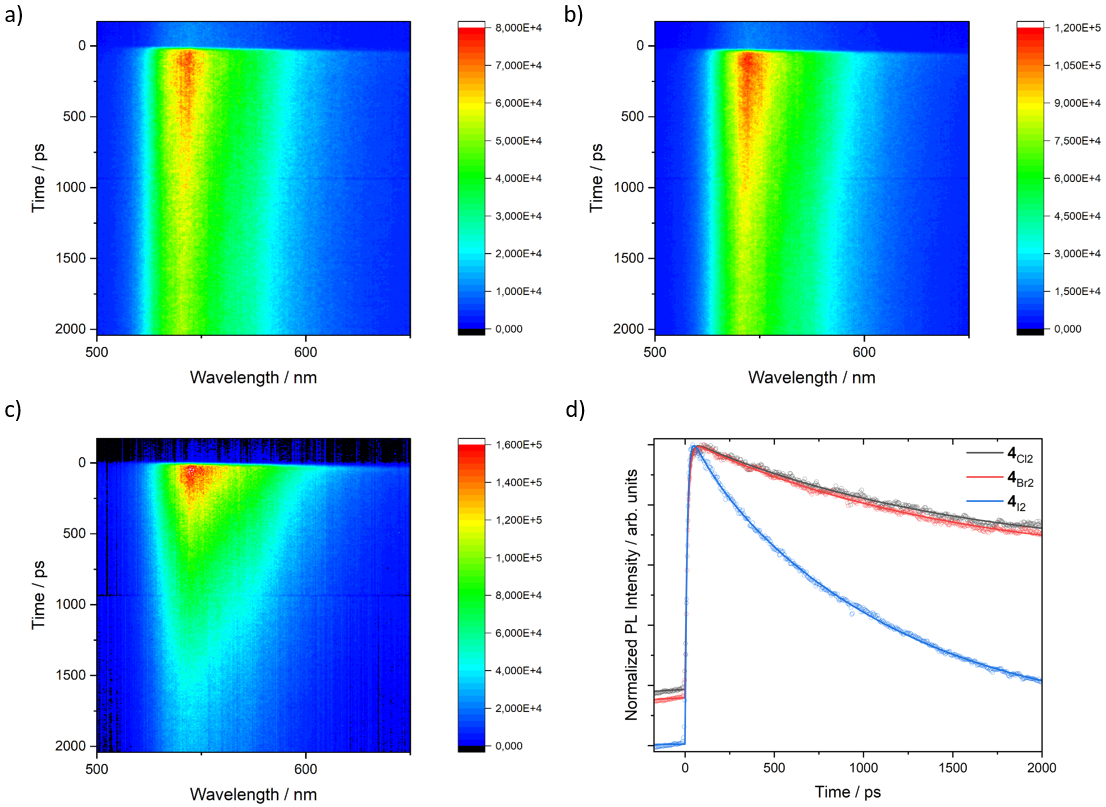


Figure S143: Time-resolved photoluminescence of toluene solutions of a) 4_Cl2_, b) 4_Br2_ and c) 4_I2_ as well as d) photoluminescence transient signals. Excitation: frequency doubled Ti:Sapphire Laser (400 nm, 78 MHz, 100 fs).

Figure S144: Normalized solid state UV/Vis spectra (left) and UV/Vis absorption spectra from toluene solution (right) of synthesized mixed substituted ^Mes^DPM triel alkyl halides (color scheme: black = 3_MeCl_; red = 3_MeBr_; green = 3_MeI_; blue ‍= ‍4_MeCl_; light blue = 4_MeBr_; pink = 4_MeI_; yellow = 5_MeCl_; gold = 5_MeBr_; dark blue = 5_MeI_).

Figure S145: Normalized and stacked solid state UV/Vis spectra of synthesized mixed substituted ^Mes^DPM triel alkyl halides (color scheme: black = 3_MeCl_; red = 3_MeBr_; green = 3_MeI_; blue ‍= ‍4_MeCl_; light blue = 4_MeBr_; pink = 4_MeI_; yellow = 5_MeCl_; gold ‍= ‍5_MeBr_; dark blue = 5_MeI_).

Figure S146: Photoluminescence spectra of synthesized mixed substituted ^Mes^DPM triel alkyl halides (color scheme: black ‍= 3_MeCl_; red = 3_MeBr_; green = 3_MeI_; blue = 4_MeCl_; light blue = 4_MeBr_; pink = 4_MeI_; yellow = 5_MeCl_; gold = 5_MeBr_; dark ‍blue ‍= ‍5_MeI_). Excitation with 405 nm continuous wave diode laser.

Figure S147: Normalized and stacked photoluminescence spectra of synthesized mixed substituted ^Mes^DPM triel alkyl halides (color scheme: black ‍= 3_MeCl_; red = 3_MeBr_; green = 3_MeI_; blue = 4_MeCl_; light blue = 4_MeBr_; pink = 4_MeI_; yellow ‍= ‍5_MeCl_; gold = 5_MeBr_; dark ‍blue ‍= ‍5_MeI_). Excitation with 405 nm continuous wave diode laser.


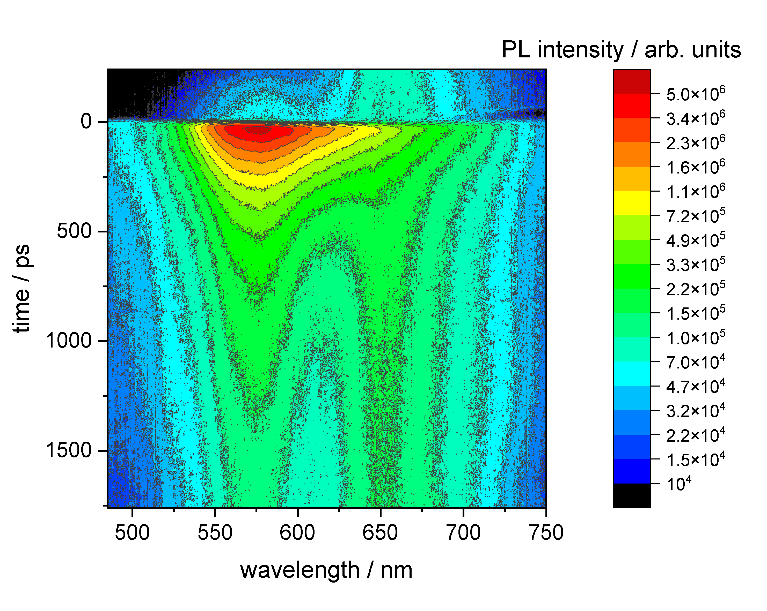

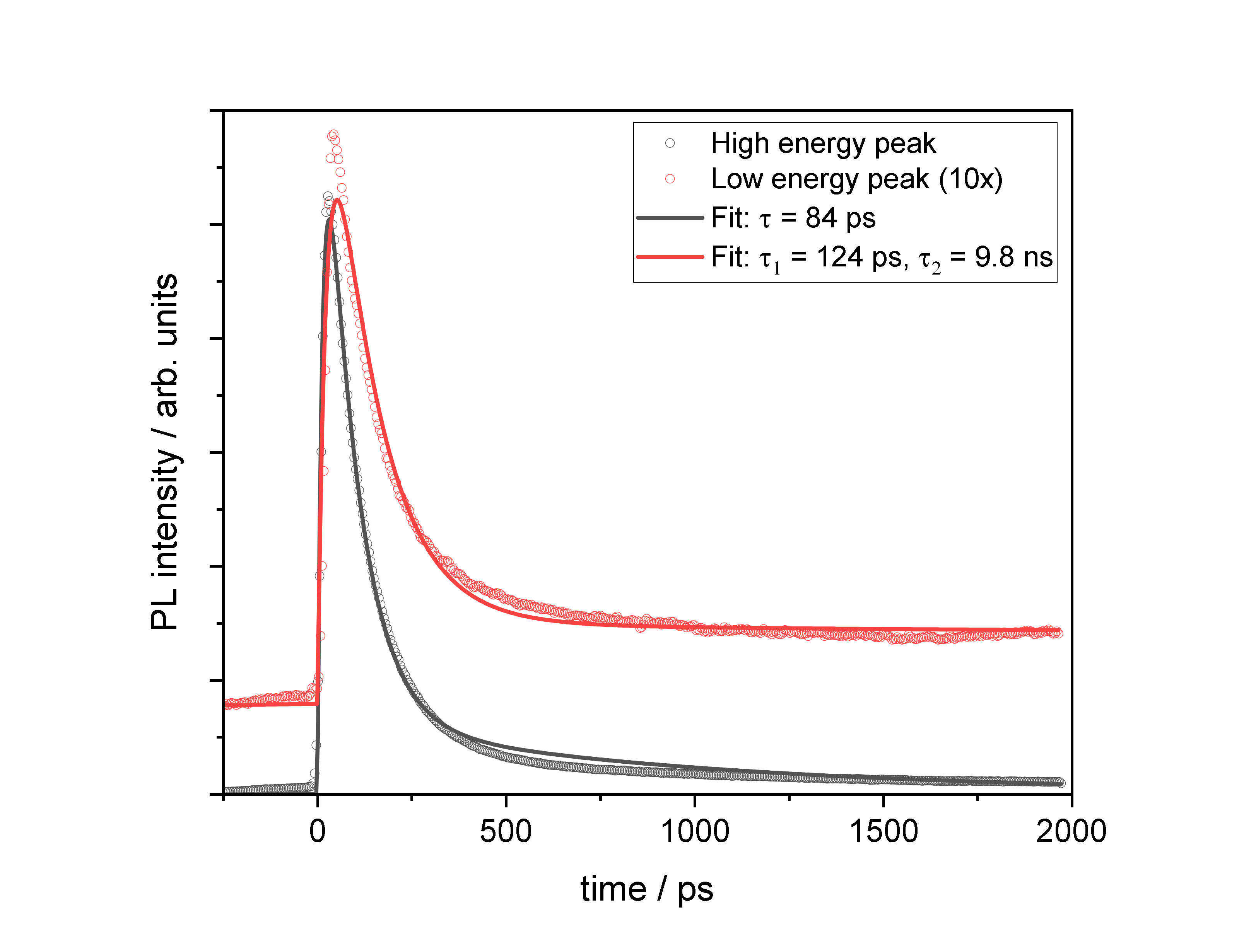


Figure S148: Time-resolved photoluminescence (left) and photoluminescence transient signals of the two main signals (right) of a 2 mM 2_Br2_ toluene solution. Excitation: frequency doubled Ti:Sapphire Laser (400 nm, 78 MHz, 100 fs). The high energy peak was integrated from 560 until 585 nm and the lower energy peak from 635 until 680 nm. The lower energy signal is magnified tenfold. Both were fitted using exponential model with the given lifetimes.

## 6.2 Spectroscopic data

Table S1: Overview on UV/Vis and photoluminescence data for synthesized ^Mes^DPM triel compounds.

| Compound | *λ*_max,ss_ /  nm | *λ*_max_ /  nm | *ε*_max_ (λ_max_)/  L mol^-1^cm^-1^ | *λ*_F_ /  nm | *φ*_F_ /  % |  |
| --- | --- | --- | --- | --- | --- | --- |
| [LBCl_2_] (**2**_Cl2_) | 528 | 524 | 0.69∙10^5^ | 572 | 41.5 |  |
| [LBBr_2_] (**2**_Br2_) | 532 | 529 | 0.48∙10^5^ | 590, 649 | 0.97 |  |
| [LAlCl_2_] (**3**_Cl2_) | 519 | 510 | 1.20∙10^5^ | 594, 657 | 0.6 |  |
| [LAlBr_2_] (**3**_Br2_) | 517 | 511 | 0.89∙10^5^ | 650 | 0.3 |  |
| [LAlI_2_] (**3**_I2_) | 518 | 510 | 1.25∙10^5^ | 644 | 0.02 |  |
| [LGaCl_2_] (**4**_Cl2_) | 512 | 508 | 1.31∙10^5^ | 560 | 19 |  |
| [LGaBr_2_] (**4**_Br2_) | 510 | 509 | 1.00∙10^5^ | 554 | 22 |  |
| [LGaI_2_] (**4**_I2_) | 517 | 512 | 1.02∙10^5^ | 566 | 14 |  |
| [LInCl_2_] (**5**_Cl2_) | 509 | 505 | 1.47∙10^5^ | 613, 655 | 0.4 |  |
| [LInBr_2_] (**5**_Br2_) | 507 | 505 | 1.02∙10^5^ | 623 | 0.3 |  |
| [LInI_2_] (**5**_I2_) | 513 | 508 | 0.58∙10^5^ | 622 | 0.2 |  |
| [LAl(Me)Cl] (**3**_MeCl_) | 516 | 509 | 1.06∙10^5^ | 587 | 0.6 |  |
| [LGa(Me)Cl] (**4**_MeCl_) | 506 | 507 | 1.72∙10^5^ | 554 | 39 |  |
| [LIn(Me)Cl] (**5**_MeCl_) | 513 | 504 | 1.17∙10^5^ | 592 | 0.9 |  |
| [LAl(Me)Br] (**3**_MeBr_) | 518 | 511 | 1.21∙10^5^ | 553 | 29.0 |  |
| [LGa(Me)Br] (**4**_MeBr_) | 511 | 510 | 0.95∙10^5^ | 553 | 23.8 |  |
| [LIn(Me)Br] (**5**_MeBr_) | 510 | 506 | 0.91∙10^5^ | 571 | 6.1 |  |
| [LAl(Me)I] (**3**_MeI_) | 524 | 514 | 1.05∙10^5^ | 619 | 0.2 |  |
| [LGa(Me)I] (**4**_MeI_) | 521 | 514 | 1.08∙10^5^ | 565 | 0.5 |  |
| [LIn(Me)I] (**5**_MeI_) | 512 | 508 | 1.03∙10^5^ | 560 | 0.6 |  |
| L= ^Mes^DPM, *λ*_max,ss_ = absorption maximum (solid state), *λ*_max_ = absorption maximum (toluene solution), *ε*_max_ (*λ*_max_) = molar absorption coefficient (at *λ*_max_), *λ*_F_ = fluorescence maximum, *φ*_F_ = fluorescence quantum yield. | | | | | | |

# 7. Crystallographic data:

## 7.1 [(^Mes^DPM)Li∙thf] (**1∙THF**)


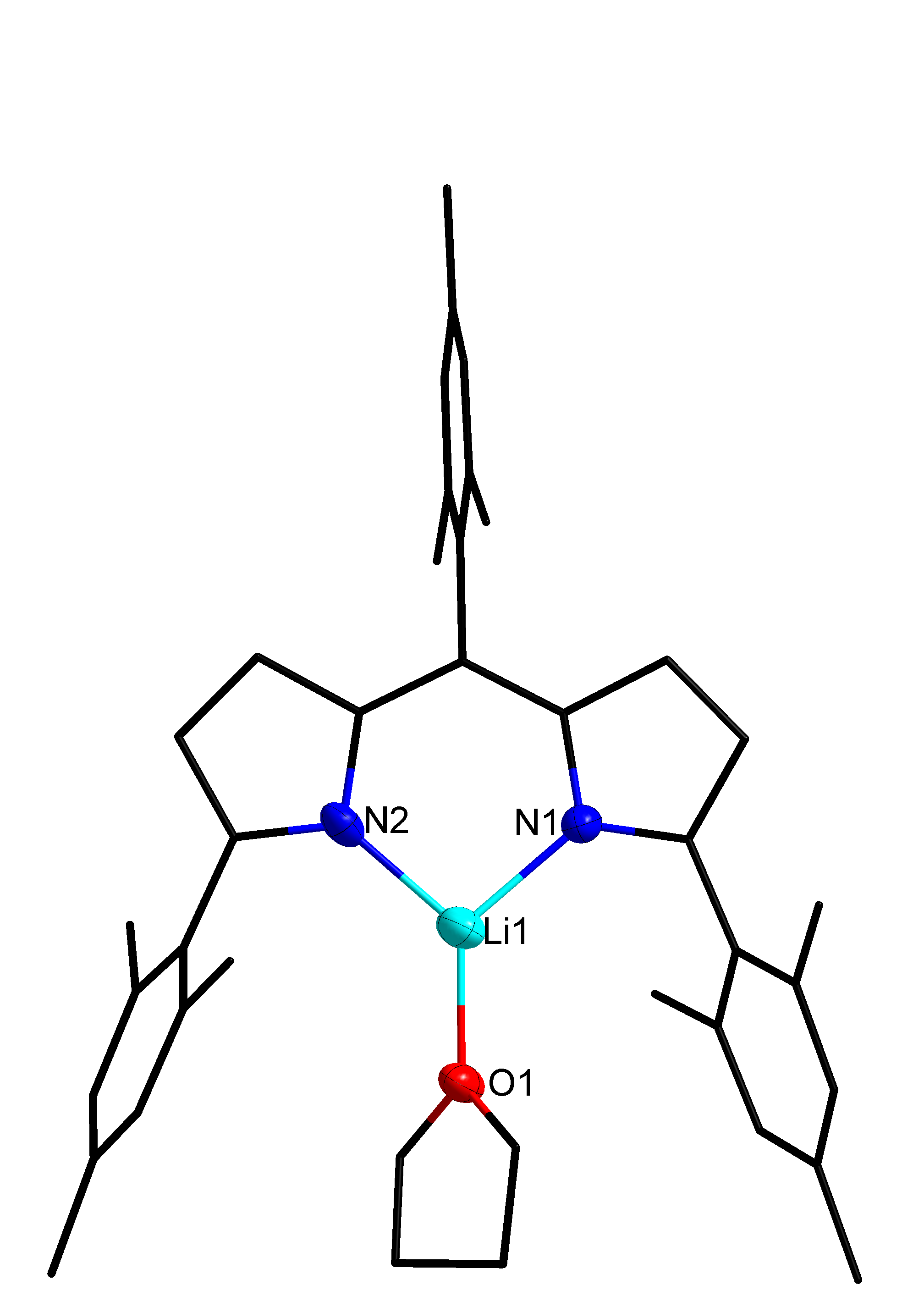


Figure S149: Solid state molecular structure for [(^Mes^DPM)Li∙thf] (6∙thf) with thermal ellipsoids set at the 50% probability level (color scheme: C = black, Li = light blue, O = red, N = blue). Carbon atoms are depicted as wireframe, carbon-bound hydrogen atoms are omitted for clarity.

Table S2: Crystal data and structure refinement for [(^Mes^DPM)Li∙thf] (6∙thf).

| **Compound** | **6∙thf** |
| --- | --- |
| Empirical formula | C_40_H_45_LiN_2_O |
| Formular weight | 576.72 |
| Temperature / K | 100 |
| Crystal system | Monoclinic |
| Space group | *P*2_1_/*c* |
| a / Å | 7.863(3) |
| b / Å | 12.763(5) |
| c / Å | 33.757(14) |
| α / ° | 90 |
| β / ° | 91.720(6) |
| γ / ° | 90 |
| Volume / Å^3^ | 3386(2) |
| Z | 4 |
| Density (calculated) / g/cm^3^ | 1.131 |
| Absorption coefficient / mm^-1^ | 0.066 |
| F(000) | 1240.0 |
| Crystal size / mm | 0.300 × 0.300 × 0.200 |
| Radiation / nm | MoKα (λ = 0.71073) |
| 2Θ range for data collection / ° | 2.414 to 49.746 |
| Index ranges | -9 ≤ h ≤ 4, -14 ≤ k ≤ 14, -39 ≤ l ≤ 39 |
| Reflections collected | 16387 |
| Independent reflections | 5692 [*R*_int_ = 0.1965*, *R*_sigma_ = 0.2555] |
| Data/restraints/parameters | 5692/0/407 |
| Goodness-of-fit on F^2^ (GooF) | 0.964 |
| Final R indexes [I>=2σ (I)] | *R*_1_ = 0.0930, *wR*_2_ = 0.1976 |
| Final R indexes [all data] | *R*_1_ = 0.2415, *wR*_2_ = 0.2637 |
| Largest diff. Peak/hole / e Å^-3^ | 0.32/-0.30 |
| ccdc | 2413317 |

*high *R_int_* due to bad crystal quality and size as well as weak reflection intensities due to missing heavy elements

## 7.2 [(^Mes^DPM)BMe_2_] (**2_Me2_**)


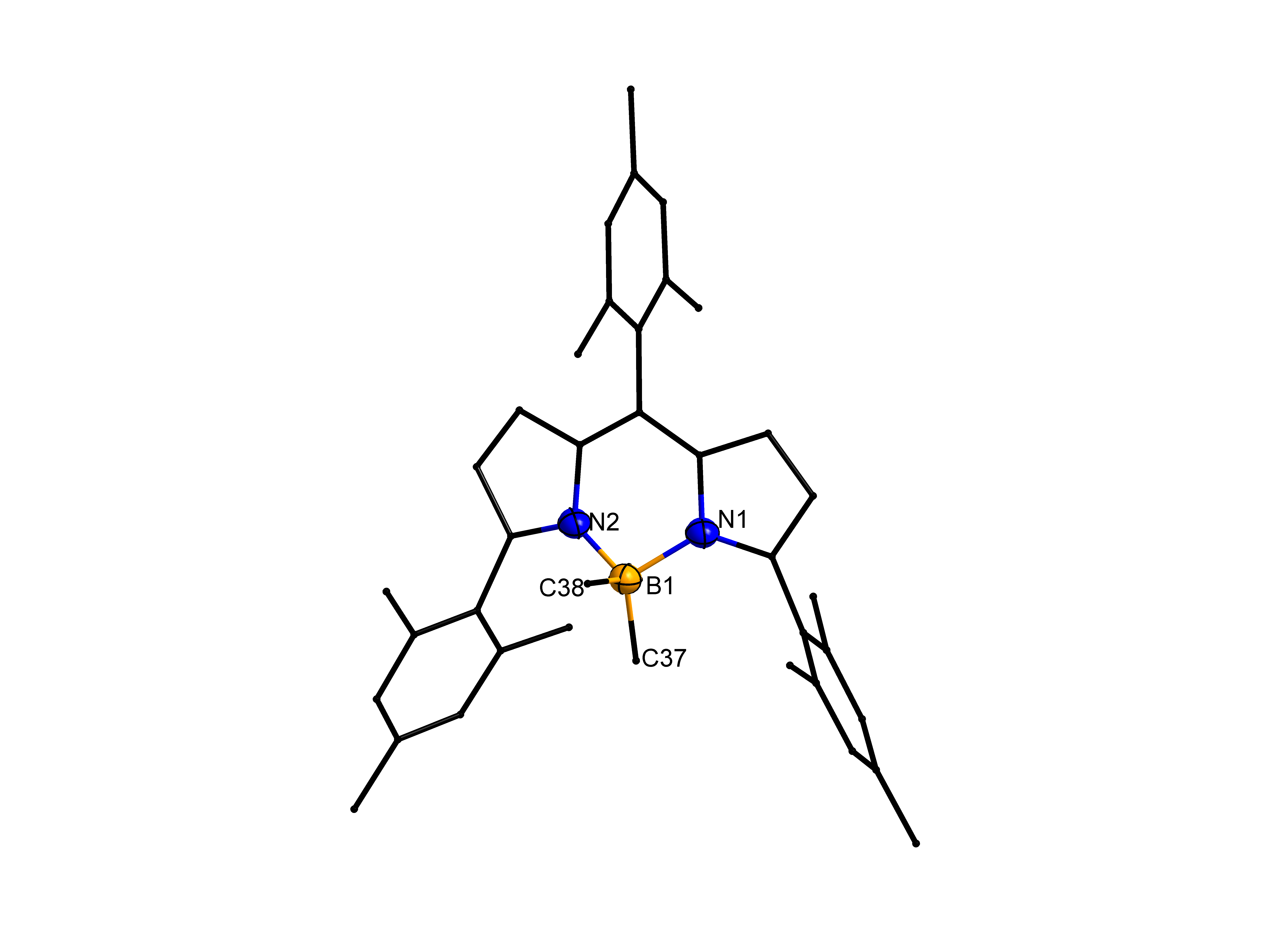


Figure S150: Solid state molecular structure of [(^Mes^DPM)BMe_2_] (2_Me2_) with thermal ellipsoids set at the 50% probability level (color scheme: C = black, N = blue, B = light orange). Carbon atoms are depicted as wireframe, carbon-bound hydrogen atoms are omitted for clarity.

Table S3: Crystal data and structure refinement for [(^Mes^DPM)BMe_2_] (2_Me2_).

| **Compound** | **2_Me2_** |
| --- | --- |
| Empirical formula | C_38_H_43_B_1_N_2_ |
| Formular weight | 538.55 |
| Temperature / K | 100 |
| Crystal system | triclinic |
| Space group | *P*$\bar{1}$ |
| a / Å | 8.1763(3) |
| b / Å | 13.7263(5) |
| c / Å | 14.8596(5) |
| α / ° | 77.378(3) |
| β / ° | 86.978(3) |
| γ / ° | 73.744(3) |
| Volume / Å^3^ | 1562.27(10) |
| Z | 2 |
| Density (calculated) / g/cm^3^ | 1.145 |
| Absorption koefficient / mm^-1^ | 0.491 |
| F(000) | 580.0 |
| Crystal size resp. radius/ mm | 0.0562 |
| Radiation / nm | CuKα (λ = 1.54186) |
| 2Θ range for data collection / ° | 6.096 to 130.18 |
| Index ranges | -9 ≤ h ≤ 9, -16 ≤ k ≤ 11, -17 ≤ l ≤ 17 |
| Reflections collected | 31418 |
| Independent reflections | 5300 [*R*_int_ = 0.0989, *R*_sigma_ = 0.0826] |
| Data/restraints/parameters | 5300/0/381 |
| Goodness-of-fit on F^2^ (GooF) | 0.867 |
| Final R indexes [I>=2σ (I)] | *R*_1_ = 0.0529, *wR*_2_ = 0.1160 |
| Final R indexes [all data] | *R*_1_ = 0.0993, *wR*_2_ = 0.1322 |
| Largest diff. Peak/hole / e Å^-3^ | 0.18/-0.25 |
| ccdc | 2413333 |

## 7.3 [(^Mes^DPM)MCl_2_] (M = B (**2_Cl2_**), Al (**3_Cl2_**), Ga (**4_Cl2_**), In (**5_Cl2_**))

a) b) c) d)


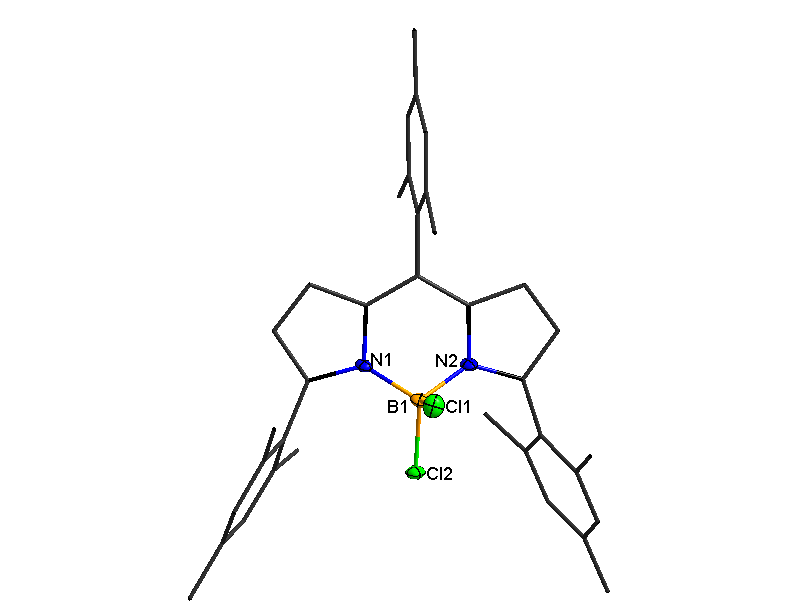

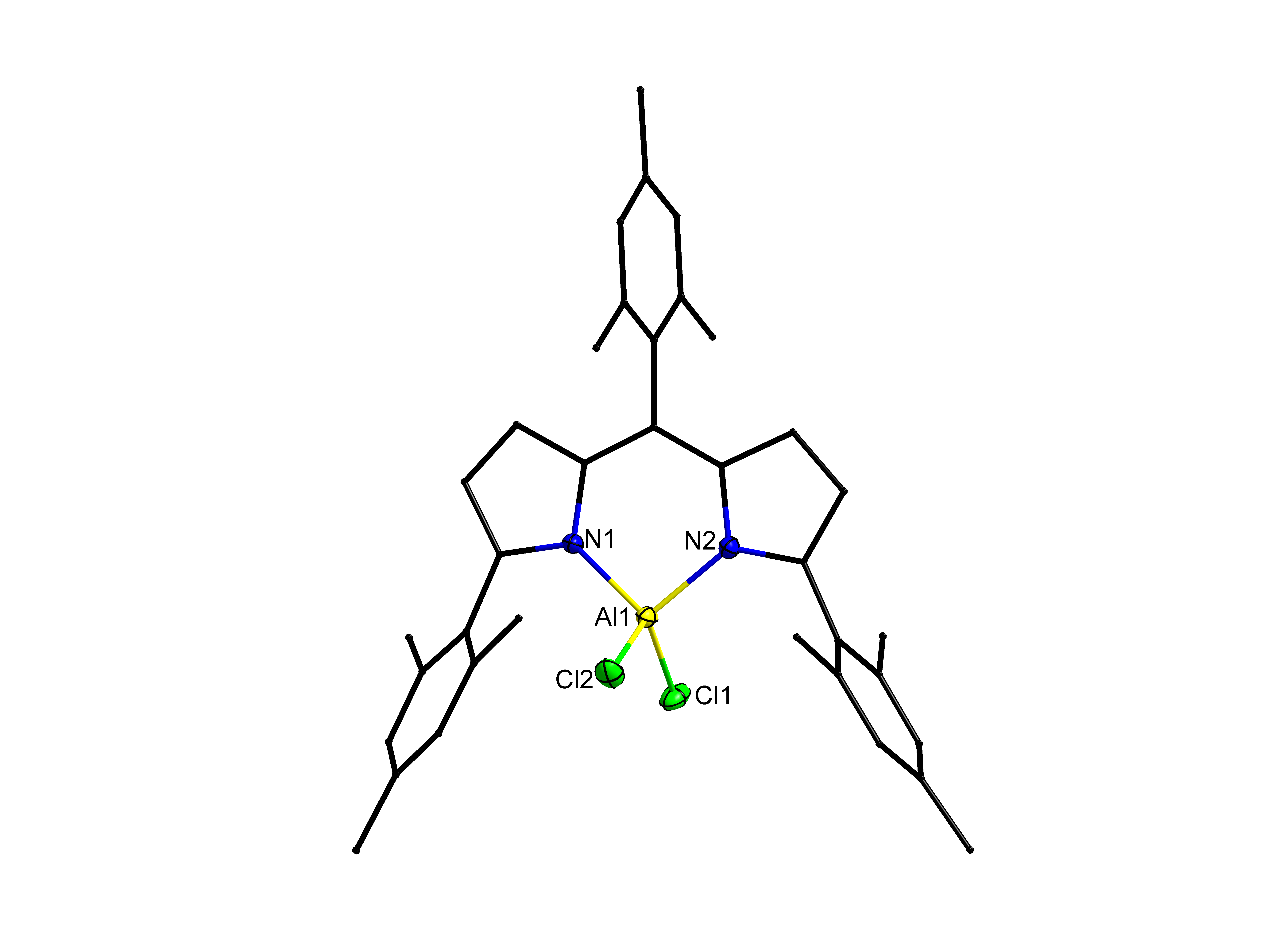

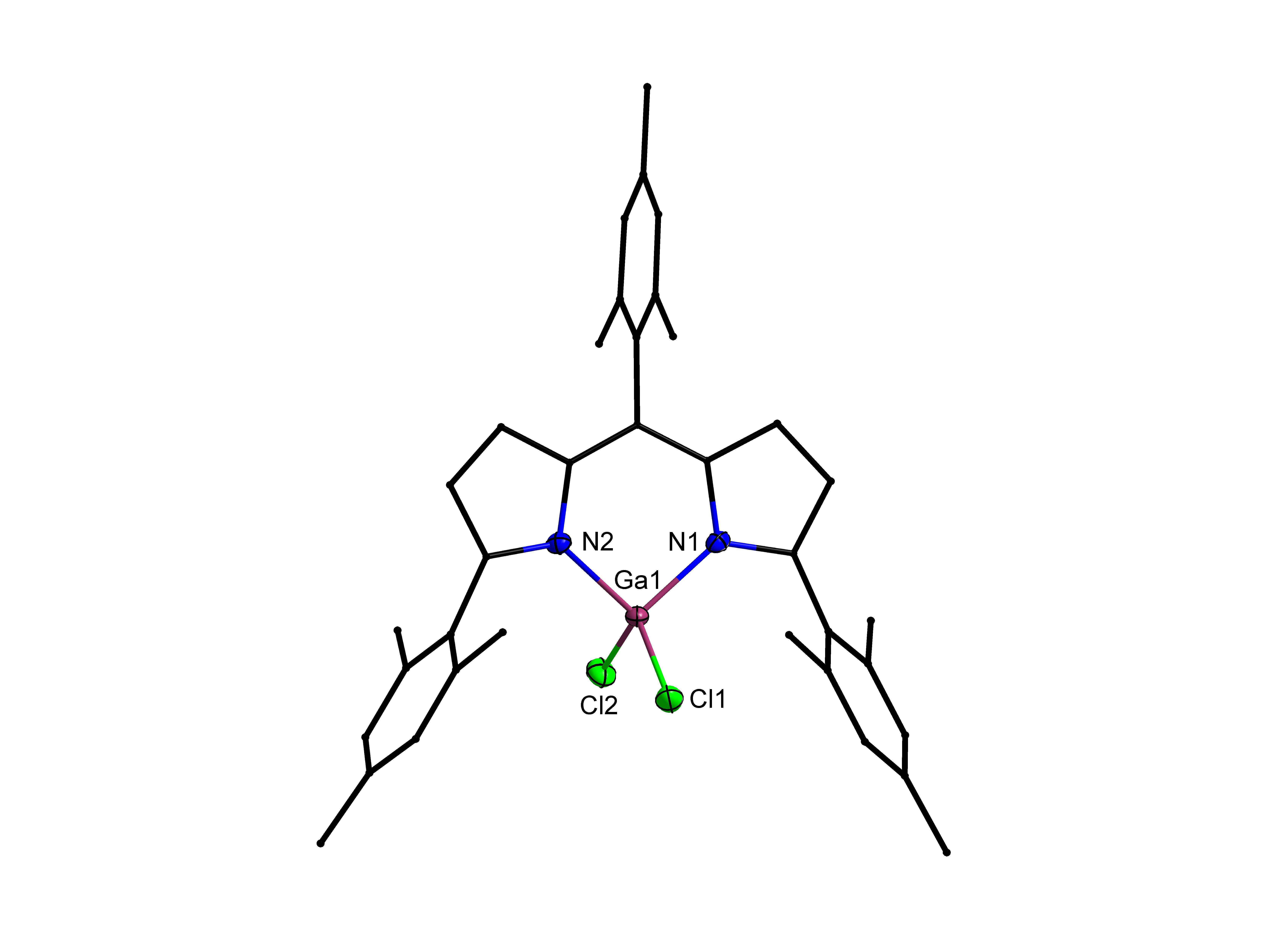

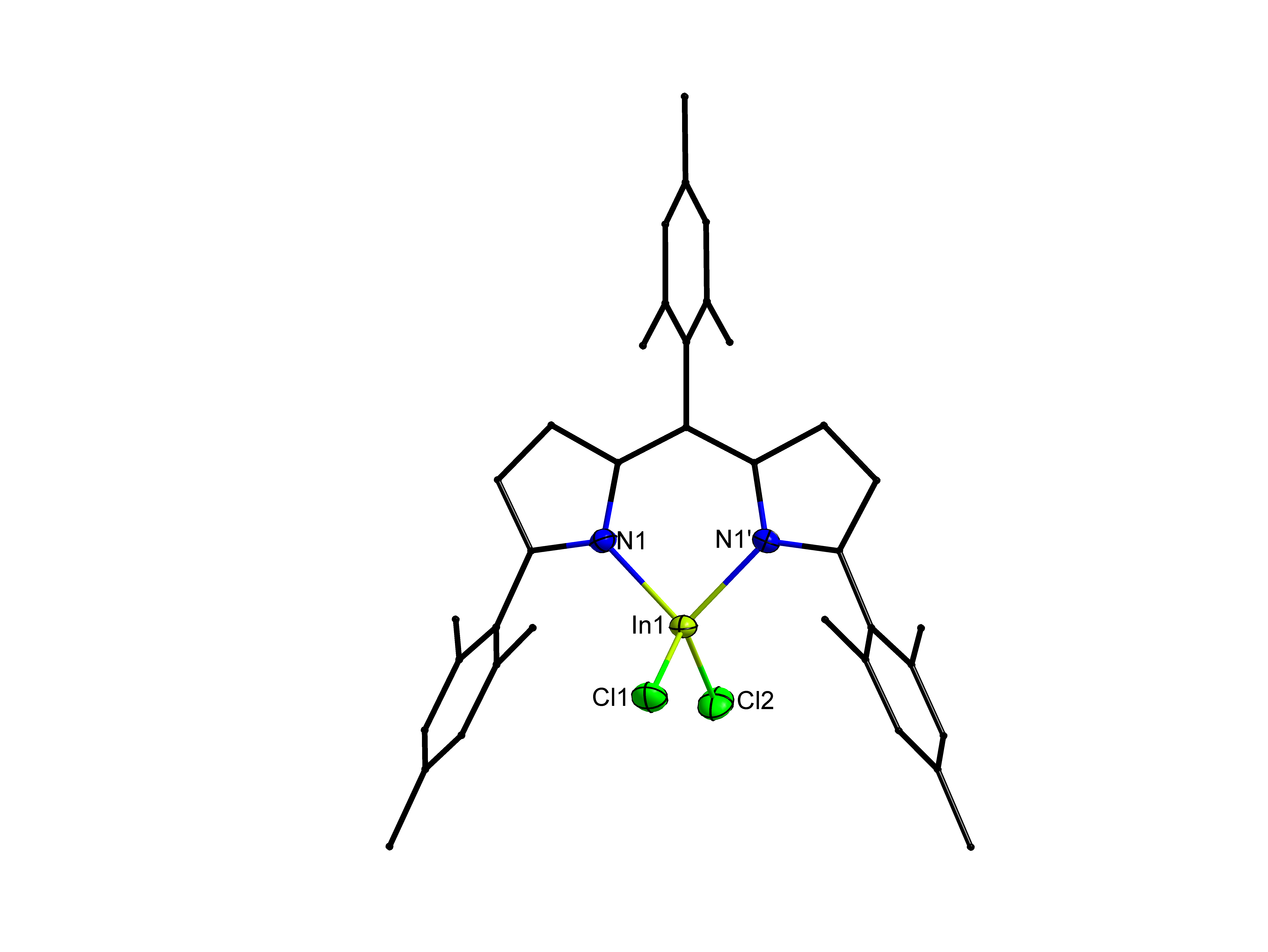


Figure S151: Solid state molecular structures for a) [(^Mes^DPM)BCl_2_] (2_Cl2_), b) [(^Mes^DPM)AlCl_2_] (3_Cl2_), c) [(^Mes^DPM)GaCl_2_] (4_Cl2_) and d) [(^Mes^DPM)InCl_2_] (5_Cl2_) with thermal ellipsoids set at the 50% probability level (color scheme: C = black, N = blue, B = light orange, Al = yellow, Ga = violet, In = light green, Cl = green).

Table S4: Crystal data and structure refinements for [(^Mes^DPM)BCl_2_] (2_Cl2_), [(^Mes^DPM)AlCl_2_] (3_Cl2_), [(^Mes^DPM)GaCl_2_] (4_Cl2_) and [(^Mes^DPM)InCl_2_] (5_Cl2_).

| **Compound** | **2_Cl2_** | **3_Cl2_** | **4_Cl2_** | **5_Cl2_** |
| --- | --- | --- | --- | --- |
| Empirical formula | C_36_H_37_B_1_Cl_2_N_2_ | C_36_H_37_Al_1_Cl_2_N_2_ | C_36_H_37_Ga_1_Cl_2_N_2_ | C_36_H_37_In_1_Cl_2_N_2_ |
| Formular weight | 579.38 | 595.55 | 638.29 | 683.39 |
| Temperature / K | 100.0 | 100.0 | 100.0 | 100.0 |
| Crystal system | triclinic | monoclinic | monoclinic | monoclinic |
| Space group | *P*$\bar{1}$ | *P*2_1_ | *P*2_1_ | *P*2_1_/*m* |
| a / Å | 8.173(4) | 8.0342(3) | 8.0103(8) | 7.96570(10) |
| b / Å | 13.623(5) | 14.2578(6) | 14.2674(13) | 14.3480(2) |
| c / Å | 14.700(6) | 14.0670(6) | 14.0960(12) | 14.6519(2) |
| α / ° | 77.67(2) | 90 | 90 | 90 |
| β / ° | 87.26(2) | 96.8830(10) | 97.044(3) | 103.4720(10) |
| γ / ° | 73.31(2) | 90 | 90 | 90 |
| Volume / Å^3^ | 1531.3(11) | 1599.76(11) | 1598.8(3) | 1628.51(4) |
| Z | 2 | 2 | 2 | 2 |
| Density (calculated) / g/cm^3^ | 1.257 | 1.236 | 1.326 | 1.394 |
| Absorption coefficient / mm^-1^ | 0.240 | 0.258 | 1.055 | 7.502 |
| F(000) | 612.0 | 628.0 | 664.0 | 700.0 |
| Crystal size resp. radius / mm | 0.3 × 0.2 × 0.15 | 0.162 × 0.154 × 0.121 | 0.118 × 0.101 × 0.045 | 0.1003 |
| Radiation / nm | MoKα (λ = 0.71073) | MoKα (λ = 0.71073) | MoKα (λ = 0.71073) | CuKα (λ = 1.54186) |
| 2Θ range for data collection / ° | 2.836 to 53.222 | 4.082 to 61.626 | 5.124 to 51.996 | 6.204 to 152.096 |
| Index ranges | -8 ≤ h ≤ 10, -17 ≤ k ≤ 16, -18 ≤ l ≤ 18 | -11 ≤ h ≤ 11, -20 ≤ k ≤ 20, -20 ≤ l ≤ 19 | -9 ≤ h ≤ 9, -17 ≤ k ≤ 17, -17 ≤ l ≤ 17 | -4 ≤ h ≤ 9, -18 ≤ k ≤ 17, -18 ≤ l ≤ 18 |
| Reflections collected | 13189 | 22571 | 37745 | 31182 |
| Independent reflections | 6271 [*R*_int_ = 0.0917, *R*_sigma_ = 0.1588] | 9759 [*R*_int_ = 0.0422, *R*_sigma_ = 0.0702] | 6274 [*R*_int_ = 0.1011, *R*_sigma_ = 0.0747] | 3504 [*R*_int_ = 0.0428, *R*_sigma_ = 0.0188] |
| Data/restraints/parameters | 6271/0/385 | 9759/1/380 | 6274/1/380 | 3504/0/211 |
| Goodness-of-fit on F^2^ (GooF) | 0.988 | 1.059 | 1.064 | 1.126 |
| Final R indexes [I>=2σ (I)] | *R*_1_ = 0.0721,  *wR*_2_ = 0.1491 | *R*_1_ = 0.0483,  *wR*_2_ = 0.0922 | *R*_1_ = 0.0426,  *wR*_2_ = 0.0858 | *R*_1_ = 0.0266,  *wR*_2_ = 0.0695 |
| Final R indexes [all data] | *R*_1_ = 0.1497,  *wR*_2_ = 0.1837 | *R*_1_ = 0.0697,  *wR*_2_ = 0.0985 | *R*_1_ = 0.0555,  *wR*_2_ = 0.0889 | *R*_1_ = 0.0276,  *wR*_2_ = 0.0699 |
| Largest diff. Peak/hole / e Å^-3^ | 0.50/-0.49 | 0.42/-0.31 | 0.47/-0.55 | 0.40/-0.65 |
| Flack parameter | - | 0.20(6) | 0.084(15) | - |
| ccdc | 2413316 | 2413320 | 2413332 | 2413325 |

## 7.4 [(^Mes^DPM)MBr_2_] (M = B (**2_Br2_**), Al (**3_Br2_**), Ga (**4_Br2_**), In (**5_Br2_**))

a) b) c) d)


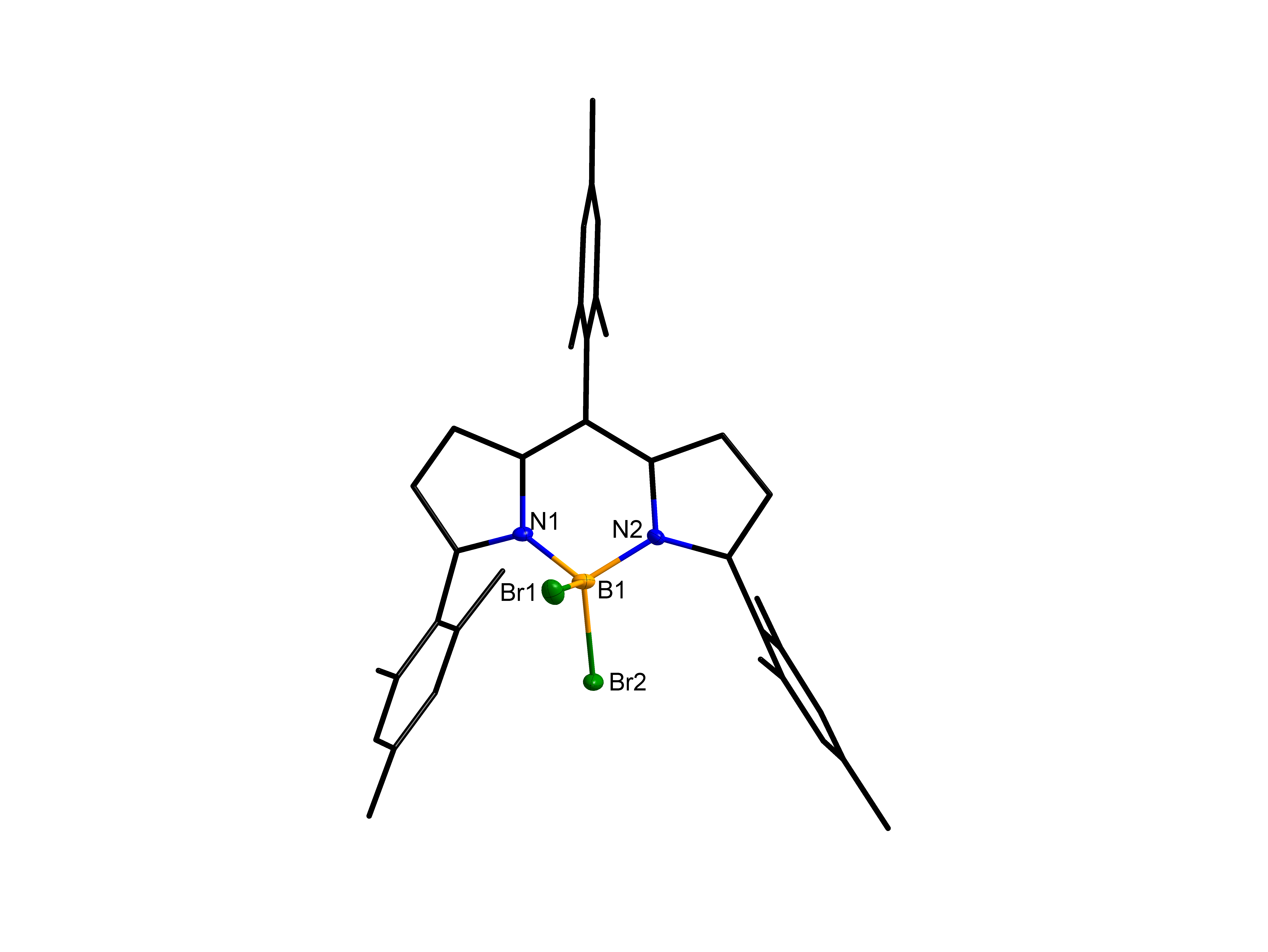

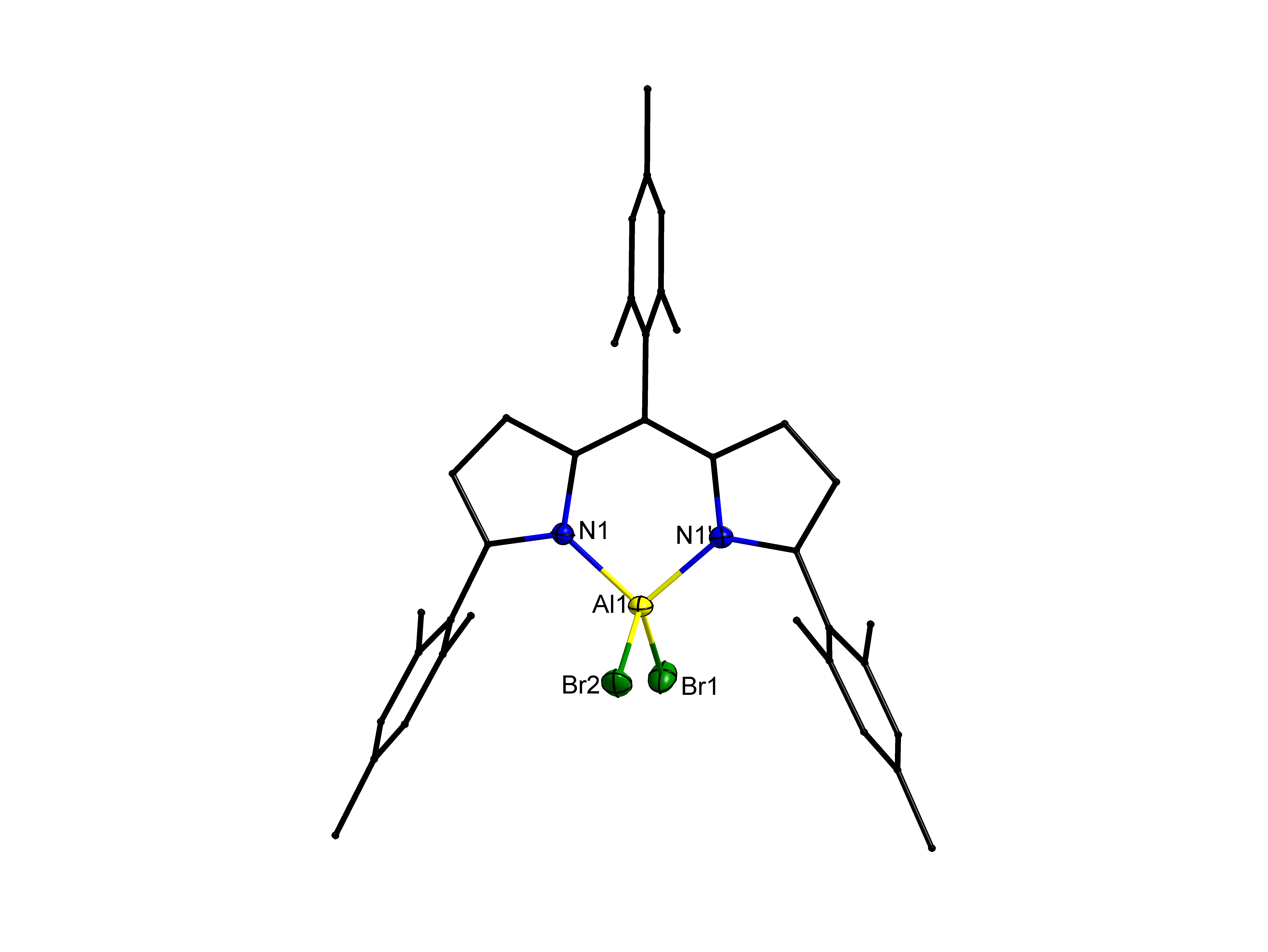

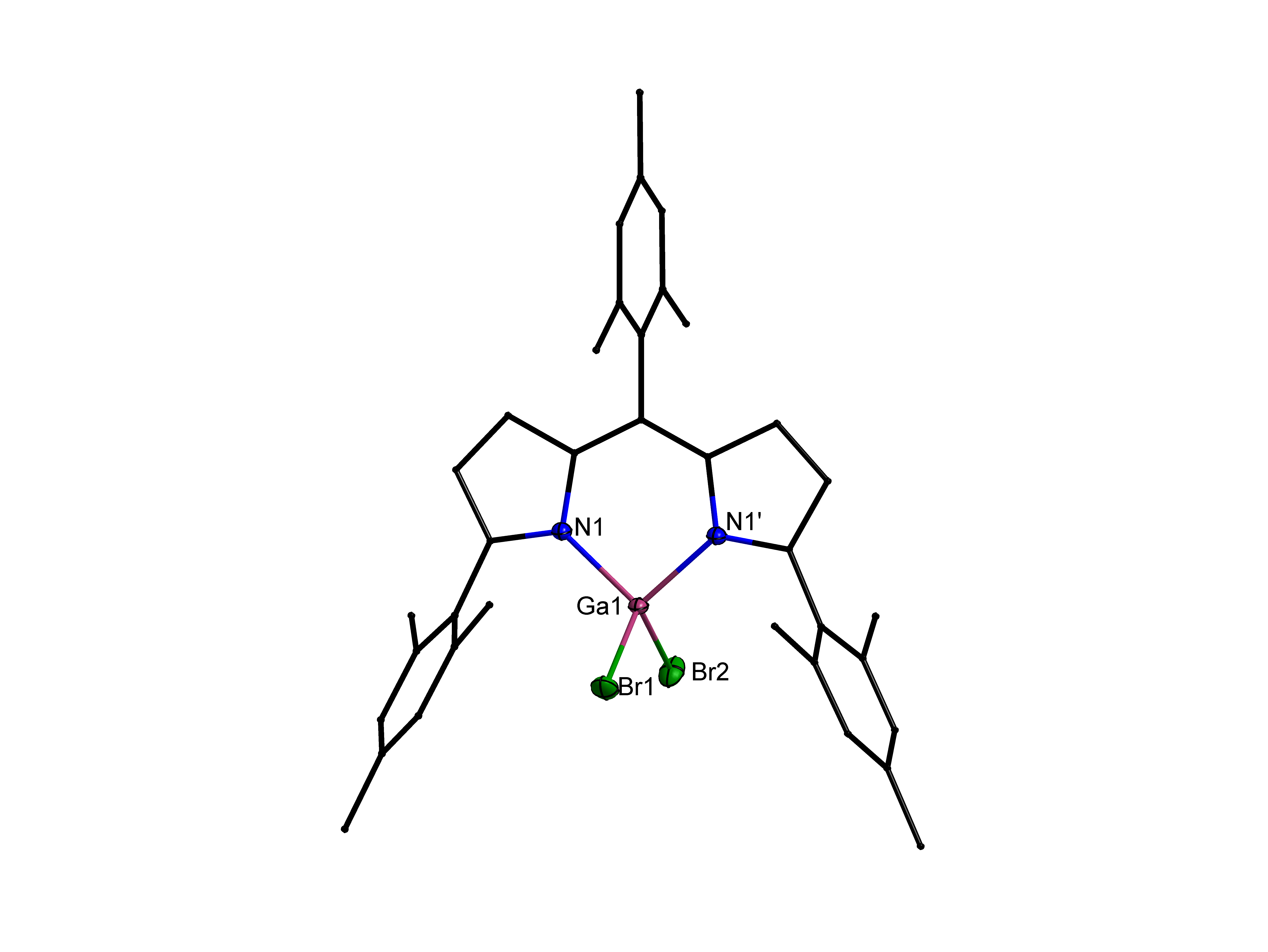

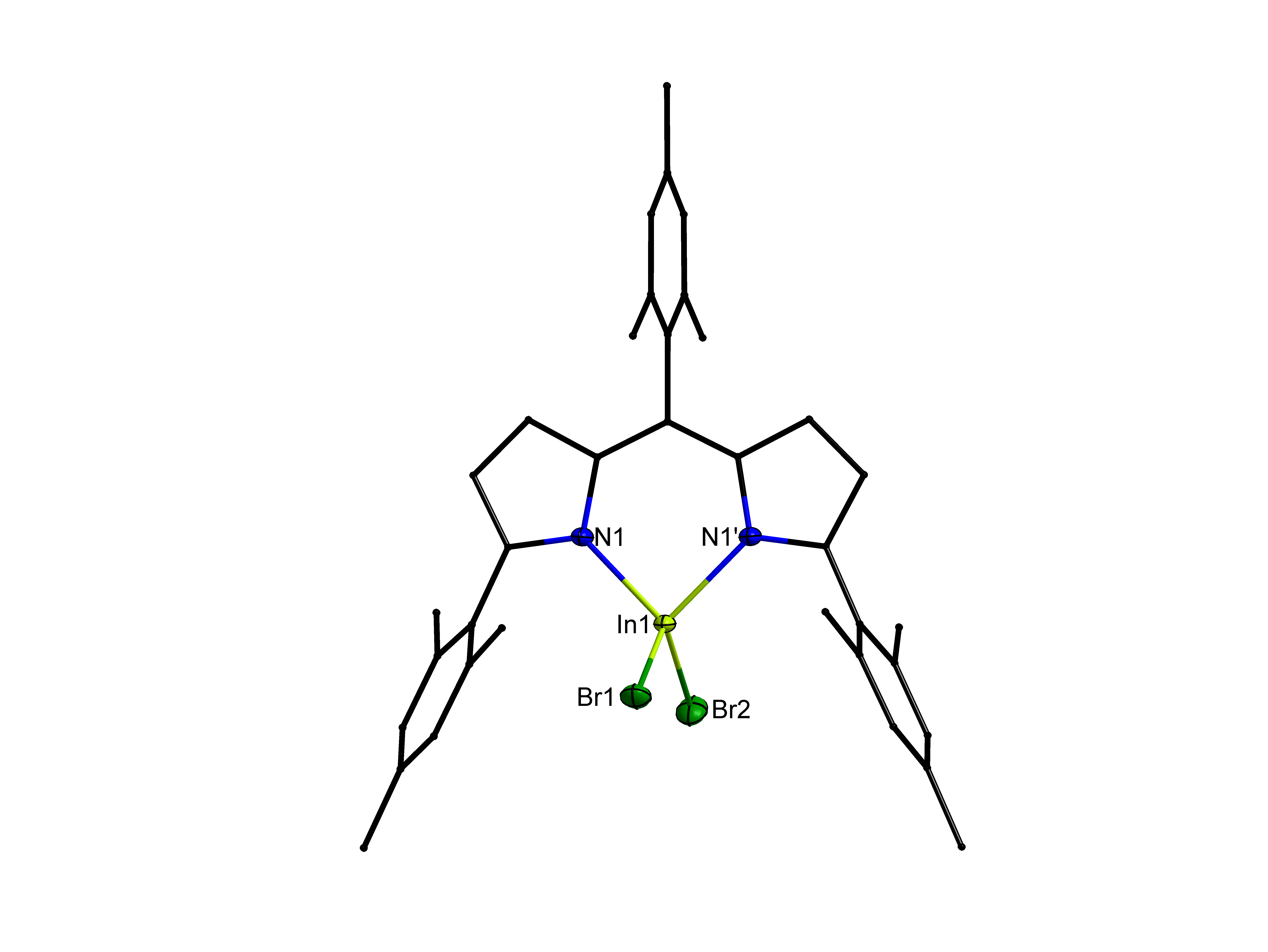


Figure S152: Solid state molecular structures for a) [(^Mes^DPM)BBr_2_] (2_Br2_), b) [(^Mes^DPM)AlBr_2_] (3_Br2_), c) [(^Mes^DPM)GaBr_2_] (4_Br2_) and d) [(^Mes^DPM)InBr_2_] (5_Br2_) with thermal ellipsoids set at the 50% probability level (color scheme: C = black, N = blue, B = light orange, Al = yellow, Ga = violet, In = light green, Br = dark green).

Table S5: Crystal data and structure refinements for [(^Mes^DPM)BBr_2_] (2_Br2_), [(^Mes^DPM)AlBr_2_] (3_Br2_), [(^Mes^DPM)GaBr_2_] (4_Br2_) and [(^Mes^DPM)InBr_2_] (5_Br2_).

| **Compound** | **2_Br2_** | **3_Br2_** | **4_Br2_** | **5_Br2_** |
| --- | --- | --- | --- | --- |
| Empirical formula | C_36_H_37_B_1_Br_2_N_2_ | C_36_H_37_Al_1_Br_2_N_2_ | C_36_H_37_Ga_1_Br_2_N_2_ | C_36_H_37_In_1_Br_2_N_2_ |
| Formular weight | 668.30 | 684.47 | 727.21 | 772.31 |
| Temperature / K | 100.42 | 100.0 | 100.0 | 100 |
| Crystal system | monoclinic | monoclinic | monoclinic | Monoclinic |
| Space group | *P*2_1_/*c* | *P*2_1_/*m* | *P*2_1_/*m* | *P*2_1_/*m* |
| a / Å | 13.728(4) | 8.0031(3) | 7.9867(3) | 7.9256(4) |
| b / Å | 8.577(3) | 14.3897(3) | 14.3895(6) | 14.3311(7) |
| c / Å | 26.994(9) | 14.5348(5) | 14.5513(5) | 14.7795(6) |
| α / ° | 90 | 90 | 90 | 90 |
| β / ° | 98.450(7) | 103.009(9) | 103.041(2) | 102.695(2) |
| γ / ° | 90 | 90 | 90 | 90 |
| Volume / Å^3^ | 3144.1(17) | 1630.90(9) | 1629.17(11) | 1637.66(13) |
| Z | 4 | 2 | 2 | 2 |
| Density (calculated) / g/cm^3^ | 1.412 | 1.394 | 1.482 | 1.566 |
| Absorption coefficient / mm^-1^ | 2.606 | 2.539 | 3.324 | 3.188 |
| F(000) | 1368.0 | 700.0 | 736.0 | 772.0 |
| Crystal size resp. radius / mm | 0.1 × 0.1 × 0.1 | 0.0719 | 0.391 × 0.364 × 0.29 | 0.1 × 0.087 × 0.055 |
| Radiation / nm | MoKα (λ = 0.71073) | MoKα (λ = 0.71073) | MoKα (λ = 0.71073) | MoKα (λ = 0.71073) |
| 2Θ range for data collection / ° | 3 to 52.216 | 5.224 to 63.53 | 4.034 to 68.784 | 5.268 to 61.024 |
| Index ranges | -16 ≤ h ≤ 16, -9 ≤ k ≤ 10, -33 ≤ l ≤ 32 | -11 ≤ h ≤ 10, -21 ≤ k ≤ 11, -21 ≤ l ≤ 21 | -12 ≤ h ≤ 12, -22 ≤ k ≤ 22, -23 ≤ l ≤ 23 | -11 ≤ h ≤ 11, -20 ≤ k ≤ 20, -21 ≤ l ≤ 21 |
| Reflections collected | 17006 | 23514 | 58609 | 55845 |
| Independent reflections | 6197 [*R*_int_ = 0.0595, *R*_sigma_ = 0.0766] | 5478 [*R*_int_ = 0.0306, *R*_sigma_ = 0.0502] | 6745 [*R*_int_ = 0.0393, *R*_sigma_ = 0.0316] | 5176 [*R*_int_ = 0.0551, *R*_sigma_ = 0.0320] |
| Data/restraints/parameters | 6197/0/379 | 5478/0/211 | 6745/0/211 | 5176/0/211 |
| Goodness-of-fit on F^2^ (GooF) | 0.982 | 1.014 | 1.078 | 1.083 |
| Final R indexes [I>=2σ (I)] | *R*_1_ = 0.0404,  *wR*_2_ = 0.0722 | *R*_1_ = 0.0367,  *wR*_2_ = 0.0773 | *R*_1_ = 0.0297,  *wR*_2_ = 0.0724 | *R*_1_ = 0.0282,  *wR*_2_ = 0.0553 |
| Final R indexes [all data] | *R*_1_ = 0.0715,  *wR*_2_ = 0.0801 | *R*_1_ = 0.0693,  *wR*_2_ = 0.0852 | *R*_1_ = 0.0454,  *wR*_2_ = 0.0759 | *R*_1_ = 0.0416,  *wR*_2_ = 0.0577 |
| Largest diff. Peak/hole / e Å^-3^ | 0.46/-0.51 | 0.60/-0.76 | 1.07/-0.78 | 0.61/-0.52 |
| ccdc | 2413318 | 2413322 | 2413329 | 2413331 |

## 7.5 [(^Mes^DPM)MI_2_] (M = Al (**3_I2_**), Ga (**4_I2_**), In (**5_I2_**))

a) b) c)


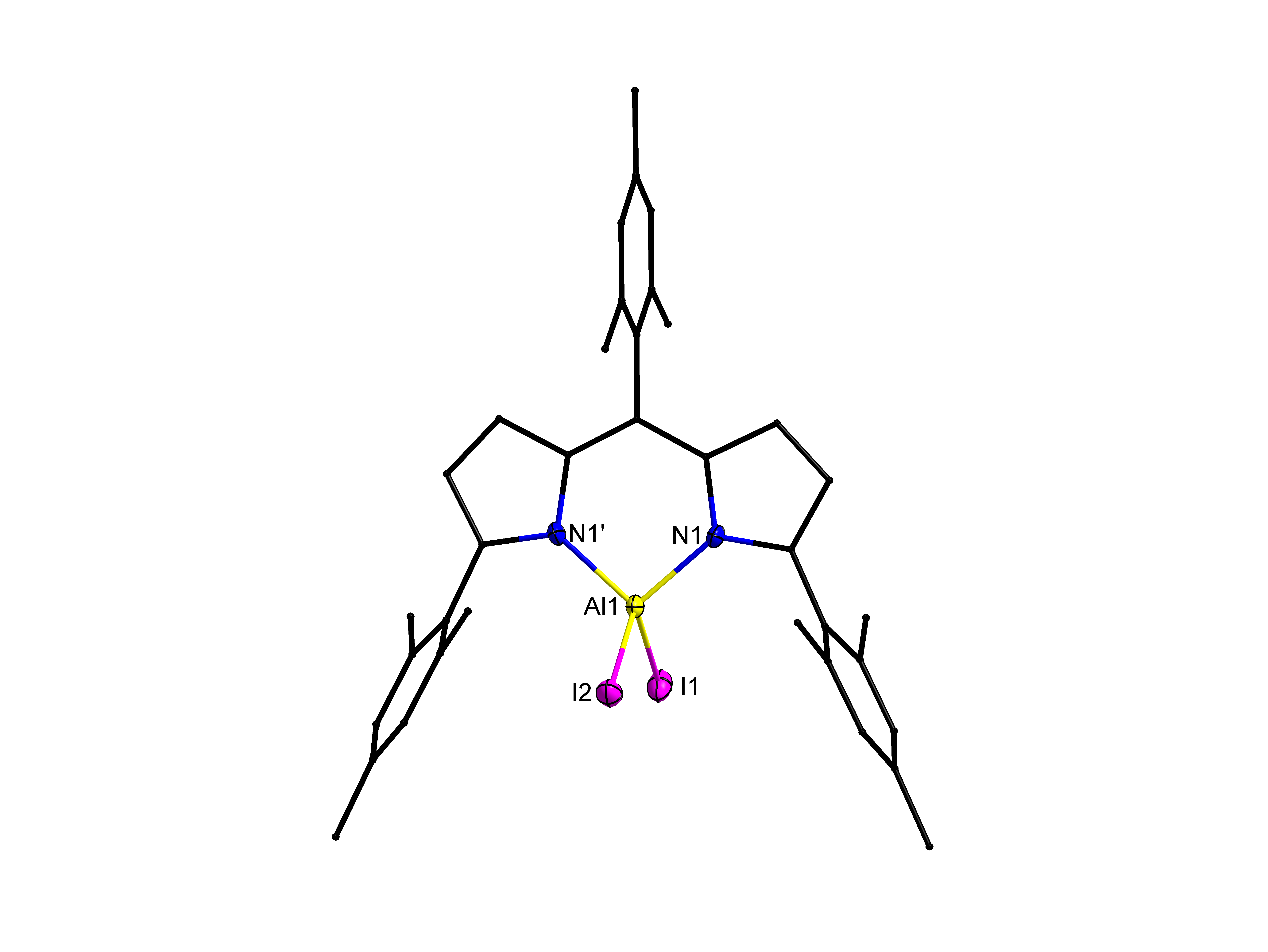

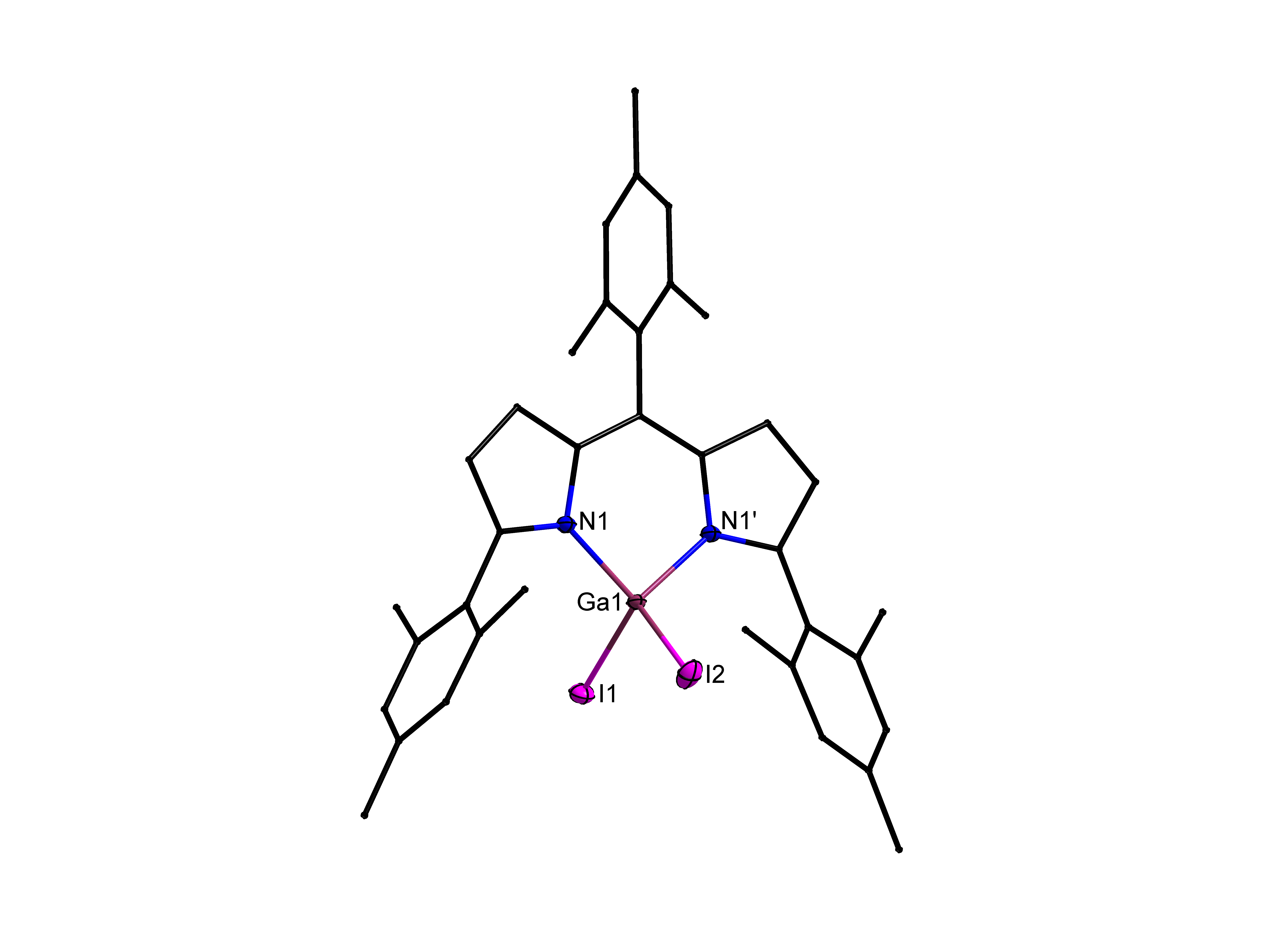

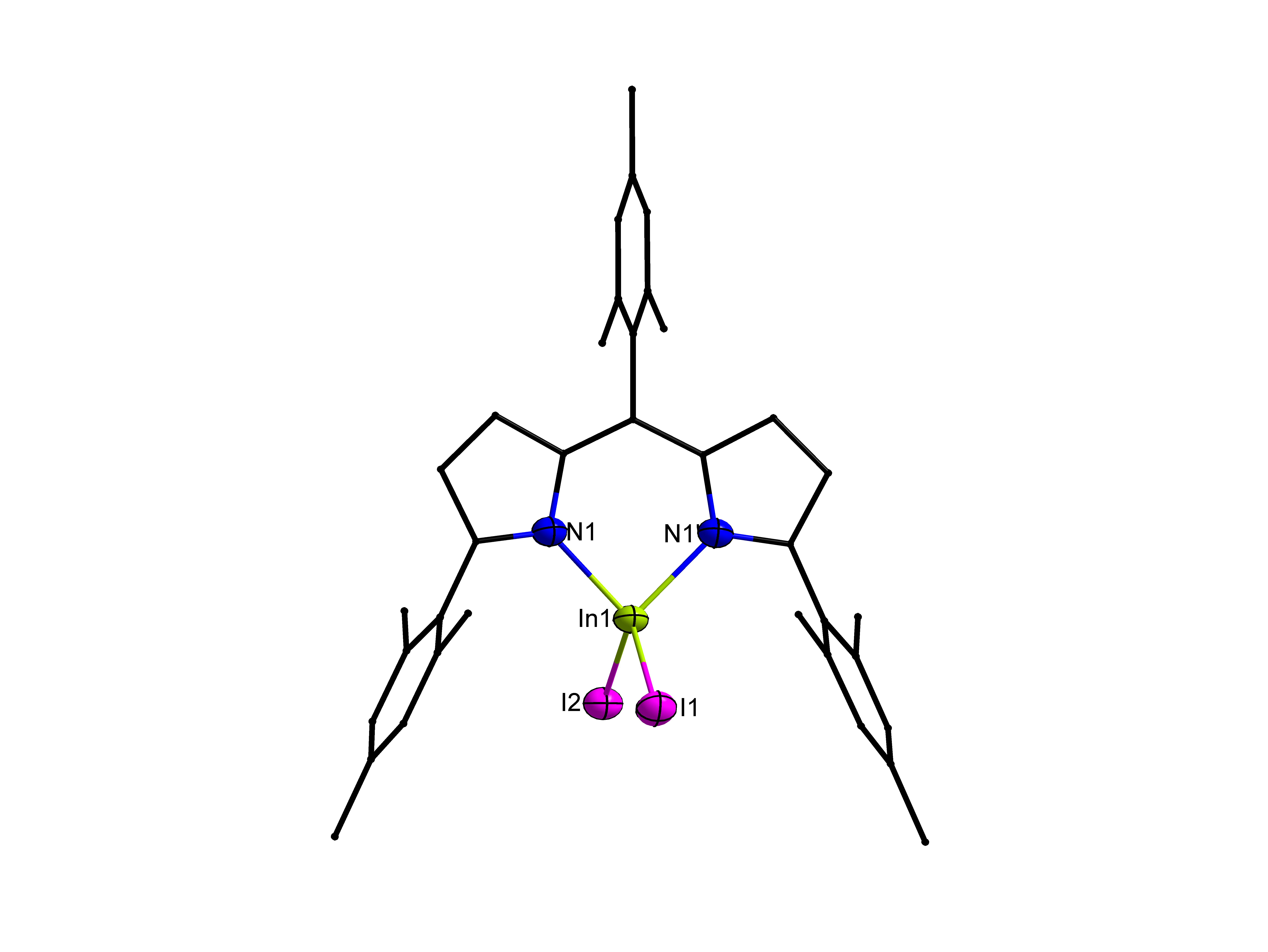


Figure S153: Solid state molecular structure for a) [(^Mes^DPM)AlI_2_] (3_I2_), b) [(^Mes^DPM)GaI_2_] (4_I2_) and c) [(^Mes^DPM)InI_2_] (5_I2_) with thermal ellipsoids set at the 50% probability level (color scheme: C = black, N = blue, Al = yellow, Ga = violet, In = light green, I = pink). Carbon atoms are depicted as wireframe, carbon-bound hydrogen atoms are omitted for clarity.

Table S6: Crystal data and structure refinement for [(^Mes^DPM)AlI_2_] (3_I2_), [(^Mes^DPM)GaI_2_] (4_I2_) and [(^Mes^DPM)InI_2_] (5_I2_).

| **Compound** | **3_I2_** | **4_I2_** (see also [10]) | **5_I2_** |
| --- | --- | --- | --- |
| Empirical formula | C_36_H_37_AlI_2_N_2_ | C_36_H_37_GaI_2_N_2_ | C_36_H_37_InI_2_N_2_ |
| Formular weight | 778.45 | 821.19 | 866.29 |
| Temperature / K | 100.0 | 100.0 | 100.0 |
| Crystal system | monoclinic | monoclinic | monoclinic |
| Space group | *P*2_1_/*m* | *P*2_1_/*m* | *P*2_1_/*m* |
| a / Å | 7.9622(9) | 7.9490(5) | 7.9174(1) |
| b / Å | 14.4230(15) | 14.4423(9) | 14.4199(3) |
| c / Å | 14.7257(15) | 14.7634(9) | 15.0339(2) |
| α / ° | 90 | 90 | 90 |
| β / ° | 101.495(4) | 101.630(2) | 101.480(1) |
| γ / ° | 90 | 90 | 90 |
| Volume / Å^3^ | 1657.2(3) | 1660.07(18) | 1682.05(5) |
| Z | 2 | 2 | 2 |
| Density (calculated) / g/cm^3^ | 1.560 | 1.643 | 1.710 |
| Absorption koefficient / mm^-1^ | 1.951 | 2.715 | 20.214 |
| F(000) | 772.0 | 808.0 | 844.0 |
| Crystal size resp. radius/ mm | 0.198 × 0.084 × 0.075 | 0.389 × 0.14 × 0.11 | 0.0976 |
| Radiation / nm | MoKα (λ = 0.71073) | MoKα (λ = 0.71073) | CuKα (λ = 1.54186) |
| 2Θ range for data collection / ° | 3.992 to 51.996 | 5.232 to 66.368 | 5.998 to 149.874 |
| Index ranges | -9 ≤ h ≤ 9, -17 ≤ k ≤ 17, -18 ≤ l ≤ 17 | -12 ≤ h ≤ 11, 0 ≤ k ≤ 22, 0 ≤ l ≤ 22 | -7 ≤ h ≤ 9, -17 ≤ k ≤ 17, -18 ≤ l ≤ 18 |
| Reflections collected | 37798 | 6601 | 30293 |
| Independent reflections | 3388 [*R*_int_ = 0.0647, *R*_sigma_ = 0.0295] | 6601 [*R*_int_ = merged, *R*_sigma_ = 0.0265] | 3555 [*R*_int_ = 0.0267, *R*_sigma_ = 0.0123] |
| Data/restraints/parameters | 3388/6/211 | 6601/0/212 | 3555/0/211 |
| Goodness-of-fit on F^2^ (GooF) | 1.261 | 1.042 | 1.125 |
| Final R indexes [I>=2σ (I)] | *R*_1_ = 0.0506, *wR*_2_ = 0.1263 | *R*_1_ = 0.0249, *wR*_2_ = 0.0521 | *R*_1_ = 0.0333, *wR*_2_ = 0.0984 |
| Final R indexes [all data] | *R*_1_ = 0.0603, *wR*_2_ = 0.1288 | *R*_1_ = 0.0329, *wR*_2_ = 0.0536 | *R*_1_ = 0.0337, *wR*_2_ = 0.0987 |
| Largest diff. Peak/hole / e Å^-3^ | 2.54/-1.74 | 0.87/-1.57 | 1.04/-0.76 |
| ccdc | 2413326 | - | 2413335 |

## 7.6 [(^Mes^DPM)M(Me)I] (M = Al (**3_MeI_**), Ga (**4_MeI_**), In (**5_MeI_**))

a) b) c)


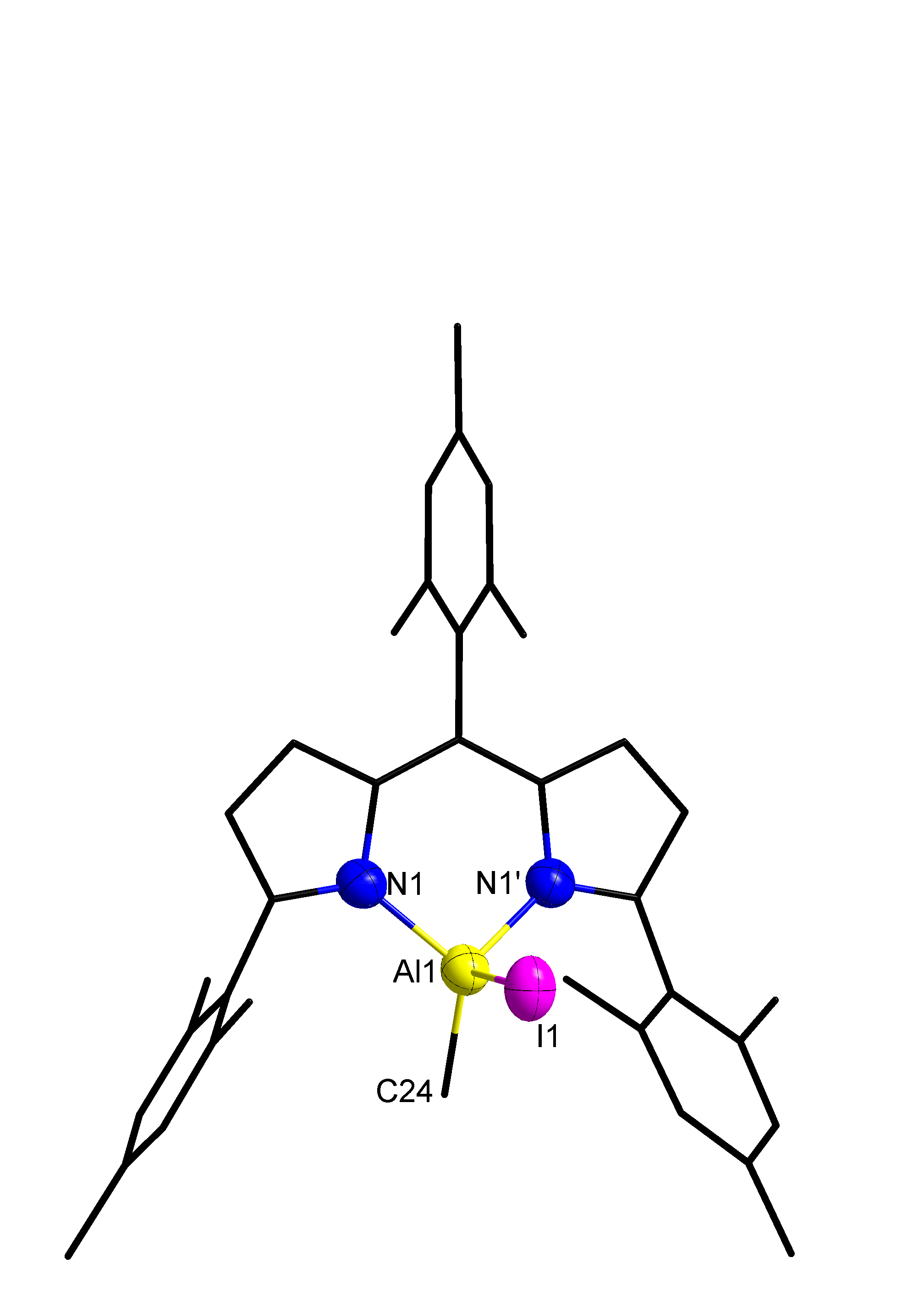

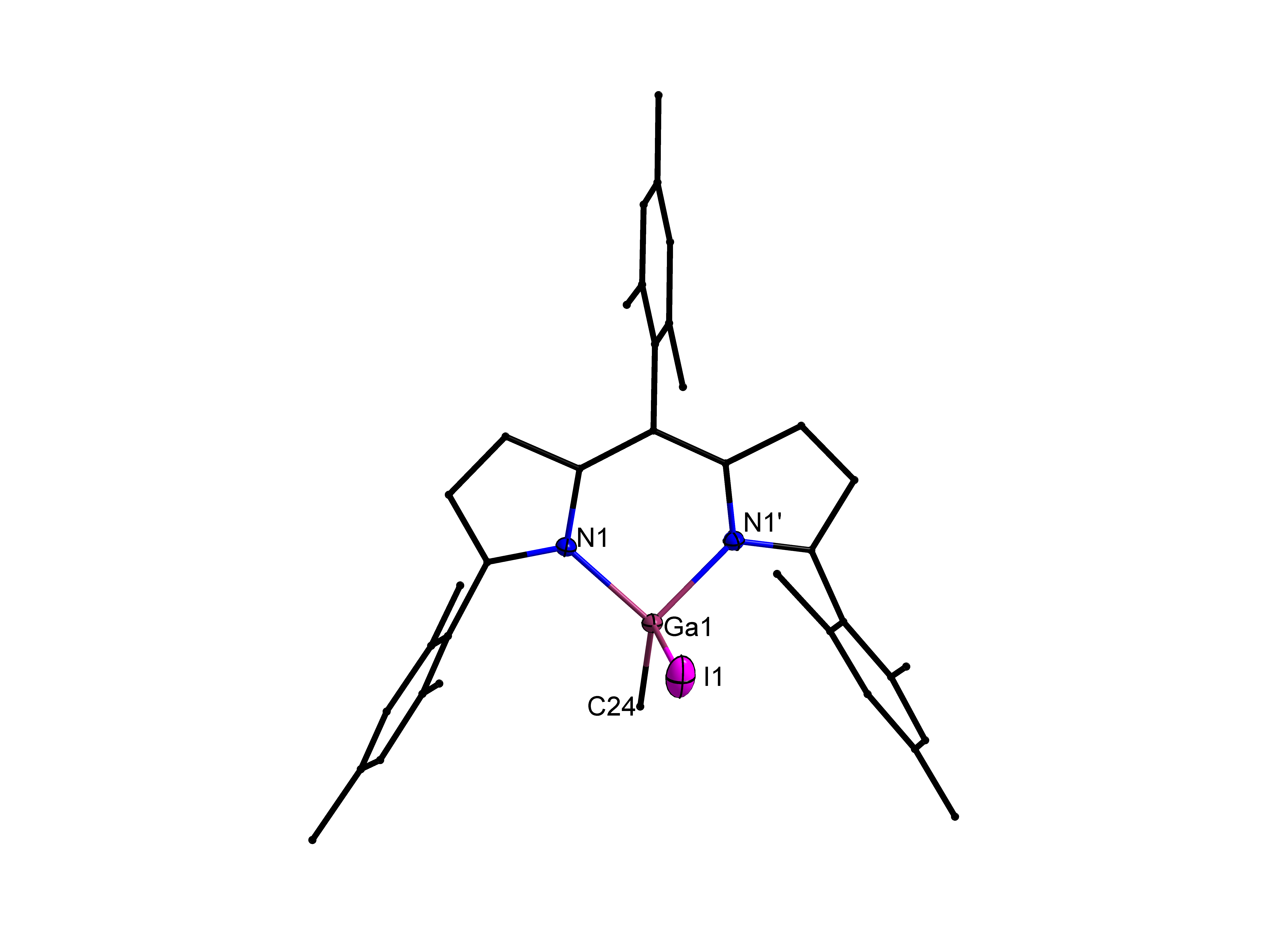

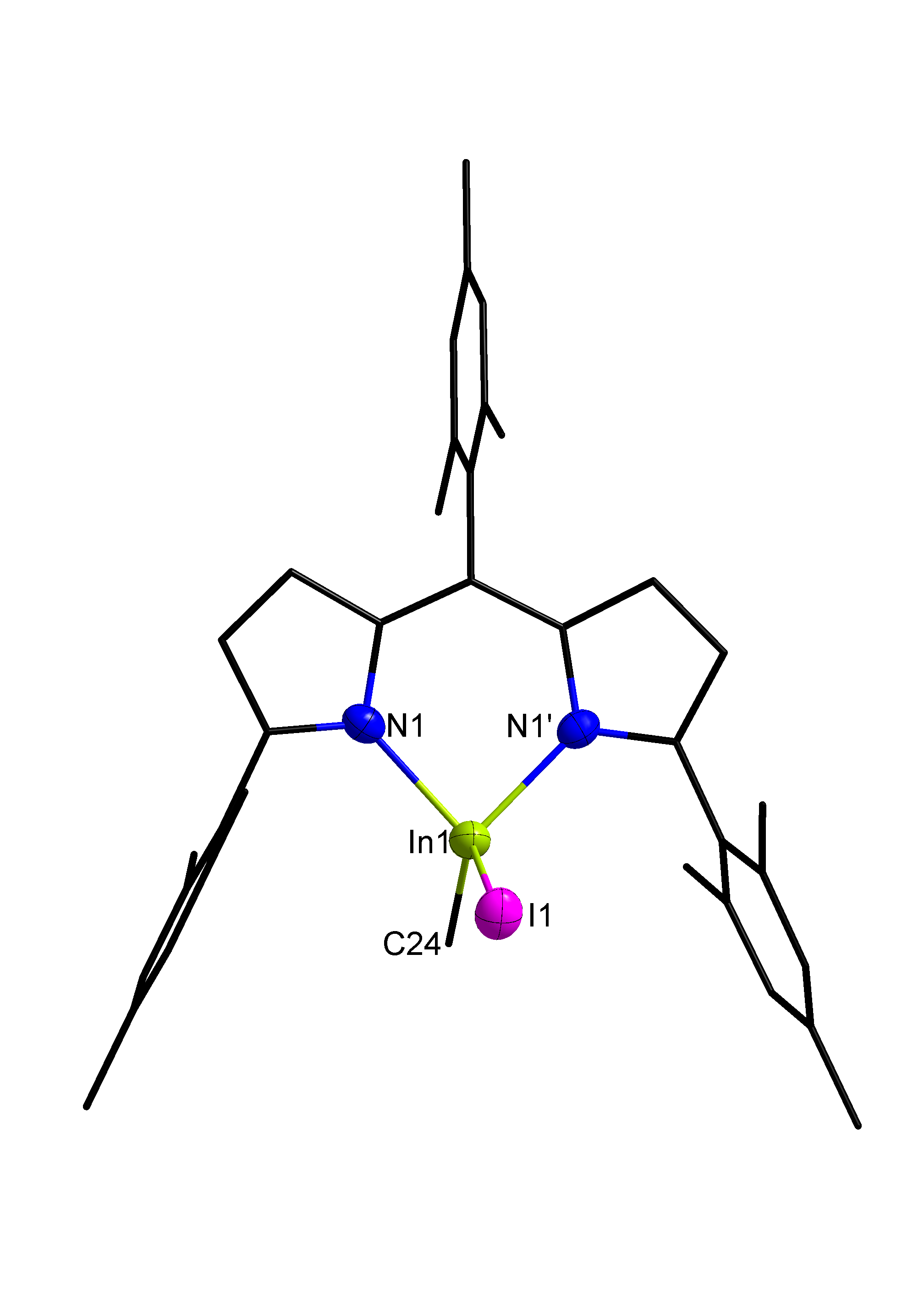


Figure S154: Solid state molecular structures for a) [(^Mes^DPM)Al(Me)I] (3_MeI_), b) [(^Mes^DPM)Ga(Me)I] (4_MeI_) and c) [(^Mes^DPM)In(Me)I] (5_MeI_) with thermal ellipsoids set at the 50% probability level (color scheme: C = black, N = blue, Ga = violet, In = light green, I = pink). Carbon atoms are depicted as wireframe, carbon-bound hydrogen atoms and disorders are omitted for clarity.

Table S7: Crystal data and structure refinements for [(^Mes^DPM)Al(Me)I] (3_MeI_), [(^Mes^DPM)Ga(Me)I] (4_MeI_) and [(^Mes^DPM)In(Me)I] (5_MeI_).

| **Compound** | **3_MeI_** | **4_MeI_** | **5_MeI_** |
| --- | --- | --- | --- |
| Empirical formula | C_37_H_40_AlIN_2_ | C_37_H_40_GaIN_2_ | C_37_H_40_IInN_2_ |
| Formular weight | 666.59 | 709.33 | 754.43 |
| Temperature / K | 100.0 | 100.0 | 100 |
| Crystal system | monoclinic | monoclinic | monoclinic |
| Space group | *P*2_1_/*m* | *P*2_1_/*m* | *P*2_1_/*m* |
| a / Å | 8.1269(3) | 8.1059(4) | 7.9617(2) |
| b / Å | 15.0681(5) | 15.0195(8) | 14.3970(2) |
| c / Å | 13.8832(7) | 13.9033(7) | 14.9085(3) |
| α / ° | 90 | 90.0 | 90 |
| β / ° | 103.504(4) | 103.555(2) | 102.179(2) |
| γ / ° | 90 | 90.0 | 90 |
| Volume / Å^3^ | 1653.09(12) | 1645.53(15) | 1670.42(6) |
| Z | 2 | 2 | 2 |
| Density (calculated) / g/cm^3^ | 1.339 | 1.432 | 1.500 |
| Absorption koefficient / mm^-1^ | 8.060 | 1.801 | 13.098 |
| F(000) | 684.0 | 720.0 | 756.0 |
| Crystal size resp. radius/ mm | 0.041 | 0.264 × 0.107 × 0.091 | 0.0295 |
| Radiation / nm | CuKα (λ = 1.54186) | MoKα (λ = 0.71073) | CuKα (λ = 1.54186) |
| 2Θ range for data collection / ° | 6.548 to 133.182 | 4.054 to 56.99 | 6.064 to 130.176 |
| Index ranges | -9 ≤ h ≤ 9, -10 ≤ k ≤ 17, -16 ≤ l ≤ 16 | -10 ≤ h ≤ 10, -20 ≤ k ≤ 20, -18 ≤ l ≤ 18 | -7 ≤ h ≤ 9, -16 ≤ k ≤ 14, -17 ≤ l ≤ 17 |
| Reflections collected | 20112 | 43181 | 32432 |
| Independent reflections | 3043 [*R*_int_ = 0.0979, *R*_sigma_ = 0.0783] | 4332 [*R*_int_ = 0.0423, *R*_sigma_ = 0.0229] | 2980 [*R*_int_ = 0.0800, *R*_sigma_ = 0.0395] |
| Data/restraints/parameters | 3043/7/216 | 4332/0/211 | 2980/4/221 |
| Goodness-of-fit on F^2^ (GooF) | 0.953 | 1.206 | 1.222 |
| Final R indexes [I>=2σ (I)] | *R*_1_ = 0.0954, *wR*_2_ = 0.2342 | *R*_1_ = 0.0410, *wR*_2_ = 0.1006 | *R*_1_ = 0.0678, *wR*_2_ = 0.1640 |
| Final R indexes [all data] | *R*_1_ = 0.1180, *wR*_2_ = 0.2438 | *R*_1_ = 0.0481, *wR*_2_ = 0.1026 | *R*_1_ = 0.0786, *wR*_2_ = 0.1678 |
| Largestdiff. Peak/hole / e Å^-3^ | 3.18/-1.56 | 1.32/-1.33 | 1.90/-1.41 |
| ccdc | 2413327 | 2413324 | 2413330 |

## 7.7 [(^Mes^DPM)M(Me)Br] (M = Al (**3_MeBr_**), Ga (**4_MeBr_**), In (**5_MeBr_**))

a) b) c)


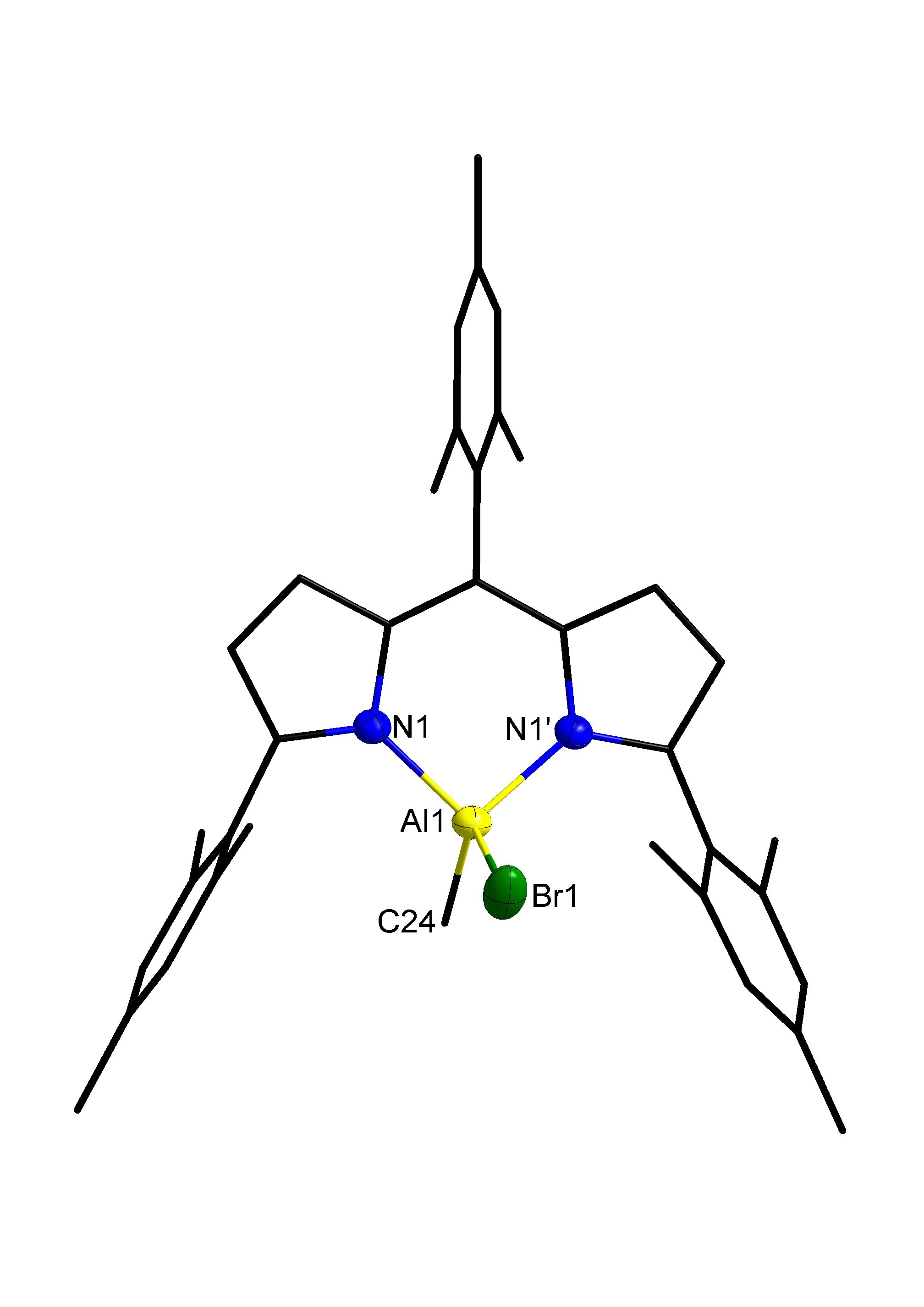

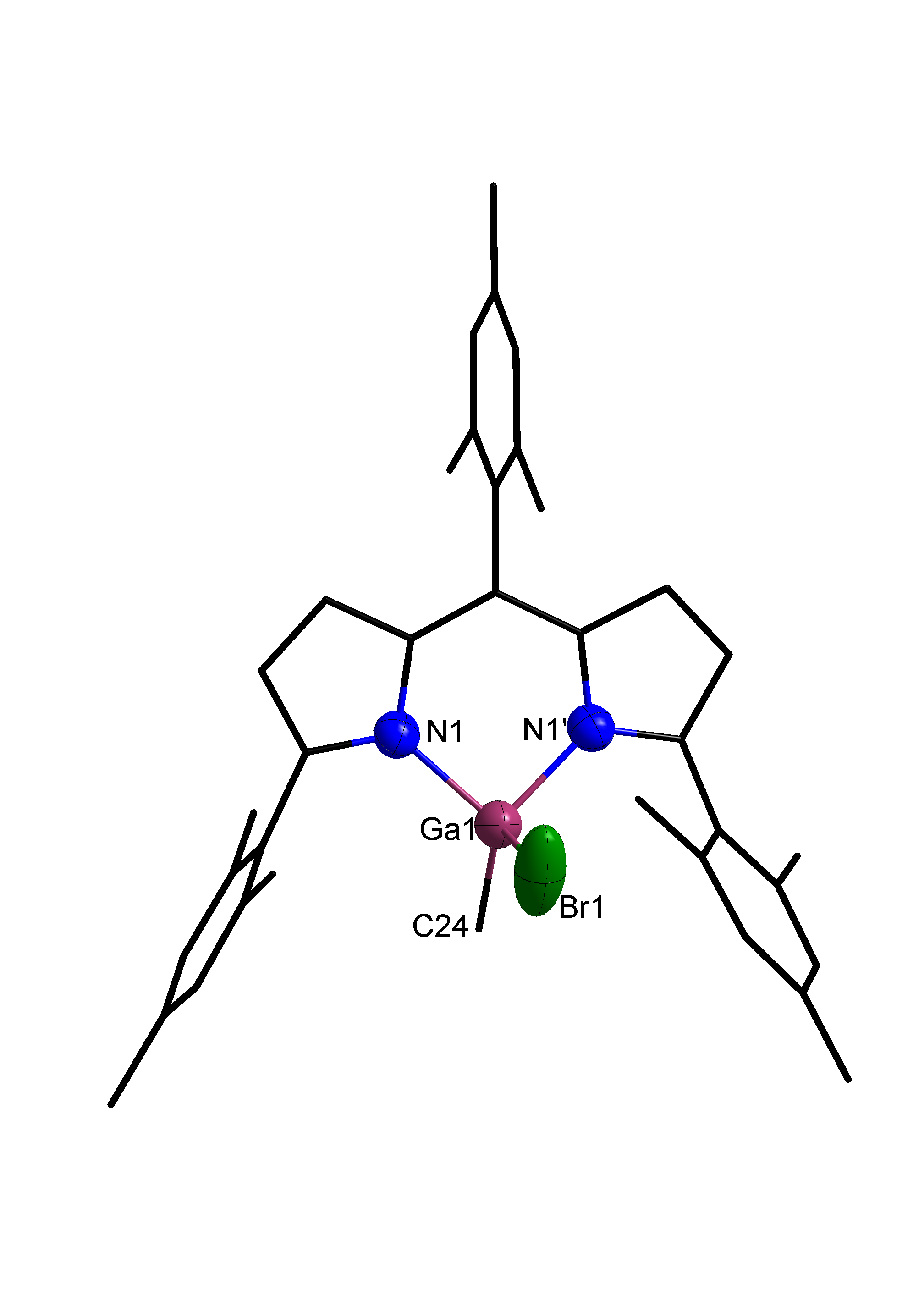

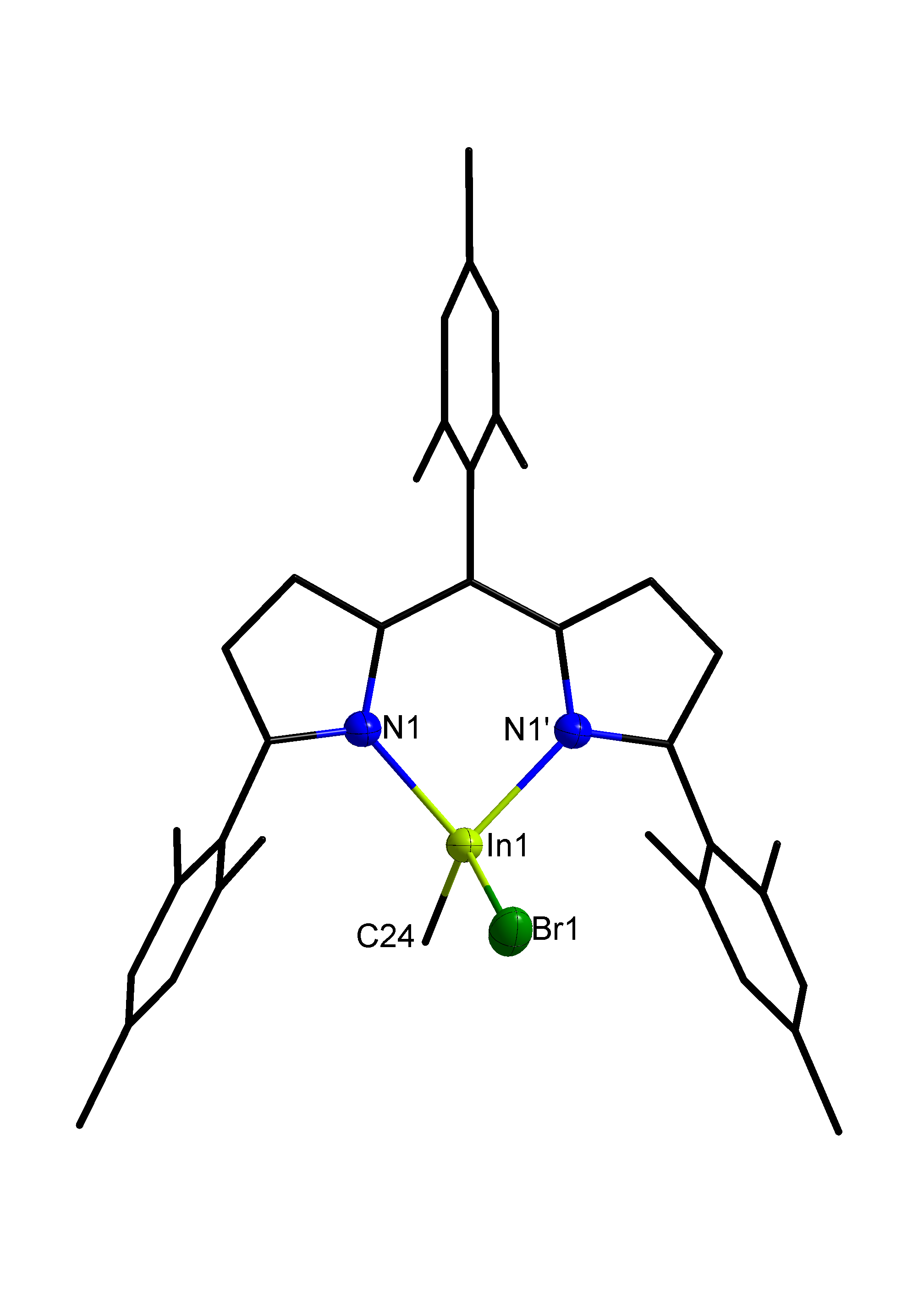


Figure S155: Solid state molecular structures for a) [(^Mes^DPM)Al(Me)Br] (3_MeBr_), b) [(^Mes^DPM)Ga(Me)Br] (4_MeBr_) and c) [(^Mes^DPM)In(Me)Br] (5_MeBr_) with thermal ellipsoids set at the 50% probability level (color scheme: C = black, N = blue, Al = yellow, Ga = violet, In = light green, I = pink). Carbon atoms are depicted as wireframe, carbon-bound hydrogen atoms and disorders are omitted for clarity.

Table S8: Crystal data and structure refinements for [(^Mes^DPM)Al(Me)Br] (3_MeBr_), [(^Mes^DPM)Ga(Me)Br] (4_MeBr_) and [(^Mes^DPM)In(Me)Br] (5_MeBr_).

| **Compound** | **3_MeBr_** | **4_MeBr_** | **5_MeBr_** |
| --- | --- | --- | --- |
| Empirical formula | C_37_H_40_AlBrN_2_ | C_37_H_40_GaBrN_2_ | C_37_H_40_BrInN_2_ |
| Formular weight | 619.60 | 662.34 | 707.44 |
| Temperature / K | 100.0 | 100.0 | 100.0 |
| Crystal system | monoclinic | monoclinic | monoclinic |
| Space group | *P*2_1_/*m* | *P*2_1_/*m* | *P*2_1_/*m* |
| a / Å | 8.0702(2) | 8.0914(4) | 8.0028(4) |
| b / Å | 14.5182(3) | 14.8135(5) | 14.4507(5) |
| c / Å | 14.3887(4) | 14.0930(7) | 14.7155(7) |
| α / ° | 90.0 | 90.0 | 90 |
| β / ° | 103.937(2) | 104.661(4) | 103.782(4) |
| γ / ° | 90.0 | 90.0 | 90 |
| Volume / Å^3^ | 1636.22(7) | 1634.21(13) | 1652.79(13) |
| Z | 2 | 2 | 2 |
| Density (calculated) / g/cm^3^ | 1.258 | 1.346 | 1.422 |
| Absorption coefficient / mm^-1^ | 2.166 | 2.777 | 7.321 |
| F(000) | 648.0 | 684.0 | 720.0 |
| Crystal radius / mm | 0.0349 | 0.106 | 0.0488 |
| Radiation / nm | CuKα (λ = 1.54186) | CuKα (λ = 1.54186) | CuKα (λ = 1.54186) |
| 2Θ range for data collection / ° | 6.33 to 130.178 | 8.814 to 133.172 | 6.184 to 133.194 |
| Index ranges | -9 ≤ h ≤ 9, -12 ≤ k ≤ 17, -15 ≤ l ≤ 16 | -9 ≤ h ≤ 6, -17 ≤ k ≤ 17, -16 ≤ l ≤ 16 | -9 ≤ h ≤ 8, -13 ≤ k ≤ 17, -17 ≤ l ≤ 17 |
| Reflections collected | 22691 | 19154 | 38190 |
| Independent reflections | 2915 [*R*_int_ = 0.0712, *R*_sigma_ = 0.0566] | 3010 [*R*_int_ = 0.0555, *R*_sigma_ = 0.0289] | 3057 [*R*_int_ = 0.0464, *R*_sigma_ = 0.0208] |
| Data/restraints/parameters | 2915/0/212 | 3010/0/211 | 3057/6/214 |
| Goodness-of-fit on F^2^ (GooF) | 0.932 | 1.065 | 1.033 |
| Final R indexes [I>=2σ (I)] | *R*_1_ = 0.0387, *wR*_2_ = 0.0970 | *R*_1_ = 0.0640, *wR*_2_ = 0.1776 | *R*_1_ = 0.0428, *wR*_2_ = 0.1138 |
| Final R indexes [all data] | *R*_1_ = 0.0625, *wR*_2_ = 0.1024 | *R*_1_ = 0.0715, *wR*_2_ = 0.1840 | *R*_1_ = 0.0441, *wR*_2_ = 0.1148 |
| Largest diff. Peak/hole / e Å^-3^ | 0.69/-0.85 | 0.88/-0.87 | 3.19/-1.19 |
| ccdc | 2413323 | 2413319 | 2413328 |

## 7.8 [(^Mes^DPM)M(Me)Cl] (M = Al (**3_MeCl_**), Ga (**4_MeCl_**), In (**5_MeCl_**))

a) b) c)


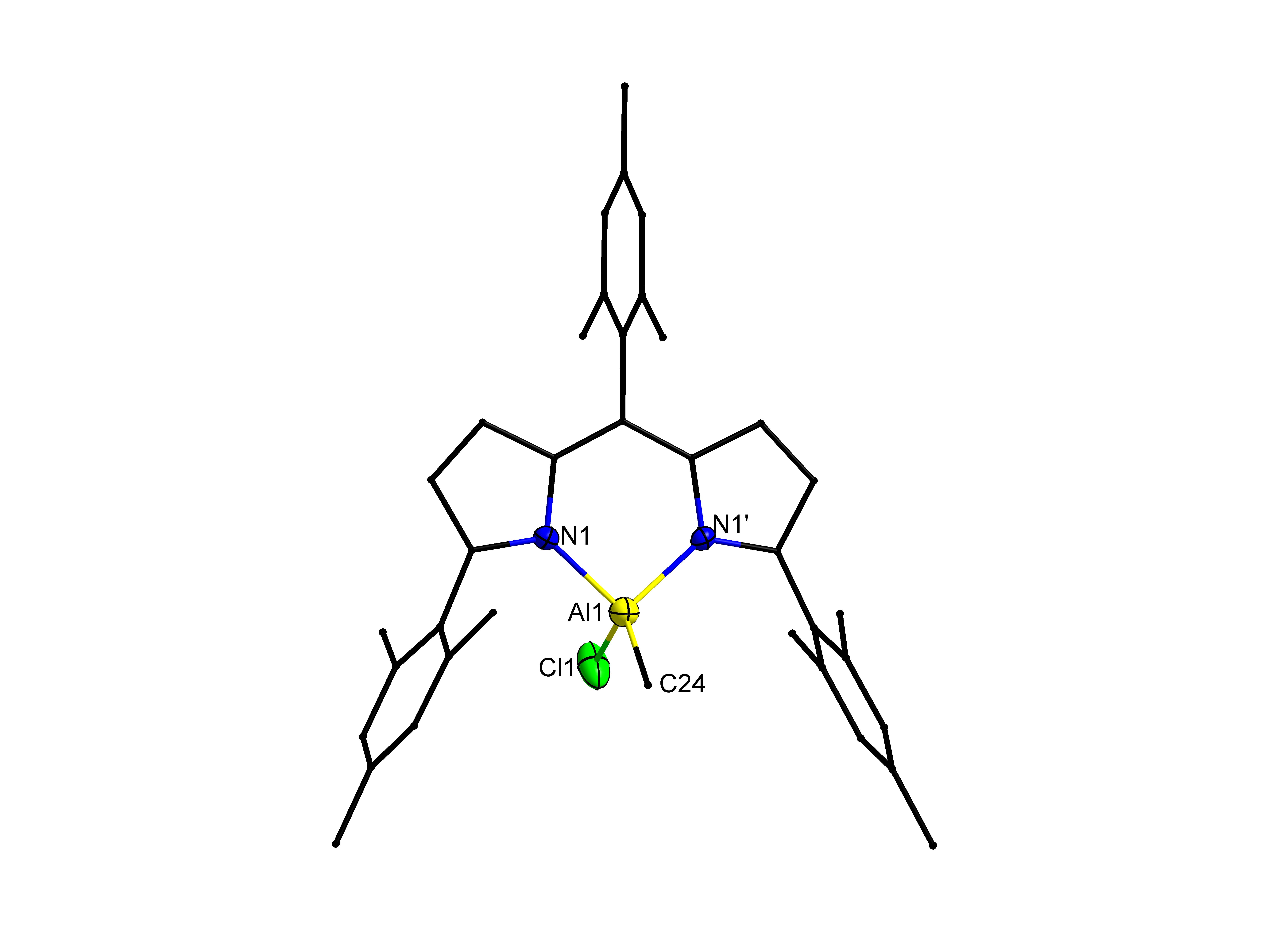

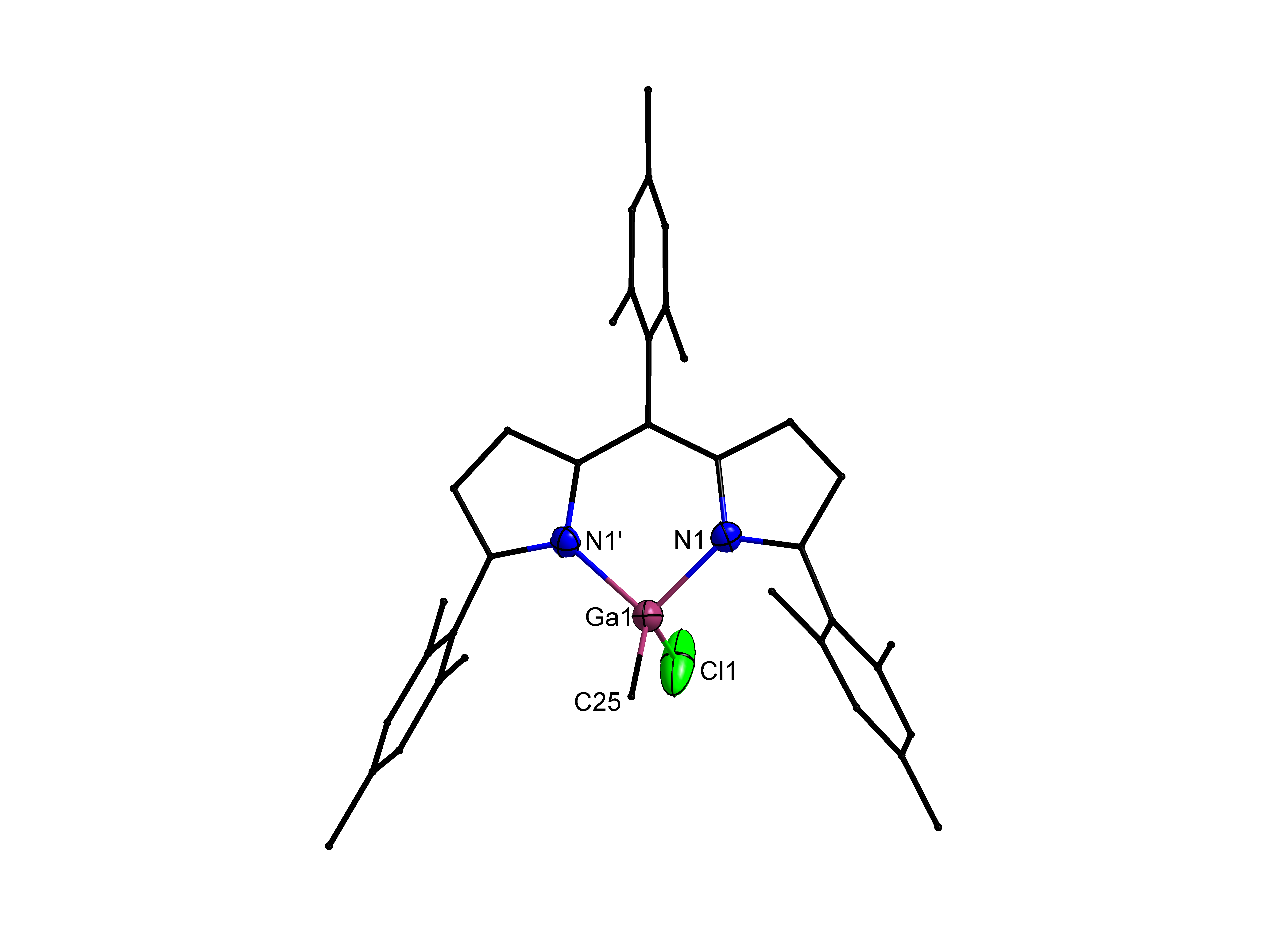

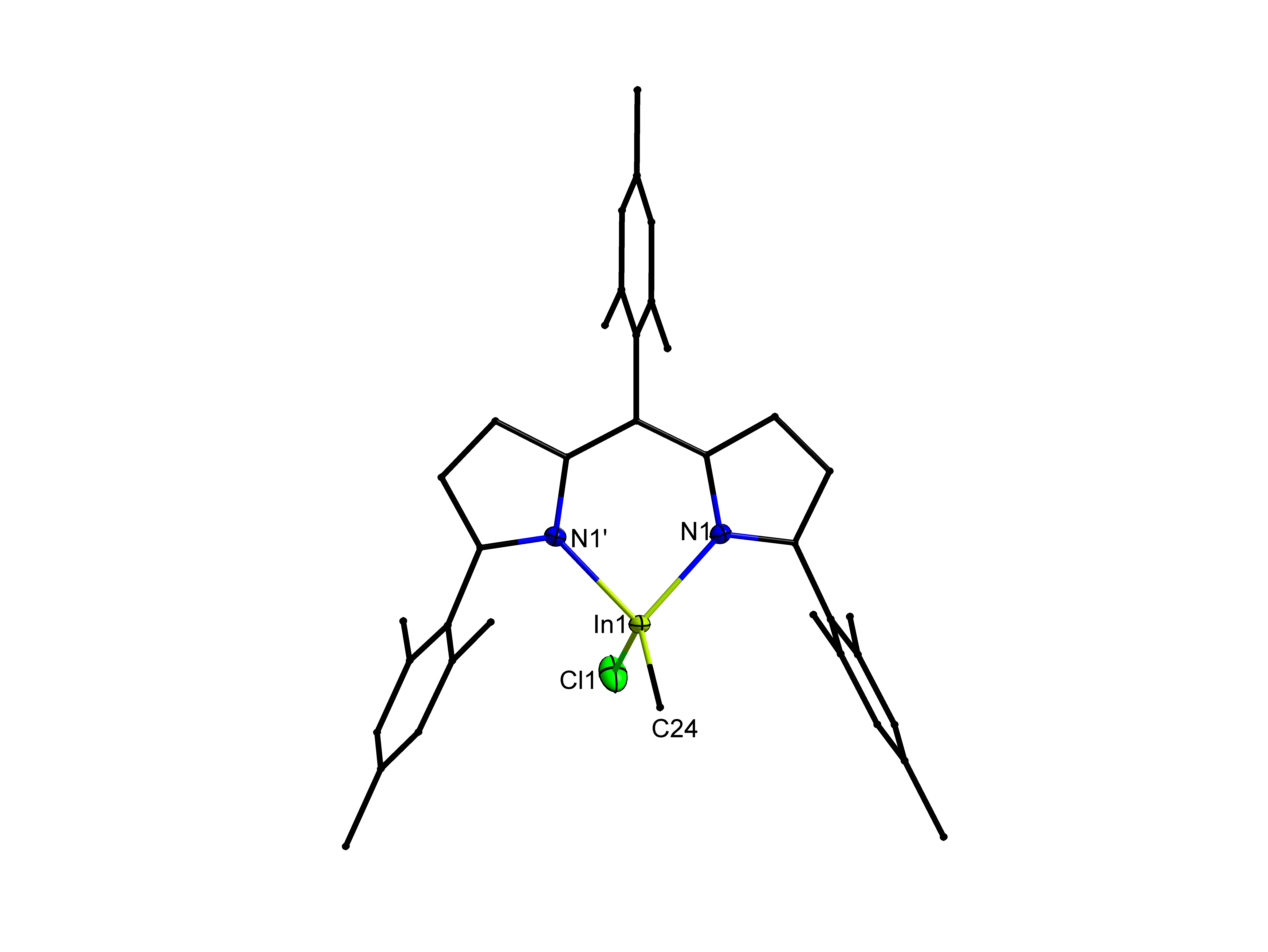


Figure S156: Solid state molecular structures for a) [(^Mes^DPM)Al(Me)Cl] (3_MeCl_), b) [(^Mes^DPM)Ga(Me)Cl] (4_MeCl_) and c) [(^Mes^DPM)In(Me)Cl] (5_MeCl_) with thermal ellipsoids set at the 50% probability level (color scheme: C = black, N = blue, Al = yellow, Ga = violet, In = light green, I = pink). Carbon atoms are depicted as wireframe, carbon-bound hydrogen atoms are omitted for clarity.

Table S9: Crystal data and structure refinements for [(^Mes^DPM)Al(Me)Cl] (3_MeCl_), [(^Mes^DPM)Ga(Me)Cl] (4_MeCl_) and [(^Mes^DPM)In(Me)Cl] (5_MeCl_).

| **Compound** | **3_MeCl_** | **4_MeCl_** | **5_MeCl_** |
| --- | --- | --- | --- |
| Empirical formula | C_37_H_40_AlClN_2_ | C_37_H_40_GaClN_2_ | C_37_H_40_ClInN_2_ |
| Formular weight | 575.14 | 617.88 | 662.98 |
| Temperature / K | 100.0 | 100.0 | 100.0 |
| Crystal system | monoclinic | monoclinic | monoclinic |
| Space group | *P*2_1_/*m* | *P*2_1_/*m* | P2_1_/m |
| a / Å | 8.0658(6) | 8.0885(3) | 8.0462(5) |
| b / Å | 14.4822(10) | 14.6710(4) | 14.4751(8) |
| c / Å | 14.3200(10) | 14.1768(5) | 14.5511(8) |
| α / ° | 90.0 | 90.0 | 90.0 |
| β / ° | 104.385(3) | 105.116(3) | 104.854(2) |
| γ / ° | 90.0 | 90.0 | 90.0 |
| Volume / Å^3^ | 1620.3(2) | 1624.10(10) | 1638.12(16) |
| Z | 2 | 2 | 2 |
| Density (calculated) / g/cm^3^ | 1.179 | 1.263 | 1.344 |
| Absorption coefficient / mm^-1^ | 0.172 | 0.957 | 0.830 |
| F(000) | 612.0 | 648.0 | 684.0 |
| Crystal size resp. radius / mm | 0.219 × 0.161 × 0.088 | 0.114 | 0.103 × 0.071 × 0.071 |
| Radiation / nm | MoKα (λ = 0.71073) | MoKα (λ = 0.71073) | MoKα (λ = 0.71073) |
| 2Θ range for data collection / ° | 4.066 to 54 | 5.216 to 55.998 | 4.038 to 68.55 |
| Index ranges | -10 ≤ h ≤ 10, -18 ≤ k ≤ 17, -18 ≤ l ≤ 18 | -10 ≤ h ≤ 10, -19 ≤ k ≤ 16, -18 ≤ l ≤ 18 | -12 ≤ h ≤ 12, -22 ≤ k ≤ 21, -22 ≤ l ≤ 18 |
| Reflections collected | 35440 | 23215 | 44901 |
| Independent reflections | 3687 [*R*_int_ = 0.0577, *R*_sigma_ = 0.0317] | 4062 [*R*_int_ = 0.0531, *R*_sigma_ = 0.0472] | 6699 [*R*_int_ = 0.0483, *R*_sigma_ = 0.0488] |
| Data/restraints/parameters | 3687/6/212 | 4062/0/211 | 6699/0/212 |
| Goodness-of-fit on F^2^ (GooF) | 1.097 | 0.966 | 1.079 |
| Final R indexes [I>=2σ (I)] | *R*_1_ = 0.0649, *wR*_2_ = 0.1489 | *R*_1_ = 0.0416, *wR*_2_ = 0.1063 | *R*_1_ = 0.0359, *wR*_2_ = 0.0647 |
| Final R indexes [all data] | *R*_1_ = 0.0795, *wR*_2_ = 0.1542 | *R*_1_ = 0.0556, *wR*_2_ = 0.1094 | *R*_1_ = 0.0583, *wR*_2_ = 0.0689 |
| Largest diff. Peak/hole / e Å^-3^ | 1.18/-1.18 | 0.56/-0.70 | 0.69/-1.27 |
| ccdc | 2413321 | 2413364 | 2413334 |

# 8. Literature:

[1] E. R. King, T. A. Betley, *Inorg. Chem.* **2009**, *48*, 2361–2363.

[2] M. R. Willcott, *J. Am. Chem. Soc.* **2009**, *131*, 13180–13180.

[3] Bruker. SAINT. Crystallography Software Suite, Bruker AXS Inc., Madison, WI, USA, **2016**.

[4] X-AREA: Program for the Acquisition and Analysis of Data, Version 1.90, STOE & Cie GmbH: Darmstadt, **2020**.

[5] O. V. Dolomanov, L. J. Bourhis, R. J. Hildea, J. . A. K. Howard, H. Puschmann, *Olex2 A Completete Structure Solution, Refinement and Analysis Program. J. Appl. Crystallogr.* **2009**, *42*, 339–341.

[6] G. M. Sheldrick, *SHELXL14; Program for the Refinement of Crystal Structures* **2014**, *Universität Göttingen*.

[7] W. T. Pennington, *J. Appl. Crystallogr.* **1999**, *32*, 1028–1029.

[8] J. Valenta, *AIP Adv.* **2018**, *8*, 105123.

[9] A. M. Brouwer, *Pure Appl. Chem.* **2011**, *83*, 2213–2228.

[10] T. Richter, S. Thum, O. P. E. Townrow, J. Langer, M. Wiesinger, S. Harder, *J. Organomet. Chem.* **2024**, *1021*, 123356.
